# Design and synthesis of 3,4-*seco*-lupane triterpene-tryptamine derivatives and revealing their anti-bladder cancer mechanisms by combining TCGA and transcriptomic approaches

**DOI:** 10.1038/s41598-025-04855-y

**Published:** 2025-06-05

**Authors:** Qinglong Chi, Hongbo Teng, Yaru Zhao, Xv Wang, Jiexin Zhang, Huiyue Shen, Xuan He, Yan Zhao, Chunxi Wang

**Affiliations:** 1 Department of Urology, The First Hospital of Jilin University, Changchun, 130021 Jilin China; 2 College of Chinese Medicinal Materials, Jilin Agricultural University, Changchun, Jilin China

**Keywords:** Bladder cancer, *seco*-lupane triterpene derivatives, Transcriptomics, TCGA, Cancer genomics, Urological cancer

## Abstract

Bladder cancer is the most common malignant tumor of the urinary tract. In this study, 90 lupane triterpene derivatives, previously synthesized in the laboratory, were systematically evaluated for their potential effects against bladder cancer by cytotoxicity screening against five urinary tumor cell lines. Bioinformatics and molecular dynamics methods were used to investigate the mechanism of action of compound 27 in depth. Most of the derivatives effectively inhibited tumor cell growth, and structure–activity relationship analysis revealed that introducing an indole moiety significantly enhanced the biological activity. The peak activity was reached when the dibromoalkyl chain length was C = 5 (IC_50_ = 1.121 μM). By integrating transcriptomic data and TCGA findings, we identified 11 key targets, among which *DUSP5* and *SCG2* showed significant differential expression. Further analysis revealed meaningful insights into the clinical association, 10-year survival prognosis, and immune infiltration. The present study further clarified the effects of compound 27 on the expression of *DUSP5* and *SCG2* in tumor cells after treatment by a combination of RNA-seq and RT-qPCR. Molecular docking confirmed the stable binding of compound 27 to *DUSP5*, which was confirmed by molecular dynamics simulations. Compound 27 inhibited bladder cancer progression by upregulating *DUSP5* expression and negatively regulating the p38 MAPK pathway, modulating the immune response and promoting apoptosis.

## Introduction

Bladder cancer refers to a malignant tumor that occurs on the bladder mucosa. It is a tumor of the cells lining the inner wall of the bladder and the most common malignant tumor in the urinary system^1^. According to the latest GLOBOCAN data, the incidence of bladder cancer in Asia is 35.1%, with a mortality rate of 41.9%^2^. Most patients have non-muscle-invasive bladder cancer, and surgery is the primary treatment method^3^. However, patients often have a high risk of recurrence after surgery. Gemcitabine^4^ is a commonly used clinical drug for chemotherapy, but its drug resistance^5^ and toxicity^6^ severely limit its clinical application. Bladder cancer is a histologically and clinically heterogeneous disease, and most cases exhibit highly aggressive behavior, posing unique challenges to diagnosis and treatment^7^. Its main characteristic is a high recurrence rate^8^. Therefore, there is an urgent need to explore new and effective treatment drugs and regimens. The development of bladder cancer is mainly closely related to the proliferation and metastasis of tumor cells. In-depth studies have shown that cancer cells may proliferate by regulating the cell cycle^9^, apoptosis^10^, and other related factors^11,12^. For example, oxidized picloram inhibits the proliferation of human bladder cancer cells by inducing apoptosis and cell cycle arrest^13^.

*Acanthopanax sessiliflorus* (Rupr. et Maxim.) Seem. is a medicinal plant primarily distributed in Heilongjiang, Jilin, Liaoning, Hebei, and Shanxi provinces in China, and it has shown specific potential in anti-tumor activity^14^. Preliminary studies by our research group found that among the medicinal plants of the genus *Acanthopanax*, the lupane triterpenoid compound chiisanoside is abundant and exhibits multiple pharmacological activities, including significant anti-tumor, anti-inflammatory, and antibacterial activities^15,16^. Specifically, chiisanoside, as a triterpenoid saponin, has been reported to possess anti-cancer, hepatoprotective, and anti-diabetic activities and effects on lymphocyte proliferation^17^. Regarding anti-tumor activity, chiisanoside has shown the ability to inhibit tumor growth in H22 xenografted mouse models by promoting apoptosis and inhibiting angiogenesis^18^. Furthermore, chiisanoside has been studied for its application in predicting anti-tumor quality markers, analyzing mechanisms of action, and preparing derivatives^19^. At the same time, further studies have shown that the 3,4-*seco*-lupane triterpene derivatives (chiisanogenin) without a glycosyl group are significantly superior to the triterpenoid saponin (chiisanoside) with a glycosyl group in terms of anti-cancer activity^20^. These studies have revealed that chiisanoside and its derivatives in *Acanthopanax sessiliflorus* have significant research and application value in anti-tumor activity. Consequently, our research focus has shifted towards the activity screening of a series of derivatives based on lead compounds, such as chisanogenin and compound MH, to develop more effective drugs and enhance the utilization efficiency of *Acanthopanax* plant resources.

Transcriptomics research focuses on the regulatory mechanisms of genes at the transcriptional and RNA levels^21^. Analyzing differences in gene expression and their effects on cellular phenotype and function by sequencing technology can help to reveal the underlying mechanisms of specific regulatory genes under pathological conditions^22^. After selecting effective derivatives, we will assess their impact on the mechanisms of action against bladder cancer using bioinformatics analysis, molecular docking techniques, and molecular dynamics simulations. These studies will not only blaze new trails in the research of anti-cancer drugs but also provide a robust theoretical basis for the clinical treatment of bladder cancer.

## Results and discussion

### Chemistry

#### Preparation of derivatives

All 90 derivatives were structurally characterized by ^1^H NMR, ^13^C NMR, and HRMS and were detected by HPLC with purity over 95%. The characterization data were consistent with the predicted structures.

**Fig. 1:**
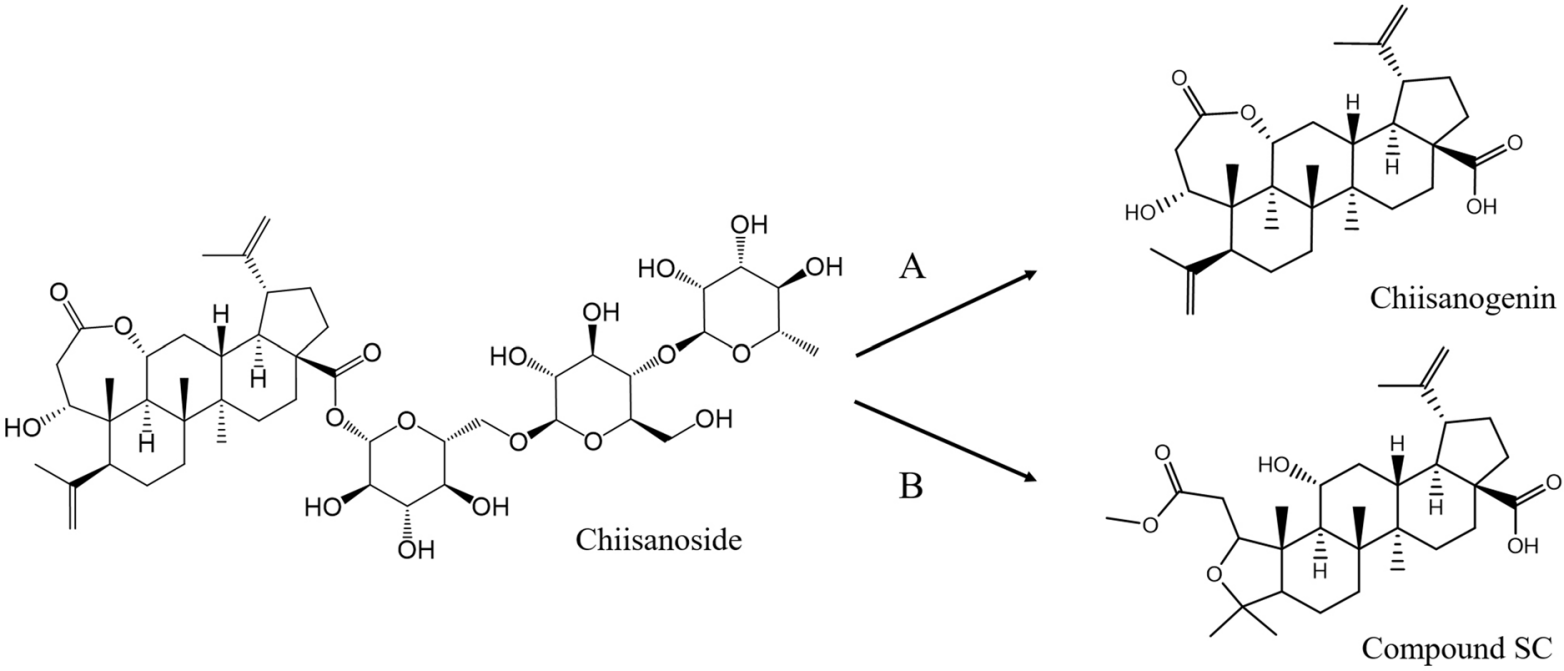
(**A**) 10% NaOH, MeOH, Reflux, 4 h; HCl, Stir, 2 h (**B**) HCl (3 mol/L), MeOH, Reflux, 2 h.

Preliminary experimental results showed that chisanogenin and compound SC are natural products of short-stemmed Wujia. However, their low content limits their further exploitation. Preliminary hydrolysis of echinacoside, which is found in considerable amounts in the leaves of S. pentaphyllum, using an alkali solution enabled us to obtain a large amount of chisanogenin and prepare a large quantity of compound SC by ester exchange reaction, as shown in Fig. 1. Echinacoside, chisanogenin, and compound SC have all been structurally determined by NMR^23^. Our previous studies have reported the structural characterization of derivatives 1–7, 10–16, 19–25, 28–34, 37–43, 46–52, 55–61, 64–70, 73–79, and 82–88^24^. Supporting Information Figs. S1–S40 show the specific characterization data and purity of derivatives 8, 9, 17, 18, 26, 27, 35, 36, 44, 45, 53, 54, 62, 63, 71, 72, 80, 81, 89, 90 synthesized based on the further optimized design of conformational relationships.

**Fig. 2:**
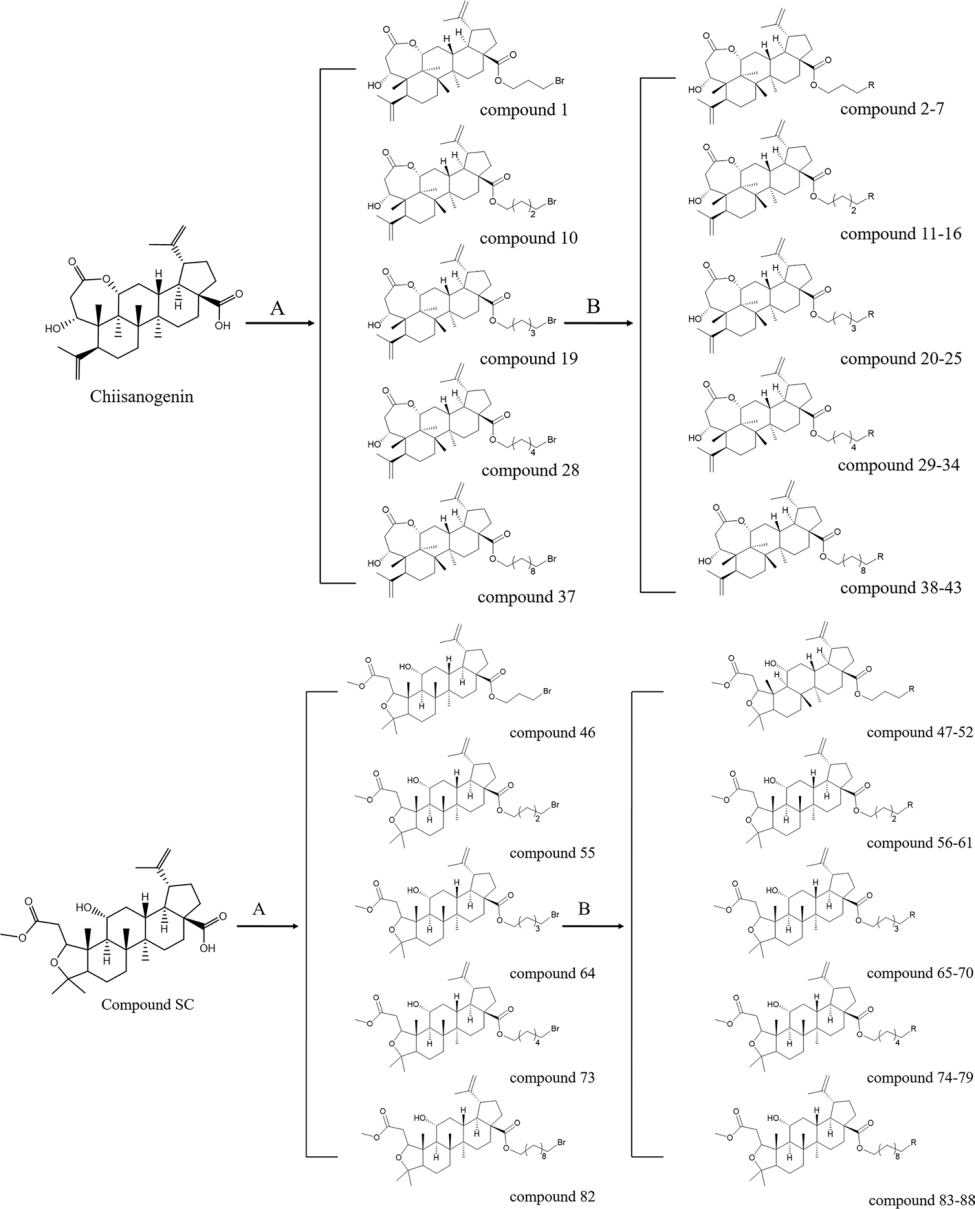
(**A**) AC, K_2_CO_3_, reflux, 24 h (**B**) ACN, K_2_CO_3_, reflux, 24 h, where R is selected from imidazole/2-methylimidazole/2-ethylimidazole/4-methylimidazole/1,2,4-triazole/tryptamine/5-methoxytryptamine.

**Fig. 3:**
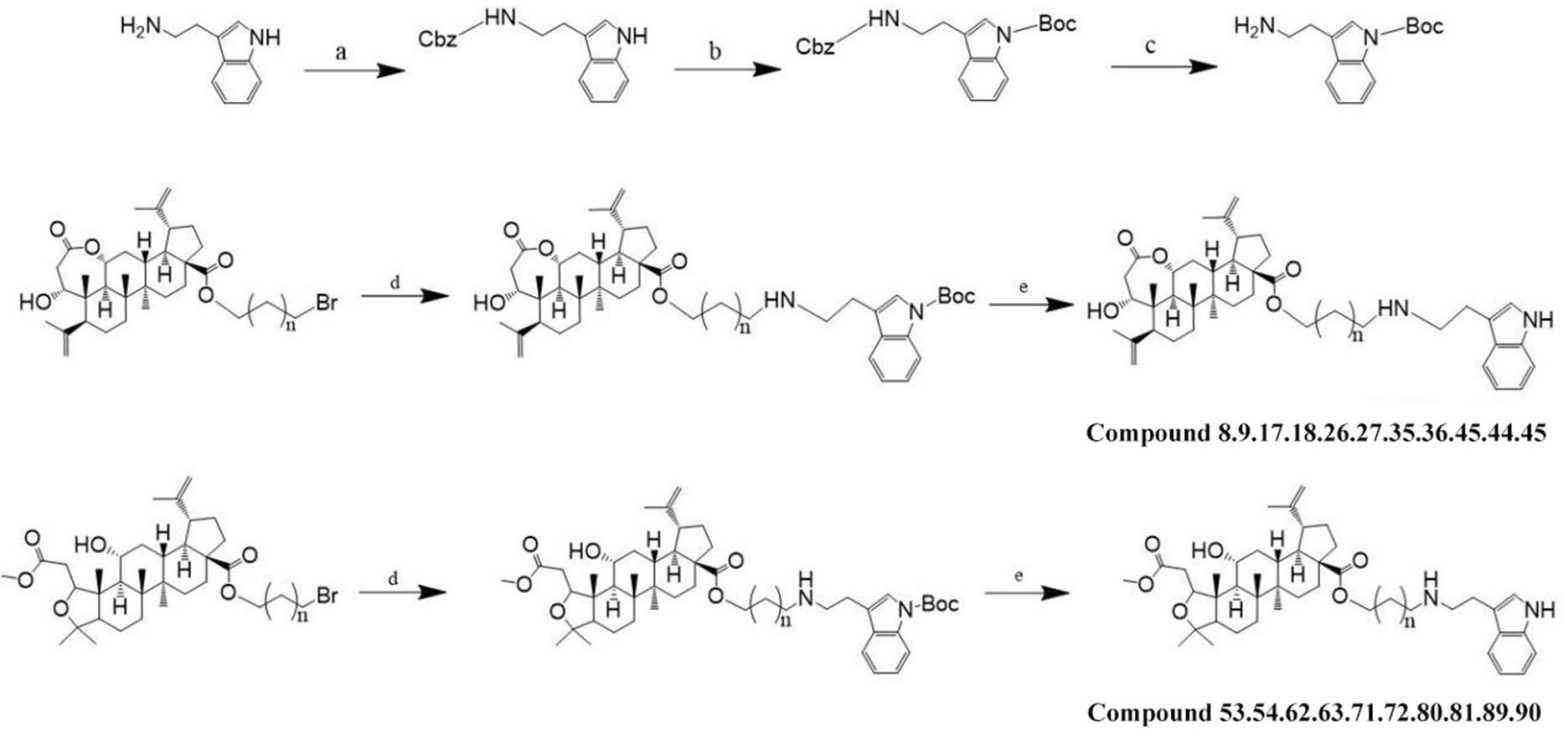
Synthesis of intermediate derivatives (in the case of tryptamine, for example, 5-methoxy tryptamine was also synthesized in this way). (**a**) Cbz-Cl, K_2_CO_3_, stir, 12 h. (**b**) Et_3_N, Boc_2_O, stir. (**c**) H_2_. (**d**) ACN, K_2_CO_3_, reflux, 24 h. (**e**) TCM: TFA (1:1), stir, 4 h.

Chiisanogenin and compound SC were used as the lead compounds. Their carboxyl groups were substituted with bromine in dibromoalkanes, and the two structures were linked together in the form of ester bonds to form intermediate compounds 1, 10, 19, 28, 37, 46, 55, 64, 73, 82. The terminal bromine in the intermediates was then reacted with selected molecules of indole, imidazole, and other fragments by substitution reaction with the iminyl hydrogen in the fragments to produce the target products 2–7, 11–16, 20–25, 29–34, 38–43, 47–52, 56–61, 65–70, 74- 79, 83–88, see Fig. 2. According to previous studies by the group, among the molecular fragments, the imino group of the tryptamine fragments, when attached to the intermediates, showed significant anti-hepatocellular carcinoma activity^23^. The tryptamine molecular fragment contain another active linkage site, the amino group, so this paper will further explore whether the activity will be further enhanced by linking the amino site in the tryptamine molecular fragment. To prevent the intermediate from connecting to the imino site, the boc group was preferentially attached to the imino group in the tryptamine molecular fragment for connection site protection. Then the de-Boc reaction was carried out again after determining that the amino site was connected to the intermediate (as shown in Fig. 3), which led to compounds 17–18, 26–27, 35–36, 44–45, 53–54, 62–63, 71–72, 80–81, 89–90.

### Toxicity studies of compound 27 on different human tumor cell lines

All the derivatives showed different degrees of cytotoxicity in various human-derived urological tumor cell lines. The specific IC_50_ values are shown in Supporting Information (Table S1). The table shows that all the derivatives were more sensitive to the bladder cancer cell line T24 in the screening of cytotoxic activity of the urological tumor cell lines. Therefore, the human-derived bladder cancer cell line T24 will be selected in the present paper for further studies. Meanwhile, further conformational relationship analysis revealed that the derivatives linking the tryptamine molecular fragments (compounds 7, 16, 25, 34, 43, 52, 61, 70, 79, and 88) had significant cytotoxic activity (IC_50_ = 3.23–5.82 μM) compared to the other molecular fragments, which coincided with the group’s previous activity studies in hepatocellular carcinoma. Surprisingly, after we replaced the attachment sites of the tryptamine molecular fragments in this paper (compounds 8, 9, 17, 18, 26, 27, 35, 36, 44, 45, 53, 54, 62, 63, 71, 72, 80, 81, 89, and 90), their cytotoxicity in urological tumor cell lines was again significantly enhanced (IC_50_ = 1.121–4.9 μM). Among them, the 5-methoxytryptamine derivatives (compounds 9, 18, 27, 36, 45, 54, 63, 72, 81 and 90) were highly tumor cytotoxic (IC_50_ = 1.121–3.89 μM) compared to the other derivatives as shown in Supporting Information (Table S1).

Interestingly, taking the 5-methoxytryptamine derivative with optimal activity as an example, the selection of the linking chain of the derivative and the analysis of the conformational relationship revealed that the derivative activity showed a tendency to increase and then decrease with the lengthening of the dibromoalkyl intermediate chain and reached the activity peak at C = 5 (IC_50_ = 1.121 μM), and Gemcitabine, a clinical first-line antitumor agent, was used as a control standard in the experiment with an IC_50_ of 6.352 ± 0.62 μM. Compound 27 had an IC_50_ value of 1.121 ± 0.25 μM as shown in Table 1. Therefore, we chose the investigated compound 27 acompound 27 hads a candidate compound and the T24 tumor cell line as a model to further validate its significant antitumor activity and mechanism of action studies.

**Table 1:** IC_50_ values of compounds on T24 cells.


Compound	X	R	IC_50_
Chiisanogenin	H	–	106.35 ± 4.36
9	(CH_2_)_3_		3.64 ± 0.45
18	(CH_2_)_4_		3.21 ± 0.32
27	(CH_2_)_5_		1.121 ± 0.25
36	(CH_2_)_6_		3.89 ± 0.45
45	(CH_2_)_10_		3.65 ± 0.45
20	(CH_2_)_5_		20.03 ± 2.19
21	(CH_2_)_5_		12.02 ± 1.23
22	(CH_2_)_5_		13.21 ± 1.28
23	(CH_2_)_5_		12.85 ± 1.89
24	(CH_2_)_5_		28.36 ± 1.56
25	(CH_2_)_5_		3.23 ± 0.89
26	(CH_2_)_5_		2.04 ± 0.66
27	(CH_2_)_5_		1.121 ± 0.25
GEM	–	–	6.352 ± 0.62

### Compound 27 significantly inhibited the growth of T24 cells

**Fig. 4:**
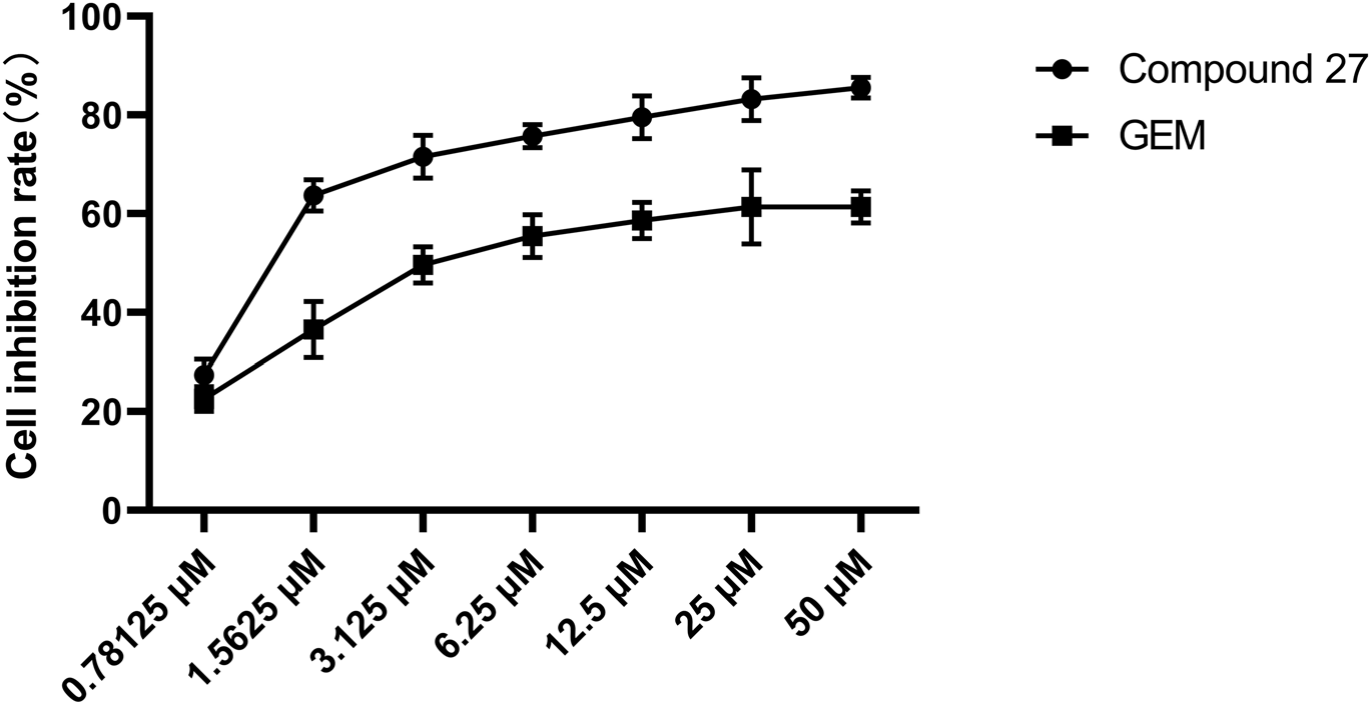
T24 growth curves at 48 h of treatment with different drugs. These values are expressed as mean ± standard deviation (n = 6).

In this experiment, we compared the antitumor activity of compound 27 with the existing clinical drug gemcitabine. The effect of compound 27 and gemcitabine on the proliferation of T24 cells was assessed by CCK8 assay. T24 human bladder cancer cells were treated with different concentrations (0.78125, 1.5625, 3.125, 6.25, 12.5, 25, 50) of compound 27 for 48 h. The results showed that compound 27 decreased the viability of T24 cells in a dose-dependent manner (Fig. 4). At a concentration of 0.78 μM, its cytotoxicity was significantly higher than that of gemcitabine, a first-line clinical antitumor drug. Meanwhile, at a lower concentration of 0.78125 μM, compound 27 inhibited T24 by 28.89%. This may make compound 27 an antitumor drug with development potential.

### Compound 27 significantly inhibited the proliferation of T24 cells

**Fig. 5:**
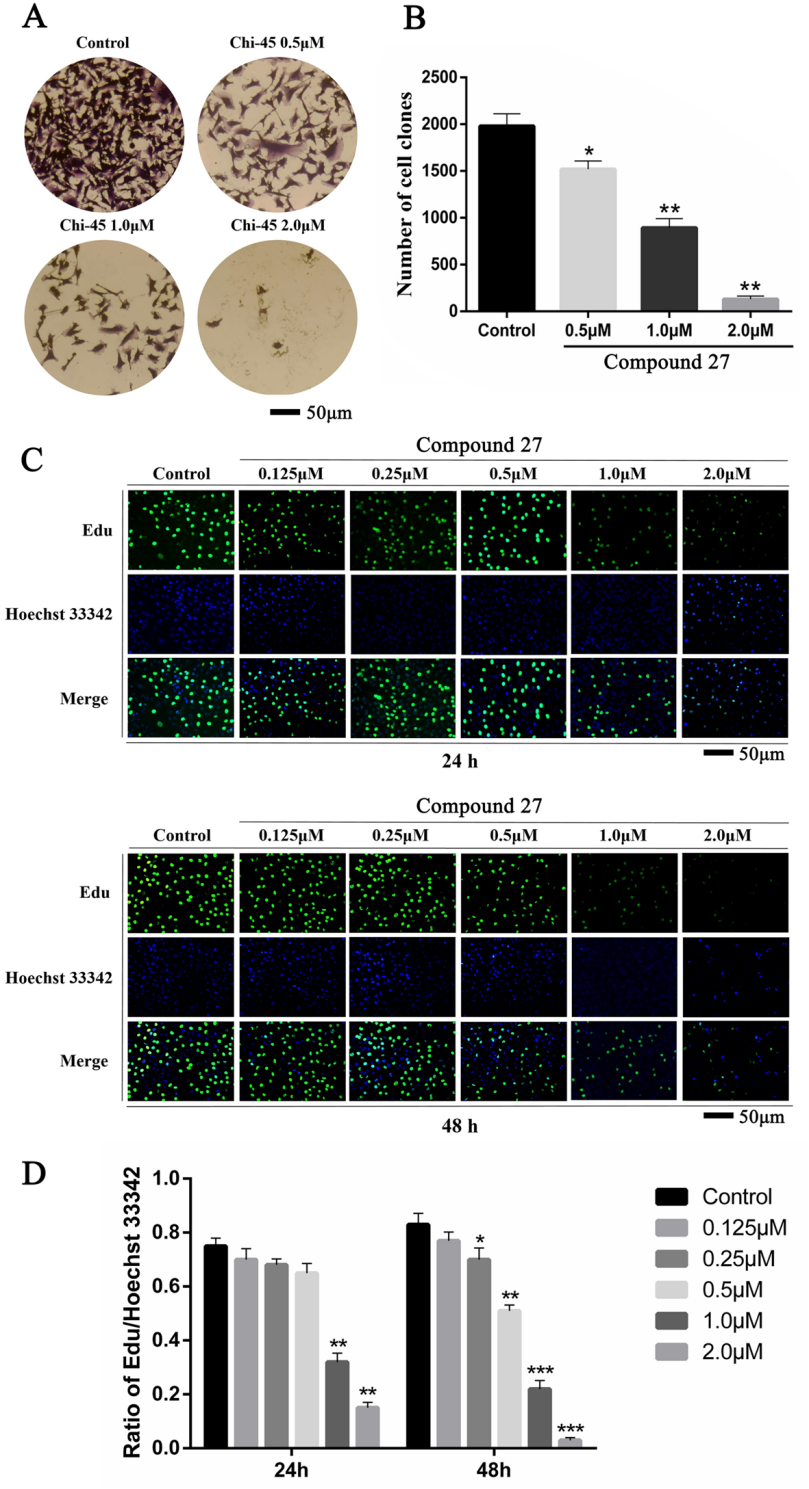
Effect of compound 27 on T24 cell proliferation. (**A**) Effect of compound 27 on clone formation of T24 cells (magnification × 100). (**B**) Histogram of the effect of compound 27 on clone formation of T24 cells. (**C**) Proliferative capacity of cells after 24 h and 48 h of action of different concentrations of compound 27 (magnification ×100). (**D**) Histograms show the percentage of proliferating cells and total cells. The values are expressed as mean ± standard deviation (n = 3). *p < 0.05, **p < 0.01, ***p < 0.001 compared to control.

The proliferation of tumor cells and their ability to migrate and invade surrounding tissues are major contributors to cancer-related death. This invasiveness is an important indicator for the study of tumor-associated signaling pathways and a key factor in assessing the efficacy of drug treatments and targeted therapies^25–27^. To deeply verify the inhibitory effect of compound 27 on the proliferation of T24 cells, EdU assay and cell clone formation assay were performed in this study, respectively. Plate clone formation assay was performed to determine the effect of compound 27 on the proliferative ability of individual cells, and the results were shown in Fig. 5A,B. At a high concentration of 2.0 μM, the proliferation of T24 cells was significantly inhibited (*p < 0.05, **p < 0.01). The inhibition of the proliferative ability of T24 cells by compound 27 was concentration-dependent. The results of EdU experiments showed that the proliferation of T24 cells was inhibited with the increase of the concentration and action time of compound 27. The weakening of the EdU fluorescence intensity indicated that the proliferative ability of T24 cells was weakened with the increase of the concentration of compound 27 (Fig. 5C,D). We explored the three concentrations of compound 27, respectively, 0.125, 0.25, and 0.5 μM, which had no significant effect on the proliferation of T24 cells at 24 h. When the time was extended to 48 h, almost all concentrations of the compounds had a significant inhibitory effect on the proliferation of T24 cells (*p < 0.05, **p < 0.01, ***p < 0.001). Therefore, three concentrations of compound 27, 0.125 μM, 0.25 μM and 0.50 μM, and an exposure time of 24 h were selected to study migration and invasion of T24 cells.

### Compound 27 significantly inhibits the migration and invasion of T24 cells

**Fig. 6:**
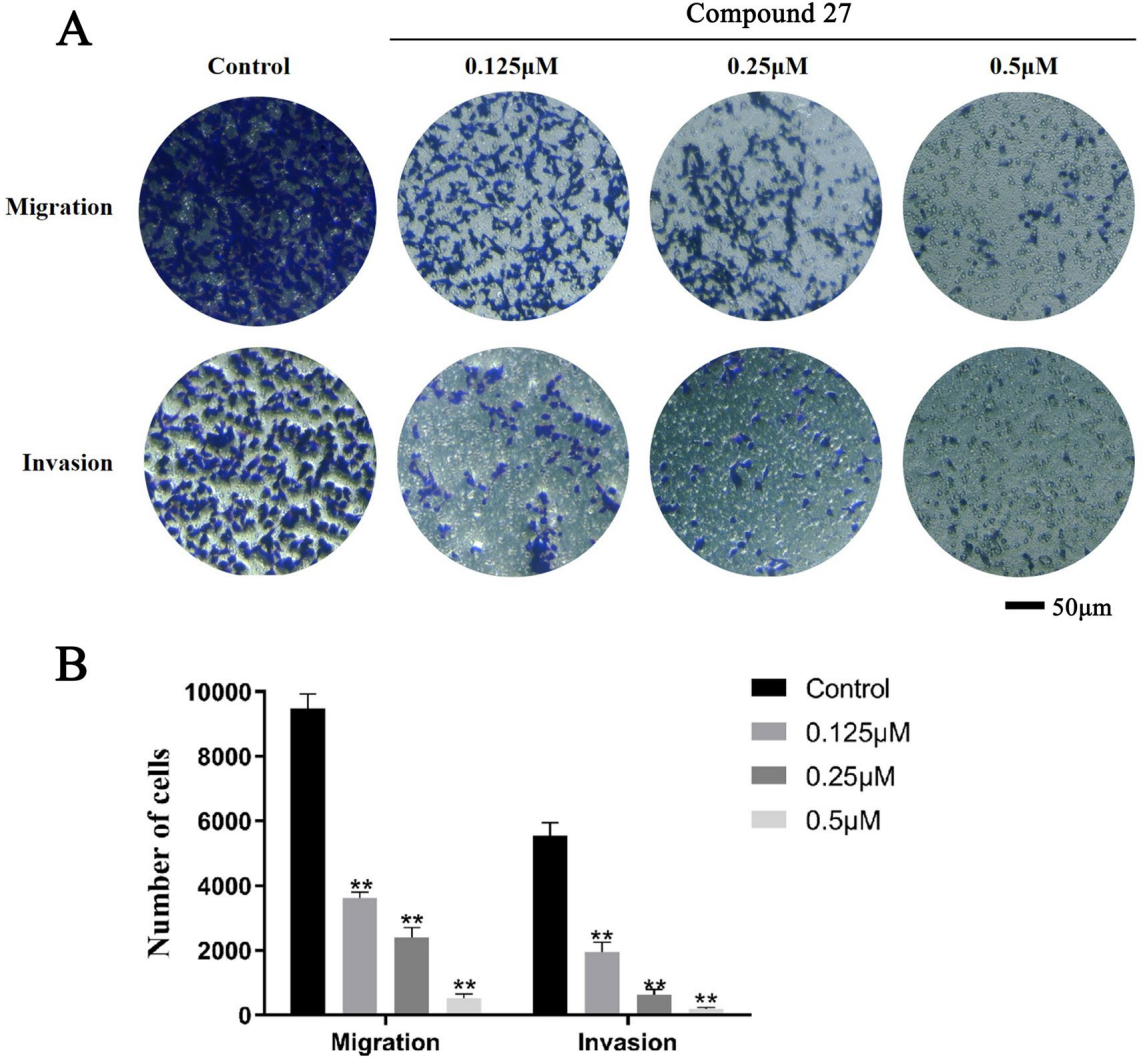
Effect of compound 27 on migration and invasion of T24 cells. (**A**) After different concentrations of compound 27 acted on T24 cells for 24 h, the cells in the lower layer of Transwell were stained with a crystal violet staining solution (magnification × 100). (**B**) Histogram showing the number of migrated/invaded cells. Values are expressed as mean ± SD (n = 3). *p < 0.05, **p < 0.01 compared with control.

In this experiment, we evaluated the effects of different concentrations of compound 27 on the migration and invasion abilities of T24 cells by Transwell assays. The results are shown in Fig. 6A and B. Compound 27, which exhibited a concentration-dependent effect on the migration and invasion of T24 cells. At concentrations of 0.125, 0.25, and 0.5 μM, compared with the control group, the migration ability of T24 cells was significantly decreased (*p < 0.05, **p < 0.01), and the invasion ability was also significantly inhibited. These results indicate that compound 27 can effectively inhibit the migration and invasion of T24 cells.

### Compound 27 suppresses T24 cell proliferation by regulating cell cycle and inducing cell death

**Fig. 7:**
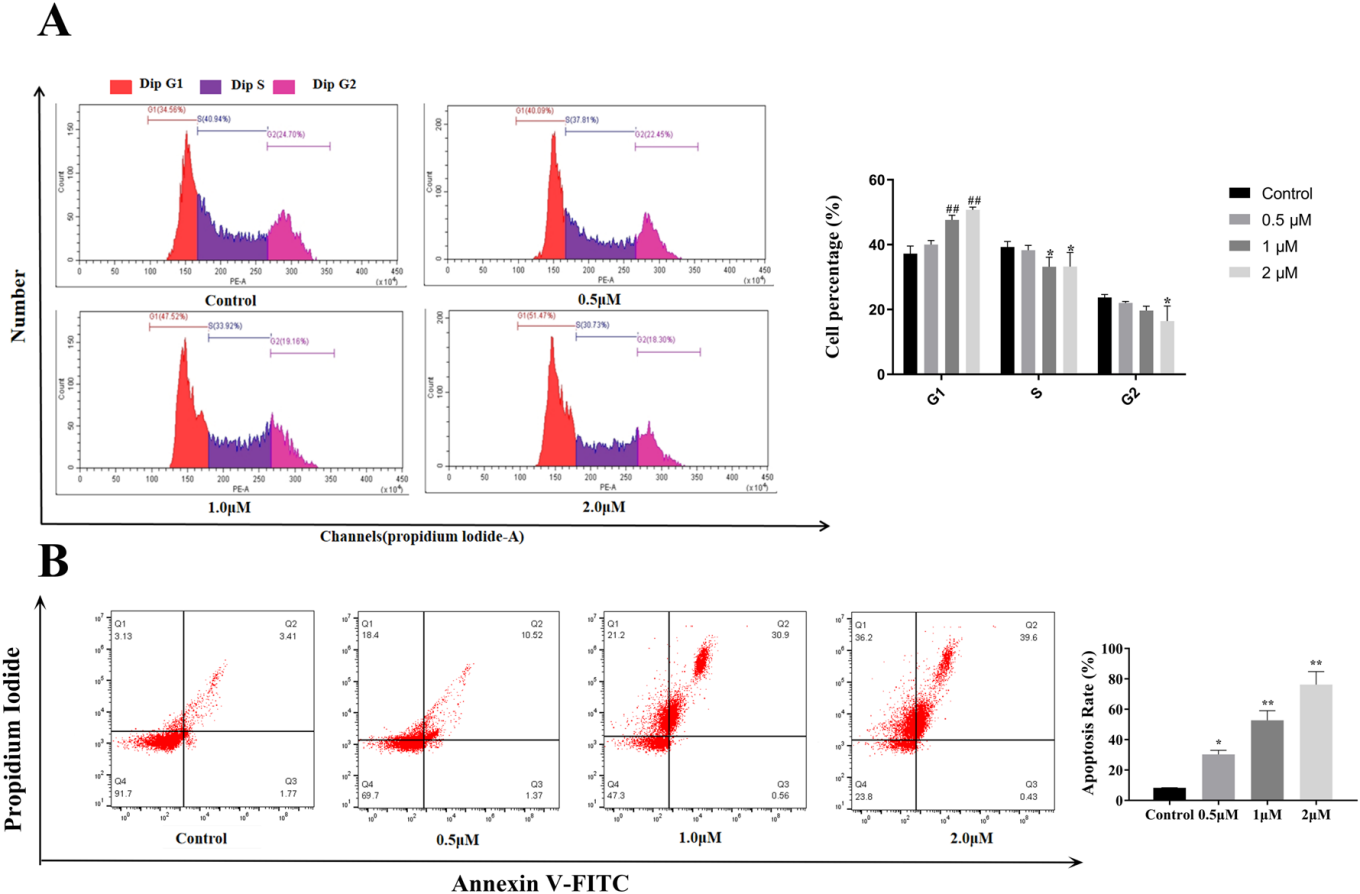
Effect of compound 27 on T24 cell cycle and apoptosis. (**A**) T24 cells were tested by flow cytometry to investigate the effect of compound 27 on the cell cycle. The cell cycle level and occupancy of T24 cells at different cycle times were measured at multiple time points, and the results were presented in the form of histograms. Values are expressed as mean ± SD (n = 3), *p < 0.05, **p < 0.01, ***p < 0.001 compared with control (decreased); ^#^*p* < 0.05 and ^##^*p* < 0.01 compared with control (increased). (**B**) Effect of compound 27 on apoptosis of T24 cells. Values are expressed as mean ± standard deviation (n = 3). Compared to control group, *p < 0.05, **p < 0.01.

The cell cycle analysis results showed (Fig. 7A; *p < 0.05 and **p < 0.01 vs. the control group (decreased); ^#^*p* < 0.05 and ^##^*p* < 0.01 vs. the control group (elevated)) that compound 27 treatment led to a significant increase in the proportion of T24 cells in the G1 phase, accompanied by a decrease in the proportions of cells in the S and G2 phases, indicating that compound 27 could induce G1 phase arrest and inhibit the G1/S phase transition in T24 cells. These results confirm that compound 27 affects the cell cycle process, exerting its anti-tumor effect.

Annexin V has the property of binding readily to phosphatidylserine, for which it has a high affinity. Fluorescein FITC labelled Annexin V was used as a fluorescent probe to detect the onset of early and late apoptosis. Propidium iodide (PI) dye is unable to stain living cells, whereas necrotic cells can be stained by PI dye. Therefore, using Annexin V-FITC with PI double staining, early and late apoptosis as well as necrotic cells can be distinguished. The effect of compound 27 on apoptosis in T24 cells is shown in Fig. 7B. The apoptosis of T24 cells at low (0.5 μM) and medium (1.0 μM) concentrations was dominated by early apoptosis, and at high (2.0 μM) concentrations, the apoptosis was dominated by late apoptosis with a small amount of early apoptosis and necrosis, and the overall apoptosis rate of the cells was close to 76.2% at a concentration of 2.0 μM. Compound 27 inhibits the growth of T24 cells by inhibiting cell proliferation and promoting cell death.

### Compound 27 inhibits the growth of bladder cancer in vivo

**Fig. 8:**
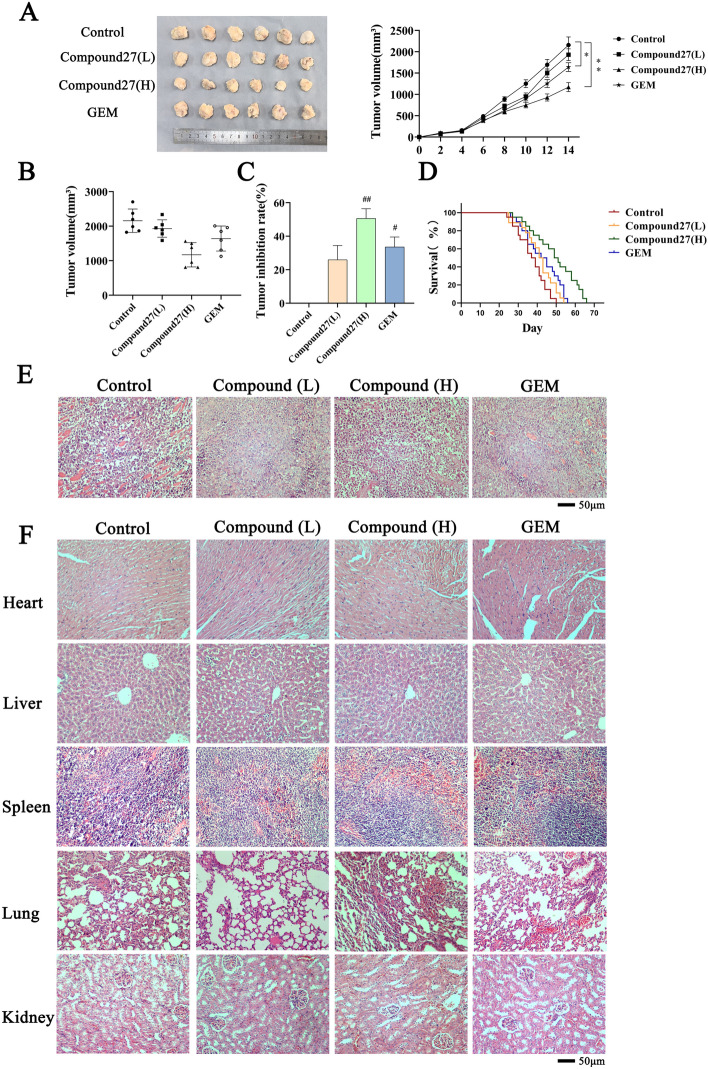
In vivo antitumor effect of compound 27. (**A**) Changes in tumor size in loaded mice. (**B**) Tumor growth curves in loaded mice. (**C**) Tumor inhibition rate. (**D**) Survival curves. (**E**) H&E staining of tumor tissues It is used to observe the morphology and structure of tumor cells, and to analyze whether compound 27 causes changes such as necrosis and apoptosis of tumor cells (magnification ×100). (**F**) H&E staining of tumor tissues and organs To evaluate the potential toxicity and side effects of compound 27 on normal tissues and organs, ensuring anti-tumor and reducing normal tissue damage (magnification ×100).The numerical values are expressed as mean ± standard deviation (n = 6) and compared with the control group, *p < 0.05, **p < 0.01, ^#^*p* < 0.05, ^##^*p* < 0.01.

To evaluate the inhibitory effect of compound 27 on bladder cancer in vivo, we established a subcutaneous tumor xenograft model of bladder cancer in nude mice using T24 cells. As shown in Fig. 8A–C, compared with the blank control group (^#^*p* < 0.05 and ^##^*p* < 0.01), the tumor volume in the high-dose group of compound 27 was significantly reduced. This indicates that compound 27 has a practical anti-tumor effect in vivo. In addition, the tumor inhibition rates in the positive drug, low-dose, and high-dose groups were 33.56%, 25.94%, and 50.65%, respectively. These results together indicate that compound 27 has a significant tumor growth inhibitory ability in treating bladder cancer. Survival curve analysis was performed using GraphPad Prism 8. The results are shown in Fig. 8D. The median survival times of the mice were 50 days for the saline group, 56 days for the gemcitabine (GEM) group, 66 days for the high-dose compound 27 group, and 54 days for the low-dose compound 27 group. It can be seen that the median survival time of mice in the control group was the shortest among all the administered groups. The median survival time was significantly longer in the high-dose group compared to the positive group. In conclusion, it is promising that Compound 27 can substantially inhibit tumor growth and prolong the median survival time of mice.

### Histopathological analysis

To further study the effect of compound 27 in vivo, the tumor tissues and mouse organs collected from the xenograft model were stained with H&E. As shown in Fig. 8E and F, the subcutaneous tumors in the blank control group exhibited the characteristic of enlarged tumor cell nuclei. Compared with the control group, the apoptosis of T24 cells in the compound 27 treatment group was significantly increased, and different degrees of cell necrosis were observed in the positive drug group, low-dose group, and high-dose group, indicating that compound 27 has significant anti-bladder cancer activity. The H&E-stained sections of the mouse organs were observed under a microscope. Compared with the blank control group, no significant histopathological changes were observed in all the organs in the compound 27 group. This indicates that compound 27 has no significant toxic or side effects on BALB/c nude mice.

### RNA sequencing data analysis

**Fig. 9:**
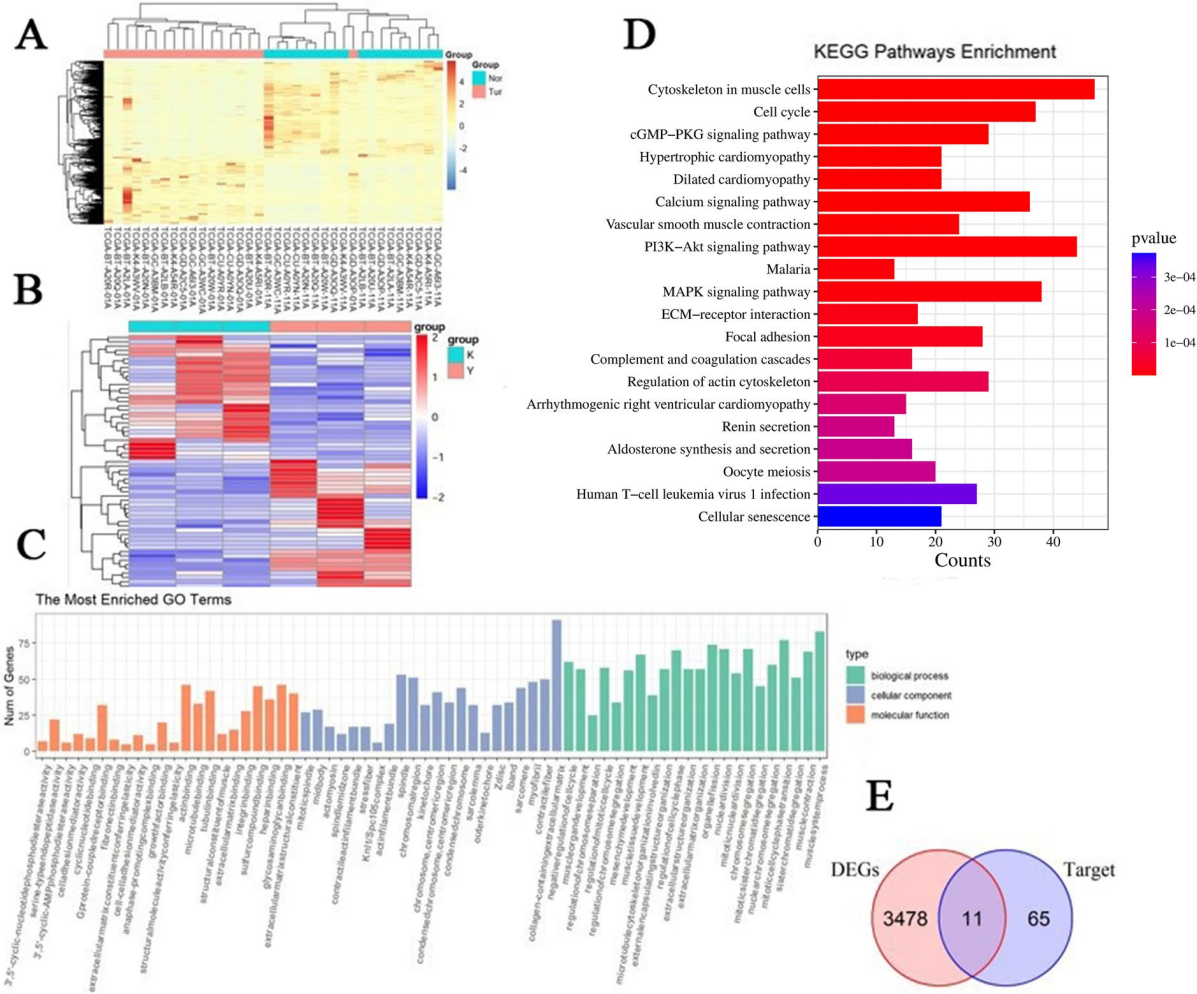
RNA sequencing data analysis results. (**A**) Heatmap of differentially expressed genes in TCGA dataset. (**B**) Heatmap of the expression patterns of all DEGs. Horizontal coordinates in the graph indicate sample names and clustering results of samples, and vertical coordinates indicate differential genes and clustering results of genes. Colors represent gene expression in sample level log2 (FPKM + 1). (**C**) GO enrichment analysis. (**D**) Enrichment histogram of DEGs in KEGG pathway. The horizontal coordinate is GeneNum, the number of relevant genes annotated in the entry, and the vertical coordinate is for each pathway entry. The color of the histogram represents the p-value of the hypergeometric test. (**E**) Venn diagram demonstrates the intersections of genes between DEGs data and Targets data.

This study aims to elucidate the molecular mechanisms underlying bladder cancer development and identify potential therapeutic targets. We obtained RNA sequencing data from the TCGA-BLCA (bladder urothelial carcinoma) cohort via the TCGA database (https://xenabrowser.net). Following rigorous quality control and preprocessing, we included 18 paired samples, which comprised primary bladder cancer tissues and corresponding adjacent normal tissues. Differential expression analysis was performed using the DESeq2 package, which employs a negative binomial distribution model and is known for its robust handling of biological replicates and outliers, facilitating accurate identification of differentially expressed genes (DEGs) (Fig. 9A). To ensure the reliability of the results, we applied conventional filtering criteria: adjusted p-value < 0.05 and |log2FoldChange|> 1, to identify DEGs between tumor and normal tissues. Subsequently, we conducted functional annotation of the identified DEGs using clusterProfiler, including Gene Ontology (GO) enrichment analysis and Kyoto Encyclopedia of Genes and Genomes (KEGG) pathway analysis^28–30^ (Fig. 9C and D). Parallel differential expression analysis was performed between drug-treated and tumor groups to identify drug-related target genes (Targets) (Fig. 9B). By comparing the DEGs and drug target genes (Targets), we identified 11 intersecting genes and generated a Venn diagram (Fig. 9E). These genes exhibit significant expression changes during tumorigenesis and in response to therapeutic intervention, suggesting their potential as therapeutic targets or prognostic markers.

### Compound 27 administration correlation analysis

**Fig. 10:**
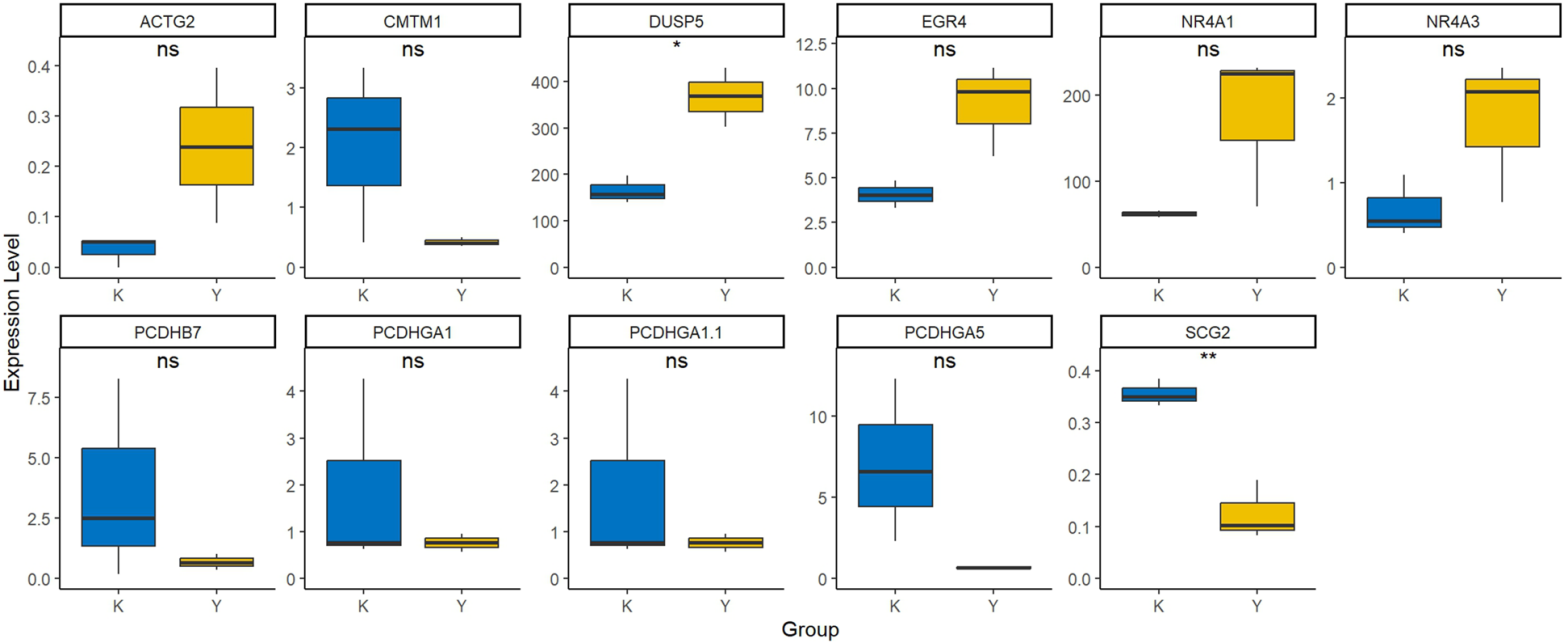
Compound 27 administration correlation analysis (*p < 0.05, **p < 0.01).

To investigate the impact of compound 27 on bladder cancer gene expression, As shown in Fig. 10, we conducted a systematic comparative analysis of 11 differentially expressed genes (*ACTG2*, *CMTM1*, *DNER*, *EGBA*, *NRAP1*, *NRAP3*, *PCD* + *B7*, *PCDHA1*, *PCDHA11*, *PCDHA6*, and *SCG2*). By comparing gene expression levels between the experimental group (Y) and the control group (K), we visualized the expression differences of each gene using box plots and assessed their statistical significance using t-tests (*p < 0.05, **p < 0.01). The results indicated that only *DUSP5* and *SCG2* exhibited significant differences among all differentially expressed genes, with *DUSP5* being upregulated in the experimental group and *SCG2* being downregulated. This finding suggests that compound 27 may exert its anti-bladder cancer therapeutic effect by upregulating *DUSP5* and downregulating *SCG2*, which provides important clues for further elucidating the mechanism of action of this compound.

### Clinical correlation analysis

The staging of bladder cancer depends on the extent of tumor invasion, the involvement of lymph nodes, and the presence of distant metastasis. The higher the stage, the poorer the prognosis for the patient is generally^31,32^. The incidence of bladder cancer is higher in men than in women, with a ratio of about 3:1. Gender may also be a factor affecting treatment outcomes and prognosis. Studies have found that there may be differences in the biological characteristics of bladder cancer and responses to treatment between men and women^33^. Lymph node metastasis and distant metastasis are adverse factors affecting the prognosis of bladder cancer. The presence of lymph node metastasis usually indicates that the disease has advanced to a later stage, making treatment more complex^34^. Therefore, clinical correlation analysis was conducted on *DUSP5* and *SCG2*.

**Fig. 11:**
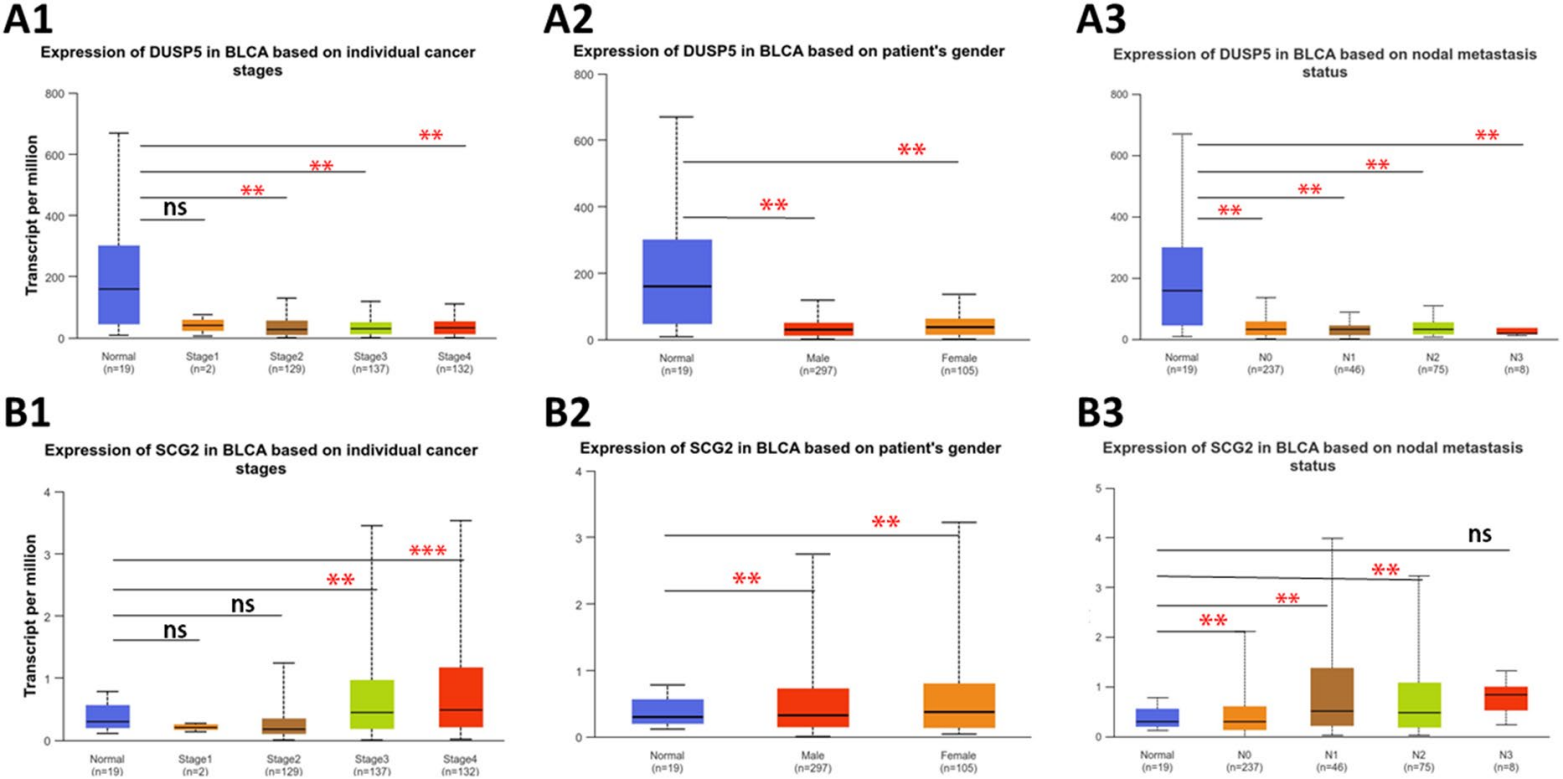
Clinical correlation analysis results for key genes (A1-B1 patient gender, A2-B2 individual cancer staging, A3-B3 lymph node metastasis status. (ns > 0.05, *p < 0.05, **p < 0.01, ***p < 0.001).

We used the UALCAN database to analyze the expression of *DUSP5* and *SCG2* genes in different subtypes of bladder cancer patients (gender, cancer stage, and lymph node metastasis) was analyzed (Fig. 11). The results indicated that the expression level of *DUSP5* in the standard group was significantly lower than in the tumor group for patient gender, cancer stages 2, 3, and 4, and lymph node metastasis status. At the same time, it was not significant in stage 1. For *SCG2*, the expression level in the standard group was significantly higher than that in the tumor group for patient gender, cancer stages 3 and 4, and lymph node metastasis status. At the same time, it was insignificant in stages 1 and 2. *DUSP5* and *SCG2* are significantly correlated with the clinical staging, gender, and lymph node metastasis of bladder cancer.

### Survival analysis

Depending on whether bladder cancer is non-muscle-invasive or muscle-invasive, bladder cancer exhibits different molecular subtypes and multiple pathogenic pathways, and early detection usually indicates a better prognosis^35^.

**Fig. 12:**
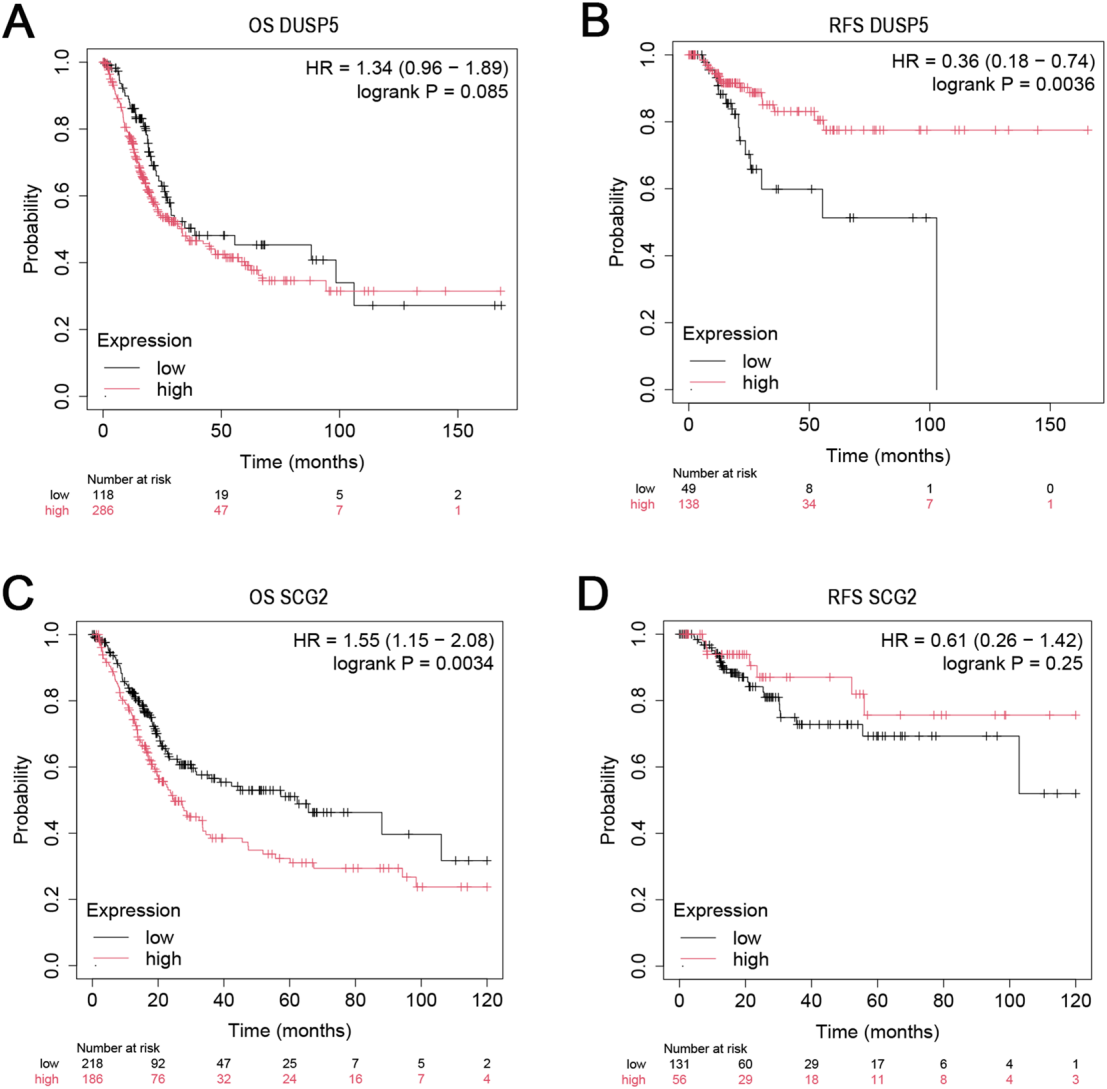
Decade prognosis results for key genes. (**A**) *DUSP5* overall survival; (**B**) *DUSP5* relapse-free survival; (**C**) *SCG2* overall survival; (**D**) *SCG2* relapse-free survival.

The Kaplan–Meier plotter database was used to predict the 10-year prognostic values for *DUSP5* and *SCG2*, with the results shown in Fig. 12. The vertical axis represents the survival rate, and the horizontal axis represents the survival time. The starting point is the follow-up time; the curve’s descent indicates patient death and the " + " signs represent censoring (the last follow-up time for patients still alive). The p-value is from the log-rank test, which measures whether there is a significant difference in survival rates between the plotted curves. HR > 1 indicates that the subject of study is a risk factor; HR < 1 indicates that the subject of study is a protective factor; HR = 1 suggests that the subject of study does not affect survival time. Both OS and RFS are important indicators for assessing the effectiveness of bladder cancer treatments, providing complementary information.

The results show that after the log-rank test, the p-value for *SCG2*'s OS is less than 0.05 and has significant survival implications within 5 years (with an HR value of 1.55). The p-value for *DUSP5*'s overall survival is greater than 0.05 and is not statistically significant. *DUSP5*'s p-value in RFS is less than 0.05, indicating significant statistical significance (with HR values of 0.36). *SCG2*'s p-value in RFS is greater than 0.05 and is not statistically significant. In actual `clinical research and practice, the importance of OS and RFS depends on the study’s purpose and the treatment’s goals. When assessing the effectiveness of radical treatments (such as radical cystectomy), OS may be the primary endpoint because it directly reflects the impact of treatment on survival^36^. When evaluating the effects of bladder-preserving treatments or adjuvant therapies, RFS may be an important secondary endpoint, as it can help understand the treatment’s effect on controlling local disease and delaying recurrence^37^. Therefore, both *DUSP5* and *SCG2* have prognostic value for survival outcomes.

### Immune infiltration analysis

**Fig. 13:**
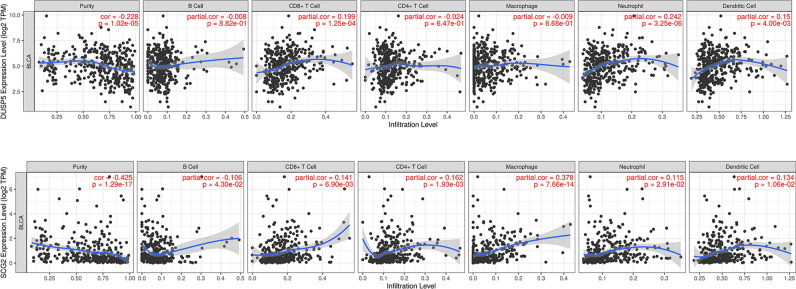
Immune Infiltration Results (**A**) The correlation of *DUSP5* with tumor purity in six types of infiltrating immune cells. (**B**) The correlation of *SCG2* with tumor purity in six types of infiltrating immune cells.

The tumor microenvironment refers to the non-cancer cell environment composed of immune cells surrounding the tumor cells. We used the TIMER database to analyze the relationship between the expression levels of the two genes, *DUSP5* and *SCG2*, and the purity of bladder cancer, as well as the correlation with six types of infiltrating immune cells (B cells, CD4^+^ T cells, CD8^+^ T cells, neutrophils, macrophages, and dendritic cells) in patients with bladder cancer. The results are shown in Fig. 13. The results indicate that the expression of *DUSP5* is significantly negatively correlated with tumor purity and positively correlated with the immune infiltration of CD8^+^ T cells, neutrophils, and dendritic cells. The expression level of *SCG2* is significantly negatively correlated with tumor purity and significantly positively correlated with the immune infiltration of CD4^+^ T cells, CD8^+^ T cells, and macrophages.

Studies suggest that *DUSP5* may influence the immune microenvironment of bladder cancer by potentially affecting the activity of T cells and natural killer (NK) cells^38^. An increase in *DUSP5* expression may be associated with tumor immune evasion. High expression of *DUSP5* may promote the survival and proliferation of tumor cells by suppressing immune responses^39^. Research has found that *SCG2* may regulate the invasiveness and metastatic properties of tumor cells, which could be closely related to the infiltration and function of immune cells within the tumor^40^. Furthermore, the expression level of *SCG2* may be closely associated with the response of bladder cancer patients to immunotherapy, potentially serving as a biomarker for predicting the efficacy of immunological treatments^41^. The downregulation of *SCG2* may have a significant impact on the immune response to tumors by reducing the infiltration of immune-suppressive cells, downregulating the expression of immune checkpoint molecules, and altering the cytokine secretion in the immune microenvironment^42,43^. These changes may enhance the immune system’s ability to recognize and attack tumor cells, thereby providing more favorable conditions for immunotherapy.

**Fig. 14:**
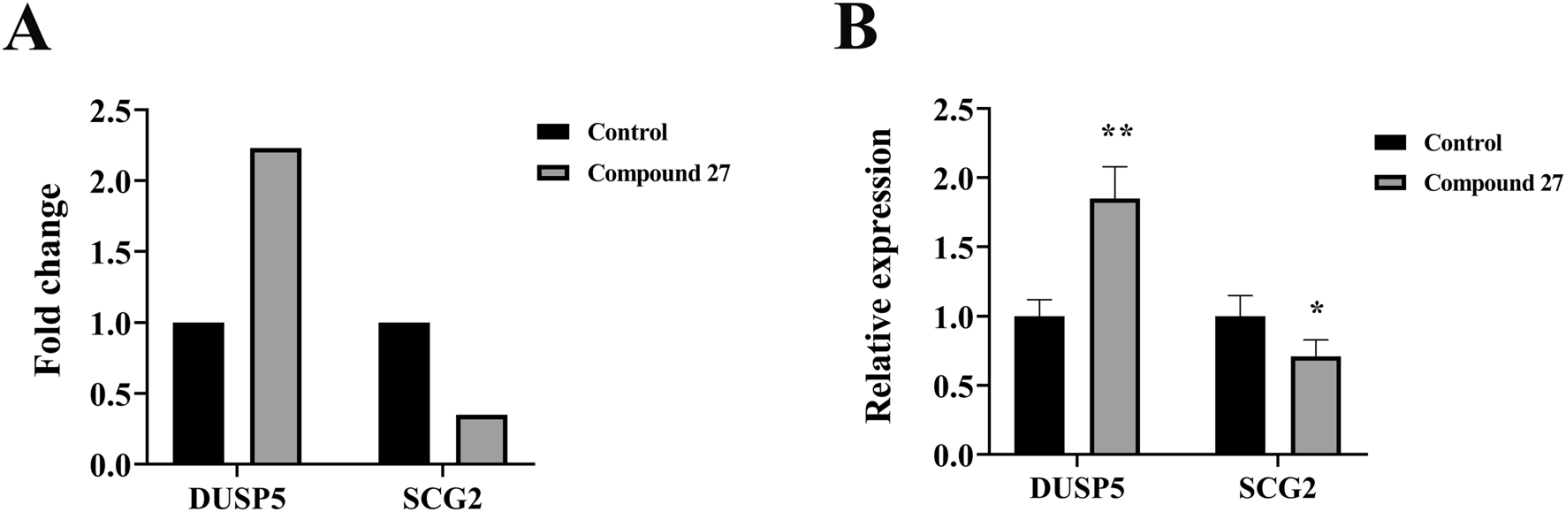
Expression and validation of *DUSP5* and *SCG2* and core genes. (**A**) Expression trend of core genes in the transcriptome. (**B**) Relative mRNA expression levels of core genes, *p < 0.05 and **p < 0.01 compared to control.

In this study, we explored the effect on the gene expression of *DUSP5* and *SCG2* in tumor cells before and after treatment. As shown in Fig. 14A, the results of RNA-seq analysis clearly revealed that the expression of *DUSP5* and *SCG2* showed significant differences in the treatment and control groups. To ensure the reliability and accuracy of the transcriptome sequencing results, the same samples were further examined using RT-qPCR technology, and the relevant results are presented in Fig. 14B. The gene expression trends presented by RT-qPCR assay were highly consistent with the RNA-seq data, and this validation result provided strong support for the accuracy of the transcriptome data. By comparing the fold change and relative expression data, it was found that the expression of *DUSP5* was significantly upregulated in the treatment group compared to the control group. This result implies that the treatment may have a potential impact on immune cell activity and tumor dynamics by upregulating the expression of *DUSP5*. Increased expression of *DUSP5* may play a key role in immune regulation and remodelling of the tumor microenvironment. Meanwhile, *SCG2* expression was also significantly altered in the treatment group. Compared with the control group, the level of *SCG2* was significantly reduced in the treatment group. This down-regulation may be important in the regulation of immune response and tumor behaviour. Lower *SCG2* levels may help to enhance the body’s immune recognition and attack ability against tumor cells, thus playing a positive role in the tumor treatment process. In summary, the present study further clarified the effects of compound 27 on the expression of *DUSP5* and *SCG2* in tumor cells after treatment by a combination of RNA-seq and RT-qPCR.

### Molecular docking and kinetic simulation analysis of compound 27 with *DUSP5* protein

**Fig. 15:**
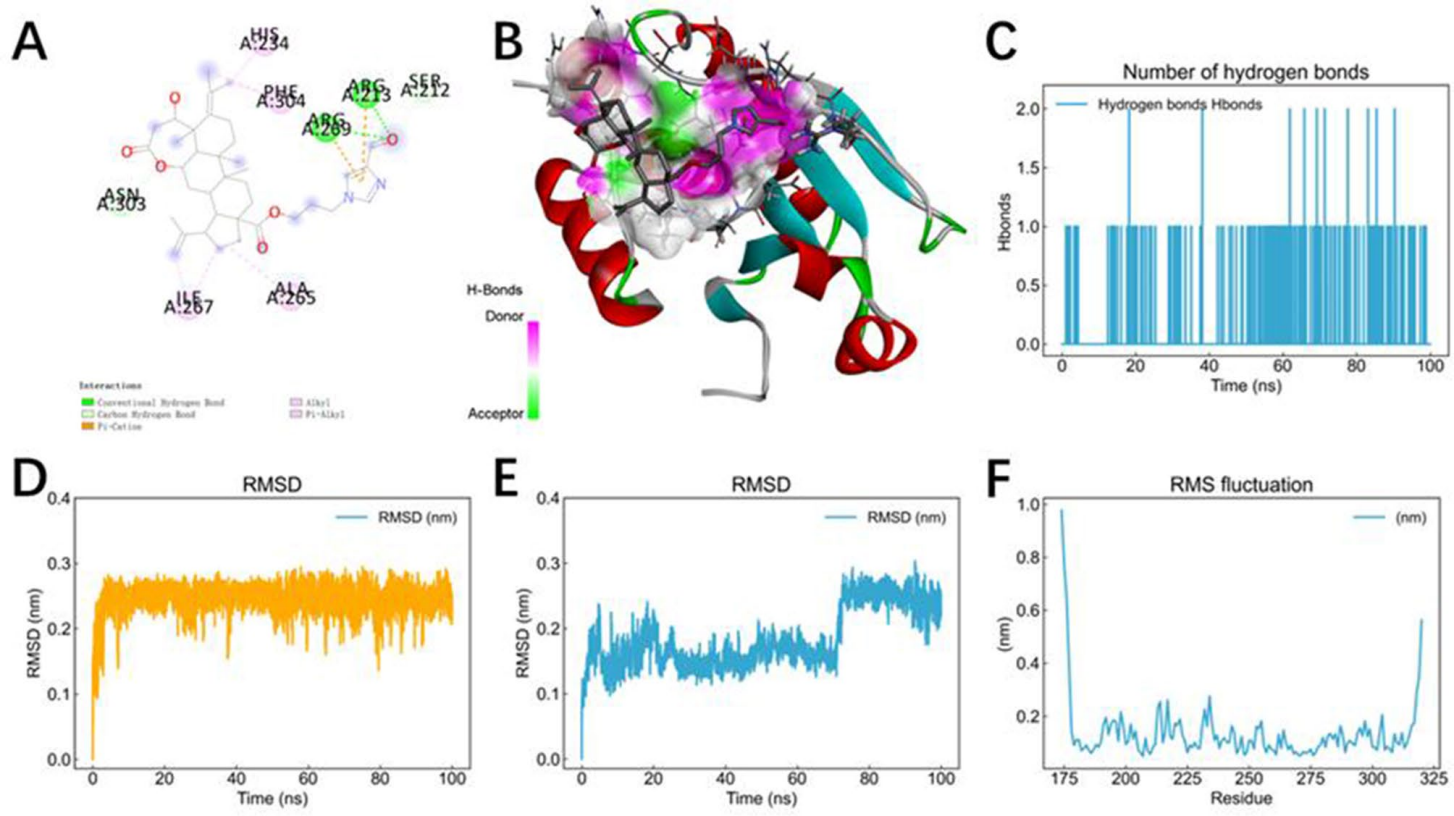
Molecular docking and kinetic simulation analysis of compound 27 with *DUSP5* protein. (**A**) Planar binding conformation of compound 27 with *DUSP5* protein. (**B**) Hydrogen bonding and hydrophobic interactions between compound 27 and the structural domain of *DUSP5* protein. (**C**) The number of hydrogen bonds. (**D**) Changes in protein RMSDs. (**E**) Changes in ligand RMSD. (**F**) RMSF of protein alpha-C.

Protein docking was performed using Autodock Vina. The docking score of *DUSP5* with compound 27 was − 6.3 kcal/mol, less than − 5 kcal/mol. It is generally believed that a binding score less than − 5 kcal/mol indicates a strong binding capacity between the ligand and the receptor, which may have potential biological activity. This score represents the free energy of binding between the ligand and the receptor. The more negative the value, the more stable the binding. Therefore, the binding between *DUSP5* and compound 27 is stable. The docking conformation was analyzed using Discovery Studio Visualizer 2020. Figure 15A shows the interactions of compound 27 with multiple amino acid residues of the *DUSP5* protein. Figure 15B shows the three-dimensional structure of the *DUSP5* protein, including α-helices (red), β-sheets (green), and loop structures (gray). Compound 27 is embedded in the protein’s binding pocket and is surrounded by the protein structure. The binding of compound 27 with *DUSP5* involves various non-covalent interactions, including hydrogen bonds, π-π, π-alkyl, and hydrophobic interactions. These interactions work together to promote the high complementarity and specific binding of compound 27 with the binding pocket of *DUSP5*. This analysis further confirms that compound 27 has a superior spatial conformation due to its appropriate molecular weight and spatial distribution. Therefore, it can bind well to the active pocket of *DUSP5*.

The results of the molecular dynamics analysis were visualized, and the results were subsequently analyzed. The RSMD curve in the figure is an indicator of the stability of the protein–ligand complex, and a smoother RMSD curve represents a more stable complex. The RMSF curve represents the degree of fluctuation of amino acid residues during kinetic simulation, and a lower RMSF value represents less fluctuating amino acid residue and a stable structure. The curve of the change in the number of hydrogen bonds represents the fluctuation in the number of hydrogen bonds formed by the protein–ligand during the simulation process. The more hydrogen bonds there are, the more stable the binding is. As shown in Fig. 15C, the relatively large number of hydrogen bonds indicates that Compound 27 is stably bound to *DUSP5*. As shown in Fig. 15D and E, the protein structure reaches equilibrium after 10 ns RMSD during the simulation. The RMSD of the small molecule reaches equilibrium after 70 ns, and the small molecule undergoes significant motion during the simulation. As shown in Fig. 15F, the RMSF is less than 1 Å, indicating that the residues have minimal fluctuation relative to their average position, and the structure is more rigid and less fluctuating. In summary, it can be seen that the structure of the protein is very stable during the simulation.

## Discussion

Bladder cancer, which is also referred to as urothelial carcinoma, is prevalent with significant morbidity and mortality rates^44,45^. The disease presents distinct molecular subtypes and diverse pathogenic pathways based on whether it is non-muscle-invasive or muscle-invasive, with early detection often leading to more favorable outcomes^46^. It is important to find potential new methods, targets, and effective therapeutic drugs for bladder cancer treatment^47^, and to identify key targets and effective therapeutic drugs for bladder cancer. In recent years, there has been a surge in interest among researchers worldwide regarding the anticancer properties of triterpenoids^48^. This study initially conducted a comprehensive cell activity screen of 90 chiisanoside derivatives synthesized in-house, followed by an in-depth investigation into their anticancer mechanisms. Cytotoxicity experiments revealed that compound 27 had the most pronounced inhibitory effect on T24 bladder cancer cells, with an inhibition rate nearing 84.01%. These findings suggest compound 27 possesses substantial anticancer potential, offering a novel therapeutic option for bladder cancer treatment.

The capacity of tumor cells to proliferate and their ability to migrate and invade surrounding tissues are primary drivers of cancer-related mortality. This invasiveness is a critical biomarker for studying tumor-associated signaling pathways and a key determinant in evaluating the effectiveness of drugs and targeted therapies^25–27^. Moreover, invasiveness is also significant in oncogene research. In this study, the impact of compound 27 was assessed through EdU assay, cell cloning assay, and Transwell migration and invasion assays. EdU assay results indicated that the proliferation of T24 cells was markedly suppressed with increasing concentrations of compound 27 and extended treatment durations. Cell clone formation, Transwell migration, and invasion assays demonstrated that compound 27’s inhibition of T24 cell proliferation was concentration-dependent. In conclusion, compound 27 significantly hindered the proliferation, migration, and invasion of T24 cells, exhibiting promising antitumor activity.

To explore the molecular mechanisms of compound 27 in bladder cancer and find therapeutic targets, we analyzed transcriptomic data to find genes and pathways affected by the compound, understanding its role in promoting apoptosis and inhibiting tumor growth. After drug treatment, we identified 11 common targets by comparing differentially expressed genes with the TCGA database. Drug administration correlation analysis revealed that compound 27 significantly upregulated the expression of *DUSP5* and downregulated the expression of SCG5. Clinical, survival, and immune infiltration analyses confirmed their importance. Molecular docking and dynamics simulations showed good binding between *DUSP5* and compound 27. KEGG pathway enrichment analysis showed that *DUSP5* is primarily regulated through the MAPK signaling pathway. The MAPK signaling pathway is crucial for key physiological activities such as cell growth, differentiation, proliferation, and apoptosis. As a key member of the MAPK family, p38 MAPK plays an important role in regulating inflammatory responses, dealing with cellular stress, promoting apoptosis, regulating the cell cycle, and promoting cell growth^49^. Activating the p38 MAPK signaling pathway can effectively trigger apoptosis, thereby inhibiting the proliferation of tumor cells^50^. *DUSP5,* functioning as a dual-specificity phosphatase, is pivotal in modulating the MAPK signaling cascade, particularly emphasizing the dampening of p38 MAPK activity^51^. This enzyme mitigates p38 MAPK activity by facilitating dephosphorylation events, which in turn influence cellular proliferation and differentiation processes^52^. The transcription of *DUSP5* is intricately governed by the ERK signaling axis, creating a sophisticated feedback loop that broadly impacts the MAPK signaling network, encompassing p38 MAPK. Consequently, it is implicated in the modulation of inflammatory responses and the orchestration of apoptosis^53,54^. Therefore, compound 27 effectively negatively regulates the p38 MAPK signaling pathway by stably binding to *DUSP5* and promotes apoptosis by upregulating the expression levels of *DUSP5*, thereby inhibiting the occurrence and development of bladder cancer.

## Experimental section

### Cell lines and materials

Human bladder metastatic cell carcinoma cell line (T24), human prostate cancer cell line (PC-3M), human renal cell adenocarcinoma cell line (786-0), human renal adenocarcinoma cell line (ACHN), human bladder cancer cell line (5637) were purchased from Wuhan Punuosai Life Science and Technology Co., Ltd. All cell lines were tested for STR identification and mycoplasma contamination at the time of purchase; Dulbecco’s Modified Eagle’s Medium (DMEM), fetal bovine serum, streptomycin, and penicillin G sodium were obtained from Gibco BRL (Thermo Fisher Scientific Inc., Waltham, MA, USA). Cell counting kits (CCK-8) were purchased from Sigma Company. Apoptosis Fluorescence Hoechst 33342/PI Double Staining Kit; Phosphate buffered saline (PBS); Trypsin; Penicillin; Streptomycin; Trizol reagent and RIPA lysis buffer were all purchased from Beijing Solar Biotechnology Co; enhanced chemiluminescence (ECL) was purchased from Beyotime Biotechnology Co., Ltd, Shanghai; EdU-488 cell proliferation assay kit and 1% crystalline violet staining solution, were purchased from Shanghai Biyuntian Biotechnology Co., Ltd. The positive drugs gemcitabine were purchased from Shanghai Yuanye Biotechnology Co., Ltd. Imidazole, 2-methylimidazole, 2-ethylimidazole, 4-methylimidazole, 1,2,4-triazole, tryptamine, 5-Methoxytryptamine, Triethylamine (ET3N), Di-t-Butyl Dicarbonate (Boc_2_O) were purchased from Shanghai Macklin Biochemical Co., Ltd. The EVOS M5000 fluorescence microscope was obtained from Thermo Fisher Scientific Inc. (Waltham, MA, USA).

T24 and ACHN were cultured in DMEM containing 10% fetal bovine serum, 100 U/mL penicillin, and 100 mg/mL streptomycin at 37 °C, 5% CO_2_; PC-3M, 786–0, and 5637 were cultured in RPMI-1640 medium supplemented with 10% bovine serum, 100 U/mL penicillin, and 100 mg/mL streptomycin at 37 °C with 5% CO_2_. All the test drugs were prepared as a solution with a concentration of 20 mM using dimethyl sulfoxide as a solvent.

### Chiisanosade extraction and separation

The leaves of A. *sessiliflorus* were harvested from the Changbai Mountain area of China and identified as A. *sessiliflorus* leaves by Professor Yan Zhao (College of Chinese Medicinal Materials, Jilin Agricultural University). 2 kg of dried leaves were extracted by ultrasonic extraction with 75% ethanol at a feed-to-liquid ratio of 1:8 for 24 h. Then, the ethanol extract was filtered and concentrated under reduced pressure until there was no apparent ethanol flavor. Next, gradient elution was carried out using 10%, 30%, and 50% ethanol solution with D101 resin as the stationary phase. Chiisanoside was mainly concentrated in the gradient of 50%, so it was collected and concentrated to obtain the primary product of chiisanoside (160 g, 7–8% yield). Finally, gradient elution was carried out through 200 to 300 mesh silica gel with chloroform/methanol mixture (6:1 and 3:1) as a mobile phase. The 3:1 fraction was collected, concentrated under reduced pressure, and evaporated to give purified Chiisanoside with a 75% yield. Chiisanoside was characterized by high-performance liquid chromatography (HPLC).

Preparation of chisanogenin: As shown in Scheme 1, chisanoside (955 mg, 1 mmol) was dissolved in 10% sodium hydroxide methanol solution and refluxed for 4 h. The methanol solution was recovered, and 3 mol/L hydrochloric acid solution was added to the reaction solution at pH = 5–6 and stirred for 2 h. The solvent was recovered under reduced pressure to produce the powder. Chisanogenin (364 mg, 0.75 mmol; 75% yield) was isolated by silica gel column chromatography (chloroform/methanol = 50:1/10:1).

Preparation of compound MH: Chiisanoside (955 mg, 1 mmol) was dissolved in methanol hydrochloric acid solution (3 mol/L hydrochloric acid solution) and refluxed for 2 h. At the end of the reaction, sodium hydroxide solution was added to neutralize the reaction solution to pH = 6 ~ 7, and the solvent was recovered under reduced pressure to obtain solid powder. The compound MH (381 mg, 76% yield) was then isolated by silica gel column chromatography (chloroform/methanol = 60:1/20:1).

### Cytotoxic activity assay

Firstly, cells were inoculated into 96-well plates at a density of 5 × 10^3^ cells/well. When the cells grew adherently to the wall and the cell confluence reached 60%-70%, drug treatment was carried out. Different concentrations of the drug to be tested were added to each well so that the final concentration reached the experimental set value, and then the 96-well plate was returned to the incubator at 37 °C and 5% CO₂ for 48 h. After 48 h, 10 μL of CCK-8 solution was added to each well, and the incubation was continued for 2 h in the incubator. At the end of incubation, the OD value of each well was measured at 450 nm using an enzyme marker. The cell viability inhibition rate was calculated based on the OD values, and the inhibition rate (%) = (OD value of control group − OD value of administered group) / (OD value of control group − OD value of blank group) × 100%. Finally, IC_50_ values of all tested derivatives were calculated using GraphPad Prism 8.0 software. The toxic activity of the drugs on the cells was assessed by the cell viability inhibition rate, and the higher the inhibition rate, the higher the cytotoxicity of the drug.

### Cell clone formation experiments

T24 cells were inoculated at 1 × 10^3^ cells/well in 6-well plates for 24 h. Different concentrations of drugs were added for treatment. After the culture was terminated, cells were fixed with 4% paraformaldehyde for 20 min and stained with 0.1% crystal violet for 30 min. They were then washed with PBS and observed for the formation of colonies.

### EdU cell proliferation assay

T24 cells were inoculated into six-well plates and cultured for 24 h. Three replicates of each group were then treated with different concentrations of drugs for different periods. An equal volume of prepared pre-made EdU working solution (20 μM) was added and incubated for 2 h in the cell incubator. The medium was removed, and the cells were fixed by adding 1 mL of 4% paraformaldehyde for 15 min at room temperature. The fixative was removed, and the cells were washed with PBS and permeabilized by adding PBS containing 0.3% Triton X-100 for 20 min. After washing, 0.5 mL Click reaction buffer was added and incubated for 30 min at room temperature away from light. After incubation, the Click reaction solution was discarded, and the Hoechst 33342 solution was added, incubated for 10 min away from light, and washed with PBS. Detection was performed using a fluorescence microscope.

### Cell migration assay

In 100 μL of serum-free DMEM, adjusted to a homogeneous concentration (1 × 10^5^ cells), the cell suspension was added to the upper chamber of the Transwell device. The lower chamber was filled with 600 μL of DMEM containing 30% FBS as a chemoattractant. After 24 h of incubation, the cells that had migrated or invaded the submembrane surface were rinsed twice with PBS, fixed with methanol for 30 min, and stained with 0.1% crystal violet for 20 min.

### Cell invasion assay

The steps of the cell invasion assay are the same as “4.6”; the only difference is that before preparing the cell suspension, the matrix gel should be diluted in a particular proportion, coated on the surface of the upper chamber of the Transwell bottom membrane, and then left at 37 °C for 30 min to polymerize the matrix gel.

### Cell cycle experiment

T24 cells were inoculated into six-well plates and cultured for 24 h. Then, after treatment with different concentrations of drugs for 48 h, the cells were collected and rinsed twice with pre-cooled PBS. The cells were then fixed with 70% ethanol at − 20 °C for 12–24 h. Subsequently, harvested cells were rinsed twice with PBS and stained with PI staining buffer containing RNase (Beyotime, Shanghai, China) for 30 min in an incubator. Finally, the cells were detected using a flow cytometer (BD FACSVerse).

### Hoechst 33342/PI Apoptosis kit to determine apoptosis

T24 cells were inoculated in 24-well plates at a density of 5 × 10^4^ cells/healthy density and cultured for 24 h. Different drug concentrations to be tested were added and incubated for 48 h. The cells were then incubated with the drug to be tested. The morphology of apoptotic cells was assessed using the Hoechst 33342/Propidium Iodide (PI) kit (Solarbio) according to the manufacturer’s instructions. Apoptotic cells exhibiting morphological features such as chromosome aggregation, nuclear division, and nuclear fragmentation under fluorescence microscopy were identified and counted. Red and blue hyperfluorescence can be observed in necrotic cells.

### Experimental animal

Twenty-four BALB/c nu mice (SPF grade, 7 weeks old, male, SPF (Beijing) Biotechnology Co., Ltd, SCXK 2019-0010) were taken and housed in SPF-grade animal rooms. They were acclimatized and fed for 7 days to construct the xenograft tumor model. The Experimental Ethics Committee of Jilin Agricultural University (Changchun, China) approved all animal experimental procedures (animal ethics approval number: 2023-KJT-021).

### T24 hormonal experiments

Mice were randomly assigned to each group using the random number table method. All mice were first anesthetized with 3–4% isoflurane in 100% oxygen (flow rate: 1.5 L/min) via a nose cone. After induction, the isoflurane concentration was adjusted to 1.5%-2% for maintenance. Then, 0.2 mL of T24 cell suspension (1 × 10⁷ cells/mL) was subcutaneously injected into the left forelimb axilla of each anesthetized mouse. When the tumor size reached 100 mm^3^, the mice were randomly divided into 4 groups (n = 6): the positive drug group administered intraperitoneally with gemcitabine (40 mg/kg); the Compound 27 low-dose group administered intraperitoneally (10 mg/kg); the Compound 27 high-dose group administered intraperitoneally (20 mg/kg); and the model group administered intraperitoneally with the same volume of saline, and the tumor volume of the mice was recorded every day during the experiment. Mice were dosed every three days and, after 11 days of treatment, mice were killed by cervical dislocation on day 15 and organs and tumour tissues were collected separately. This study was carried out adhered to the ARRIVE Guidelines^55^ All experiments were conducted in accordance with relevant guidelines and regulations, complying with the International Guiding Principles for Biomedical Research Involving Animals^56^.

### Histopathological analysis

Immediately after collection, tumor and organ tissue specimens were fixed in a 4% paraformaldehyde solution. The purpose of fixation is to preserve the morphological structure of the tissue cells and prevent them from changing during subsequent processing. After completion of fixation, the tissues were sequentially placed in ethanol solutions with different concentration gradients (e.g., 70%, 80%, 90%, 95%, and 100%) for dehydration. The dehydration process progressively removes water from the tissues so that the subsequent paraffin wax can fully infiltrate the tissues. After dehydration, the tissue was paraffin-embedded, where the tissue block was embedded in paraffin to form a firm paraffin block for subsequent sectioning. The paraffin-embedded tissue was sliced into thin slices of approximately 4–6 μm thickness using a slicer. When slicing, it is important to ensure the integrity and flatness of the slices to avoid folds or breaks. The cut slices were subjected to dewaxing, and the paraffin slices were sequentially soaked in xylene solution to remove the paraffin. After dewaxing, the slices were hydrated by placing them in graded concentrations of alcohol (100%, 95%, 90%, 80%, 70%) to return the tissue to a hydrated state for subsequent staining. For staining, the sections were first placed in hematoxylin staining solution for 5–10 min to stain the nuclei and other structures blue. The sections were then rinsed with running water to remove excess hematoxylin stain. Next, the sections were stained in eosin stain for 2–5 min to colour the cytoplasm and other structures red. After the staining was completed, the sections were again rinsed with running water to remove excess eosin stain. Finally, the stained sections were dehydrated by placing them sequentially in 95% and 100% alcohol, and then treated with xylene for transparency to make the sections clearer. After the treatment was completed, the sections were sealed with neutral gum and fixed on slides for observation under the microscope. Under the microscope, the cellular morphology and structural changes of the tumor tissues and organ tissues were observed to assess the growth of the tumour, the degree of apoptosis and necrosis, and the effect of drugs on the tissues. Comparative observations of tissue sections from different groups were used to analyse the effectiveness of compound 27 in the treatment of bladder cancer and whether it had toxic side effects on normal organs.

### Survival analysis

Twenty mice were divided into four groups: the model group, the positive drug group, the low-dose group, and the high-dose group. Each group was given the same dose as ‘4.12’. The number of mice deaths was recorded daily, the test was terminated after 75 days of mice loaded with the drug, and the experimental data were statistically analysed.

### Statistical analysis

GraphPad Prism 8.0 was used for statistical data analysis, and its standard deviation was used as the mean deviation (SEM). Three or more groups of gradual difference statistical analysis use one-way ANOVA. The Student t-test determined statistical significance except for the RNA-sequence analysis (Fisher exact test). *P* < 0.05 was considered as a statistically significant difference.

### RNA sequencing data analysis

RNA sequencing data from the TCGA-BLCA (bladder urothelial carcinoma) cohort were obtained through the TCGA database (https://xenabrowser.net). Following stringent quality control measures and data preprocessing, a total of 18 paired samples were included in the analysis, consisting of primary bladder cancer tissues and their matched adjacent normal tissues. Differential gene expression analysis was performed using the DESeq2 package. This package implements a negative binomial distribution model that effectively accounts for biological variability and manages outliers. Differentially Expressed Genes (DEGs) were identified using the following criteria: adjusted p-value < 0.05 and absolute log2 fold change (|log2FC|) > 1. The Benjamini–Hochberg method was applied for multiple testing corrections—functional Enrichment Analysis. Functional enrichment analysis was conducted using the clusterProfiler package to elucidate the biological functions and pathways associated with the identified DEGs. This analysis encompassed both Gene Ontology (GO) enrichment analysis across three categories (biological process, cellular component, and molecular function) and Kyoto Encyclopedia of Genes and Genomes (KEGG) pathway analysis^28–30^.

### Clinical correlation analysis

The UALCAN database (http://ualcan.path.uab.edu) is used to validate target genes and is a platform capable of identifying specific candidate biomarkers for tumor subgroups. To conduct a clinical correlation analysis of key genes using the UALCAN database, we input the key genes, set to use the TCGA database, select bladder cancer as the disease, and set conditions such as "Gene expression based on individual cancer" to obtain the expression levels of the key genes.

### Survival analysis

The Kaplan–Meier plotter (https://kmplot.com/analysis/) contains microarray gene expression data and survival information data from databases such as the Gene Expression Omnibus (GEO) and The Cancer Genome Atlas (TCGA). The gene expression and survival data information of bladder cancer patients are also included. We used the Kaplan–Meier plotter to evaluate the prognostic value of the key gene expressions in terms of overall survival (OS) and relapse-free survival (RFS).

### Immune infiltration analysis

The TIMER database (https://cistrome.shinyapps.io/timer/) was used to perform a comprehensive and systematic immune infiltration analysis. This database systematically analyzes the correlation between the expression of key genes and the abundance of cellular immune infiltration. The TIMER algorithm was used to calculate the expression of key genes and the abundance of immune infiltration of six immune cells (B cells, CD4^+^ T cells, CD8^+^ T cells, neutrophils and macrophages, and dendritic cells).

### Molecular docking

The 3D structure of compound 27 was drawn using Chem3D, energy-optimized, and stored in a mol2 file. QPBQT files were exported using Autodock tool 1.5.6. The receptor protein *DUSP5* (Protein Data Bank: 2G6Z) was downloaded from the PDB (http://www.rcsb.org/), the docking centers were located, and water molecules and original ligands were removed using Discovery Studio Visualizer 2020. The processed receptors and ligands were imported into AutoDock Tools 1.5.6 to generate PDBQT files. Molecular docking was performed using the AutoDock program, and the results were visualized using Discovery Studio Visualizer 2020.

### Quantitative real-time RT-PCR

The total RNA samples were extracted using TRIzol reagent using the methodology previously described in the literature. Subsequently, the total RNA was reverse-transcribed into cDNA using an RT-qPCR kit (TransGen Biotech, Beijing, China). The PCR conditions were as follows: denaturation at 94 °C for 30 s, followed by 45 cycles of denaturation at 94 °C for 5 s, annealing at 60 °C for 15 s, and extension at 72 °C for 10 s. The relative expression levels of the target genes were calculated based on the cycle threshold (Ct) values as follows: △*C*_t_ = *C*_t_ (target gene)  − *C*_t_ (*GAPDH*); △△*C*_t_ = △*C*_t_ (treatment) − △*C*_t_ (control); Fold change = 2 (^△△*C*t^). The sequences of the primers used for the target genes are presented in Table 2.

**Table 2:** Sequences of the primers for real-time RT-PCR.

Gene	Forward primer (5′–3′)	Reverse primer (5′–3′)
*DUSP5*	GAAGATATGGGGCAACTGGA	ATGCACGTGTTCAGCTTGAG
*SCG2*	ACCAGACCTCAGGTTGGAAAA	ACCAGACCTCAGGTTGGAAAA
*GAPDH*	GTCAAGGCTGAGAACGGGAA	AAATGAGCCCCAGCCCTTCTC

### Molecular dynamics simulation

Molecular dynamics simulations of protein–ligand complexes obtained by molecular docking were performed at 100 ns using the Gromacs package. The force field used to treat the proteins and ligands was charmm36. A TIP3P water model was added to the system, and a water box with a periodic boundary of 1.2 nm was created. Then, water molecules and ions were added to the water box, and energy minimization was performed to treat the system, followed by temperature- and pressure-controlled equilibration. After energy minimization and equilibration of the system, the molecular dynamics simulations were carried out without any constraints at a time step of 2 fs for 100 ns. At the same time, the structural coordinates were saved every ten ps. Finally, we analyzed the root mean square deviation (RMSD) and root mean square fluctuation (RMSF) in the molecular dynamics simulation trajectories of the complexes.

## Conclusion

Compound 27 effectively modulates the p38 MAPK signaling pathway by stably binding to *DUSP5* and promotes cell apoptosis by upregulating the expression level of *DUSP5*, thereby inhibiting the occurrence and development of bladder cancer. This discovery provides potential molecular targets and compound candidates for developing new treatment strategies for bladder cancer, with significant prospects for clinical application. Future research will further explore the pharmacological properties, dose–effect relationships, and potential applications in bladder cancer treatment of compound 27.

## Supplementary Information

Supplementary Information.

## Data Availability

The raw data of publicly available datasets were downloaded from TCGA dataset (http://cancergenome.nih.gov/). Further inquiries can be directed to the corresponding authors.

## Abbreviations

CCK-8: Cell counting kit-8
EdU: EdU fluorescence labeling for cell proliferation
KEGG: Kyoto Encyclopedia of Genes and Genomes
GO: Gene Ontology
RT-qPCR: Quantitative real-time PCR
LEU: Leucine
FBS: Fetal bovine serum
DMEM: Dulbecco’s modified eagle medium
ET3N: Triethylamine
Boc_2_O: Di-tert-butyl dicarbonate
