# Supplementary material for: Design and synthesis of 3,4-seco-lupane triterpene-tryptamine derivatives and revealing their anti-bladder cancer mechanisms by combining TCGA and transcriptomic approaches
Source: Sci Rep. 2025 Jun 5;15:19723. doi: 10.1038/s41598-025-04855-y (PMC12141488; doi:10.1038/s41598-025-04855-y)

***Supplementary Material***

**Qinglong Chi^.a^**, **Hongbo Teng^.b^**, **Yaru Zhao^.b^**, **Xv Wang^.b^**, **Jiexin Zhang^.b^** , **Huiyue Shen^.b^**, **Xuan He^.b^**, **Yan Zhao^.b^**, **Chunxi Wang^.a*^**

^a^Department of Urology, The First Hospital of Jilin University, Changchun 130021, Jilin, China

^b^College of Chinese Medicinal Materials, Jilin Agricultural University, Changchun, Jilin Province, China

*** Correspondence:**

Corresponding Author: Department of Urology, The First Hospital of Jilin University, Changchun 130021, Jilin, China

E-mail addresses: [chunxi@jlu.edu.cn](mailto:chunxi@jlu.edu.cn)

Table S1. IC_50_ Values of Compounds against Five Tumor Cell Lines.

| **compound** | **IC_50_ (μM)** | | | | | **compound** | **IC_50_ (μM)** | | | | |
| --- | --- | --- | --- | --- | --- | --- | --- | --- | --- | --- | --- |
|  | **PC-3M** | **786-O** | **ACHN** | **T24** | **5637** |  | **PC-3M** | **786-O** | **ACHN** | **T24** | **5637** |
| **chiisanogenin** | **134.2±12.32** | **159.38±7.28** | **182.06±16.34** | **106.35±4.36** | **145.23±12.33** | **compound MH** | **103.43±15.35** | **85.02±7.56** | **73.95±4.37** | **121.7±18.89** | **189.36±13.92** |
| **1** | **109.4±10.30** | **126.56±7.31** | **174.63±14.14** | **96.20±8.56** | **105.45±9.55** | **46** | **47.55±3.56** | **63.98±5.33** | **57.82±6.15** | **67.89±12.34** | **45.12±3.33** |
| **2** | **24.78±2.64** | **21.76±2.98** | **23.40±2.02** | **22.44±2.31** | **32.11±2.33** | **47** | **35.62±2.57** | **34.51±3.01** | **38.65±4.22** | **49.78±6.232** | **89.36±7.46** |
| **3** | **15.95±2.25** | **27.35±3.49** | **38.55±3.08** | **16.42±3.57** | **16.45±0.12** | **48** | **34.44±1.25** | **58.62±5.37** | **74.31±6.05** | **47.89±9.45** | **42.36±3.98** |
| **4** | **18.46±1.89** | **10.36±1.23** | **10.97±0.78** | **16.34±2.45** | **20.12±1.89** | **49** | **30.62±1.55** | **46.97±3.98** | **48.01±5.47** | **45.32±7.56** | **30.36±2.43** |
| **5** | **12.33±2.33** | **27.37±1.10** | **38.46±3.45** | **11.45±1.48** | **12.45±1.03** | **50** | **24.91±2.31** | **46.72±3.79** | **69.81±6.30** | **36.45±5.68** | **48.78±3.90** |
| **6** | **33.95±0.88** | **29.34±3.48** | **47.29±4.16** | **32.56±3.44** | **45.78±4.12** | **51** | **43.21±2.88** | **63.40±0.45** | **76.58±5.52** | **56.37±6.45** | **69.30±5.32** |
| **7** | **6.27±0.65** | **10.27±3.09** | **18.47±1.63** | **5.43±1.22** | **8.45±0.45** | **52** | **11.61±1.06** | **8.94±0.78** | **9.63±1.63** | **5.45±1.08** | **10.57±0.55** |
| **8** | **5.11±0.51** | **6.28±0.43** | **8.46±0.48** | **4.90±0.51** | **8.78±1.04** | **53** | **10.83±0.84** | **7.34±0.28** | **6.37±5.36** | **4.34±0.66** | **9.58±1.01** |
| **9** | **3.85±0.35** | **3.81±0.28** | **9.50±0.82** | **3.64±0.45** | **4.78±0.12** | **54** | **3.23±0.14** | **8.42±0.67** | **9.26±0.41** | **4.06±1.23** | **8.60±0.45** |
| **10** | **82.20±5.25** | **92.38±6.72** | **86.49±9.07** | **96.03±8.21** | **104.50±9.89** | **55** | **53.29±4.56** | **64.37±6.22** | **62.43±8.15** | **65.23±8.39** | **89.02±7.34** |
| **11** | **28.23±2.35** | **19.98±1.45** | **18.65±1.56** | **26.64±3.24** | **36.48±2.55** | **56** | **44.28±1.85** | **40.78±3.99** | **52.01±7.42** | **52.32±7.89** | **47.15±3.97** |
| **12** | **30.01±1.36** | **21.87±1.33** | **34.57±3.05** | **15.67±3.01** | **12.45±0.98** | **57** | **35.95±2.58** | **47.06±4.34** | **54.09±5.01** | **36.34±4.52** | **40.18±3.22** |
| **13** | **15.53±1.33** | **10.28±1.34** | **13.40±1.24** | **15.32±2.78** | **18.56±1.11** | **58** | **25.04±1.89** | **32.87±3.11** | **41.52±3.46** | **28.31±3.89** | **28.69±1.56** |
| **14** | **12.06±0.55** | **19.34±1.78** | **26.50±2.54** | **13.78±1.56** | **22.03±2.13** | **59** | **25.95±3.14** | **20.53±1.78** | **31.72±2.28** | **27.56±1.67** | **28.63±2.54** |
| **15** | **18.09±0.91** | **24.83±2.35** | **35.46±3.17** | **24.56±2.80** | **20.02±1.87** | **60** | **45.31±2.36** | **50.48±5.19** | **45.68±3.05** | **48.98±4.01** | **56.69±3.23** |
| **16** | **6.644±1.20** | **8.74±0.76** | **13.06±1.08** | **5.2±0.64** | **8.27±1.12** | **61** | **15.66±0.69** | **14.11±0.82** | **10.59±0.95** | **5.30±0.98** | **8.36±0.45** |
| **17** | **6.31±1.05** | **3.29±0.53** | **8.71±0.50** | **4.15±0.69** | **9.23±1.33** | **62** | **15.96±1.36** | **12.76±1.89** | **8.61±1.01** | **4.24±0.94** | **9.47±1.03** |
| **18** | **3.77±0.25** | **3.16±0.17** | **6.02±0.46** | **3.21±0.32** | **6.48±0.23** | **63** | **2.72±0.35** | **7.51±0.33** | **8.81±1.82** | **2.98±0.45** | **8.04±0.21** |
| **19** | **14.53±1.21** | **16.26±1.39** | **21.84±2.04** | **18.78±3.45** | **31.23±2.98** | **64** | **44.09±9.63** | **39.44±3.12** | **25.77±2.10** | **42.56±5.02** | **58.96±4.23** |
| **20** | **11.84±1.11** | **10.35±1.03** | **13.40±1.02** | **20.03±2.19** | **25.23±2.01** | **65** | **30.57±4.52** | **31.85±2.77** | **37.15±2.25** | **45.35±4.23** | **60.48±4.03** |
| **21** | **12.42±0.89** | **26.73±3.06** | **41.29±3.54** | **12.02±1.23** | **11.27±1.05** | **66** | **37.84±0.84** | **29.94±2.12** | **27.11±1.84** | **40.23±3.93** | **45.64±3.97** |
| **22** | **14.82±1.81** | **27.39±2.33** | **11.38±1.25** | **13.21±1.28** | **11.89±1.04** | **67** | **30.08±1.26** | **29.36±2.45** | **34.84±2.83** | **38.56±7.23** | **48.02±3.45** |
| **23** | **12.52±2.35** | **11.67±1.09** | **25.13±1.58** | **12.85±1.89** | **25.36±2.06** | **68** | **23.85±1.26** | **28.11±2.33** | **32.07±6.42** | **35.78±7.12** | **20.15±1.09** |
| **24** | **54.33±2.89** | **49.75±5.66** | **38.27±4.54** | **28.36±1.56** | **36.45±2.13** | **69** | **32.29±2.45** | **38.22±3.55** | **31.46±2.75** | **42.35±8.04** | **60.36±4.03** |
| **25** | **8.93±0.51** | **6.85±0.59** | **11.30±1.25** | **3.23±0.89** | **7.41±0.04** | **70** | **13.87±1.274** | **13.90±1.19** | **17.70±1.26** | **3.52±0.87** | **8.69±0.67** |
| **26** | **9.81±1.25** | **8.35±1.09** | **8.26±0.79** | **2.04±0.66** | **6.45±0.52** | **71** | **11.77±1.84** | **5.24±1.12** | **12.08±1.86** | **3.32±1.32** | **14.04±1.34** |
| **27** | **6.35±0.83** | **2.67±0.22** | **5.48±0.47** | **1.121±0.25** | **4.56±0.12** | **72** | **2.76±0.16** | **2.09±0.19** | **4.20±0.62** | **2.29±0.23** | **8.14±0.17** |
| **28** | **16.09±1.35** | **23.89±2.19** | **24.59±2.25** | **85.56±7.89** | **98.20±4.98** | **73** | **29.01±2.11** | **46.38±3.78** | **57.29±6.43** | **34.56±3.07** | **47.02±2.98** |
| **29** | **23.24±2.52** | **38.40±3.46** | **32.92±2.89** | **25.64±1.78** | **21.30±1.98** | **74** | **19.74±1.58** | **46.03±3.99** | **40.26±1.39** | **28.45±2.45** | **21.03±1.97** |
| **30** | **13.96±1.87** | **24.31±2.08** | **34.94±3.59** | **14.45±1.98** | **18.95±1.03** | **75** | **20.93±1.99** | **39.24±2.79** | **30.56±1.88** | **21.23±1.78** | **30.18±2.07** |
| **31** | **12.25±1.58** | **32.76±2.97** | **29.88±2.58** | **14.12±1.45** | **17.05±1.34** | **76** | **18.36±0.69** | **43.95±3.33** | **58.60±2.53** | **24.35±2.03** | **39.04±2.56** |
| **32** | **12.93±2.35** | **23.09±2.26** | **21.98±1.78** | **14.02±2.12** | **15.26±1.29** | **77** | **19.56±0.97** | **35.64±2.95** | **39.95±6.09** | **27.45±1.55** | **25.56±1.98** |
| **33** | **56.04±4.21** | **47.58±4.37** | **45.81±4.05** | **32.45±3.02** | **56.45±3.24** | **78** | **27.42±1.29** | **27.16±2.33** | **35.63±5.42** | **30.21±2.89** | **36.98±3.87** |
| **34** | **11.99±0.88** | **7.09±0.56** | **9.73±0.87** | **4.53±1.03** | **8.04±0.23** | **79** | **13.99±1.36** | **15.73±1.11** | **8.68±1.29** | **4.54±0.81** | **8.45±0.51** |
| **35** | **9.18±0.75** | **10.45±0.91** | **7.16±0.47** | **4.16±0.72** | **5.23±0.34** | **80** | **3.38±0.58** | **7.39±0.36** | **6.57±0.40** | **3.59±0.99** | **6.93±0.98** |
| **36** | **3.79±0.65** | **7.84±0.68** | **8.93±0.97** | **3.89±0.45** | **4.12±0.11** | **81** | **3.84±0.15** | **4.02±0.21** | **5.79±0.85** | **3.04±0.55** | **7.05±0.13** |
| **37** | **17.29±1.87** | **26.97±2.44** | **38.27±3.69** | **91.23±4.23** | **84.78±4.33** | **82** | **16.65±1.13** | **23.01±2.11** | **31.45±3.01** | **25.89±0.12** | **22.48±2.01** |
| **38** | **11.76±2.01** | **20.93±1.97** | **32.10±3.04** | **28.23±2.89** | **24.56±1.46** | **83** | **13.97±0.85** | **32.19±2.55** | **38.81±4.79** | **15.82±0.12** | **18.47±1.19** |
| **39** | **8.63±0.89** | **24.80±2.11** | **30.56±2.89** | **15.67±3.02** | **12.36±1.08** | **84** | **10.31±1.52** | **34.65±2.67** | **42.47±1.50** | **14.78±0.13** | **10.89±0.89** |
| **40** | **13.86±0.45** | **25.09±2.34** | **27.46±2.48** | **15.78±2.89** | **31.02±2.29** | **85** | **12.66±1.33** | **35.84±3.04** | **47.11±1.46** | **10.45±0.09** | **18.56±0.12** |
| **41** | **13.87±0.87** | **33.76±3.15** | **48.26±4.56** | **12.45±1.78** | **13.69±0.89** | **86** | **11.45±0.28** | **27.36±1.98** | **38.51±4.22** | **9.56±0.66** | **8.14±0.48** |
| **42** | **19.94±1.57** | **52.03±4.99** | **59.37±5.02** | **24.02±2.56** | **32.67±2.98** | **87** | **23.46±1.29** | **49.24±4.22** | **47.22±8.83** | **8.56±0.44** | **8.25±0.56** |
| **43** | **8.69±0.54** | **9.17±0.87** | **9.98±0.87** | **5.82±0.65** | **8.69±0.11** | **88** | **15.72±1.35** | **7.55±0.12** | **9.53±0.28** | **5.05±1.12** | **9.47±0.17** |
| **44** | **6.38±0.87** | **8.56±0.58** | **6.95±0.77** | **4.78±0.59** | **8.48±0.67** | **89** | **9.64±0.96** | **9.47±0.81** | **10.92±1.41** | **4.23±0.84** | **8.15±0.43** |
| **45** | **4.16±0.12** | **5.35±0.43** | **6.04±0.54** | **3.65±0.45** | **5.86±0.21** | **90** | **3.65±0.26** | **6.84±0.33** | **7.01±6.28** | **3.58±0.01** | **9.17±0.51** |

Table S1. IC_50_ Values of Compounds against Five Tumor Cell Lines.

Figures S1. *HPLC tracing of compound* ***8****.*

Figures S2. *^13^C and ^1^H NMR of* *compound* ***8****.*

Figures S3. *HPLC tracing of compound* ***9****.*

Figures S4. *^13^C and ^1^H NMR of compound* ***9****.*

Figures S5. *HPLC tracing of compound* ***17****.*

Figures S6. *^13^C and ^1^H NMR of compound* ***17****.*

Figures S7. *HPLC tracing of compound* ***18****.*

Figures S8. *^13^C and ^1^H NMR of compound* ***18****.*

Figures S9. *HPLC tracing of compound* ***26****.*

Figures S10. *^13^C and ^1^H NMR of compound* ***26****.*

Figures S11. *HPLC tracing of compound* ***27****.*

Figures S12. *^13^C and ^1^H NMR of compound* ***27****.*

Figures S13. *HPLC tracing of compound* ***35****.*

Figures S14. *^13^C and ^1^H NMR of compound* ***35****.*

Figures S15. *HPLC tracing of compound* ***36****.*

Figures S16. *^13^C and ^1^H NMR of compound* ***36****.*

Figures S17. *HPLC tracing of compound* ***44****.*

Figures S18. *^13^C and ^1^H NMR of compound* ***44****.*

Figures S19. *HPLC tracing of compound* ***45****.*

Figures S20. *^13^C and ^1^H NMR of compound* ***45****.*

Figures S21. *HPLC tracing of compound* ***53.***

Figures S22. *^13^C and ^1^H NMR of compound* ***53****.*

Figures S23. *HPLC tracing of compound* ***54.***

Figures S24. *^13^C and ^1^H NMR of compound* ***54****.*

Figures S25. *HPLC tracing of compound* ***62.***

Figures S26. *^13^C and ^1^H NMR of compound* ***62****.*

Figures S27. *HPLC tracing of compound* ***63.***

Figures S28. *^13^C and ^1^H NMR of compound* ***63****.*

Figures S29. *HPLC tracing of compound* ***71.***

Figures S30. *^13^C and ^1^H NMR of compound* ***71****.*

Figures S31. *HPLC tracing of compound* ***72.***

Figures S32. *^13^C and ^1^H NMR of compound* ***72****.*

Figures S33. *HPLC tracing of compound* ***80.***

Figures S34. *^13^C and ^1^H NMR of compound* ***80****.*

Figures S35. *HPLC tracing of compound* ***81.***

Figures S36. *^13^C and ^1^H NMR of compound* ***81****.*

Figures S37. *HPLC tracing of compound* ***89.***

Figures S38. *^13^C and ^1^H NMR of compound* ***89****.*

Figures S39. *HPLC tracing of compound* ***90.***

Figures S40. *^13^C and ^1^H NMR of compound* ***90****.*

Figure S1. *HPLC tracing of compound* ***8****.*


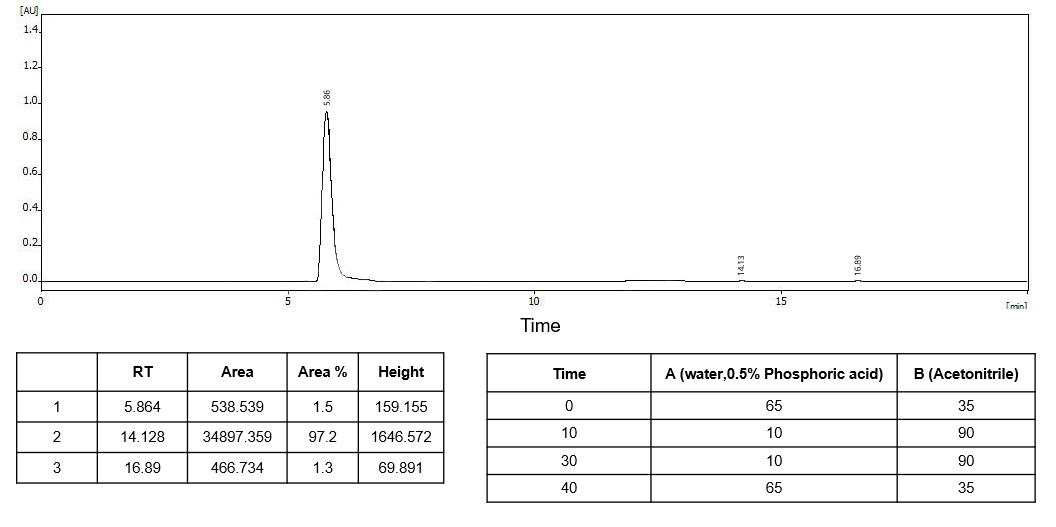


Figures S2. *^13^C and ^1^H NMR of compound* ***8****.*


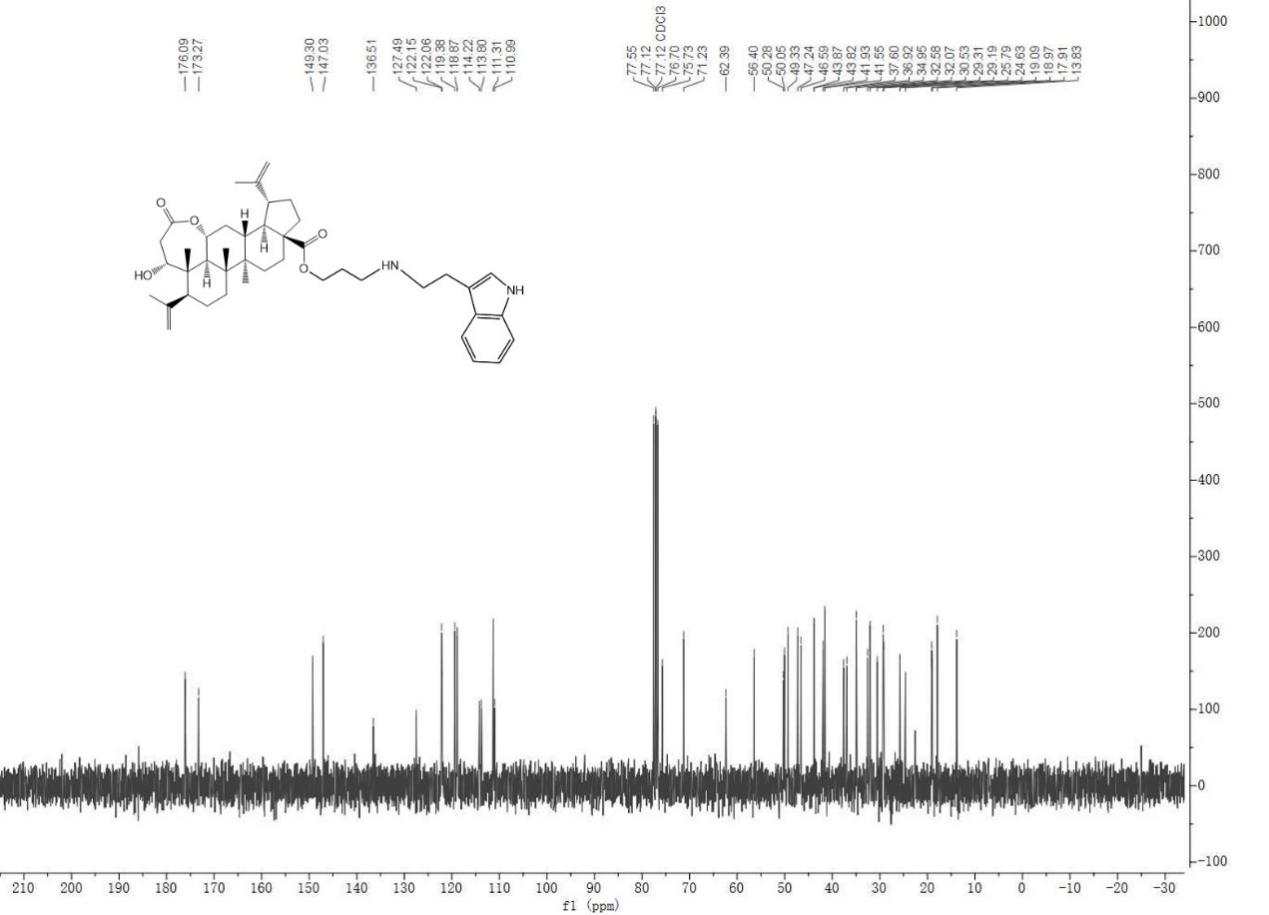


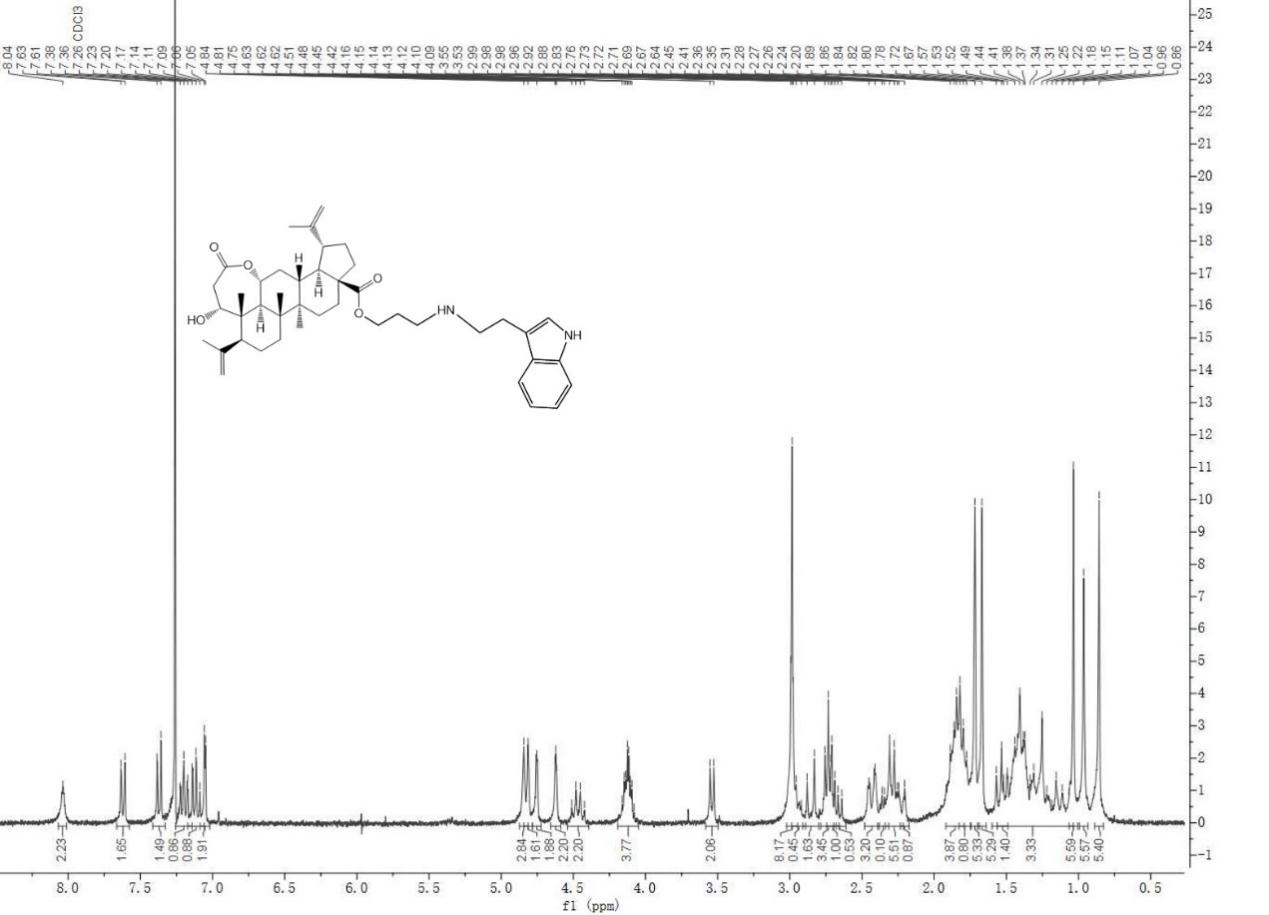


Figure S3. *HPLC tracing of compound* ***9****.*


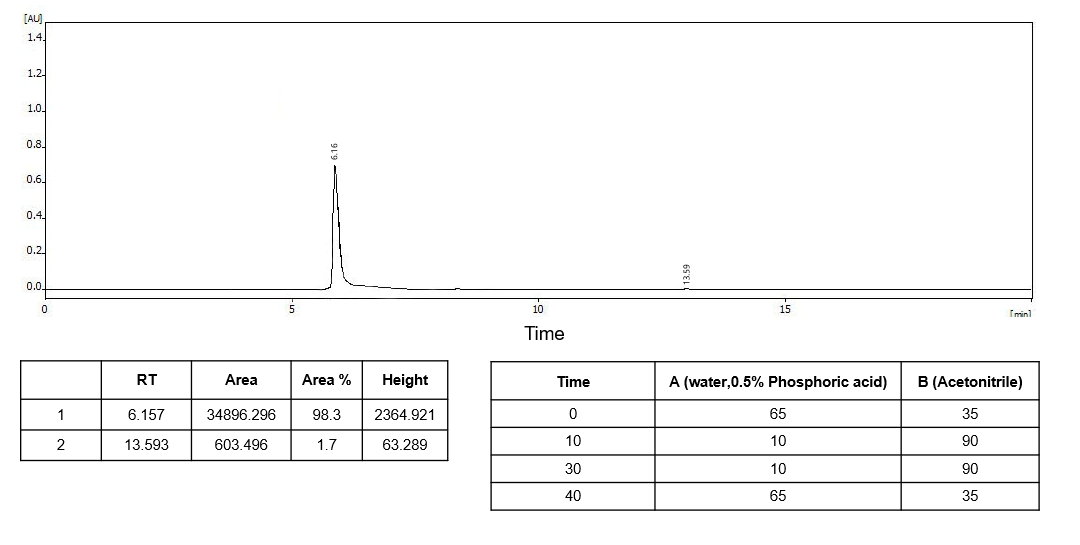


Figures S4. *^13^C and ^1^H NMR of compound* ***9****.*


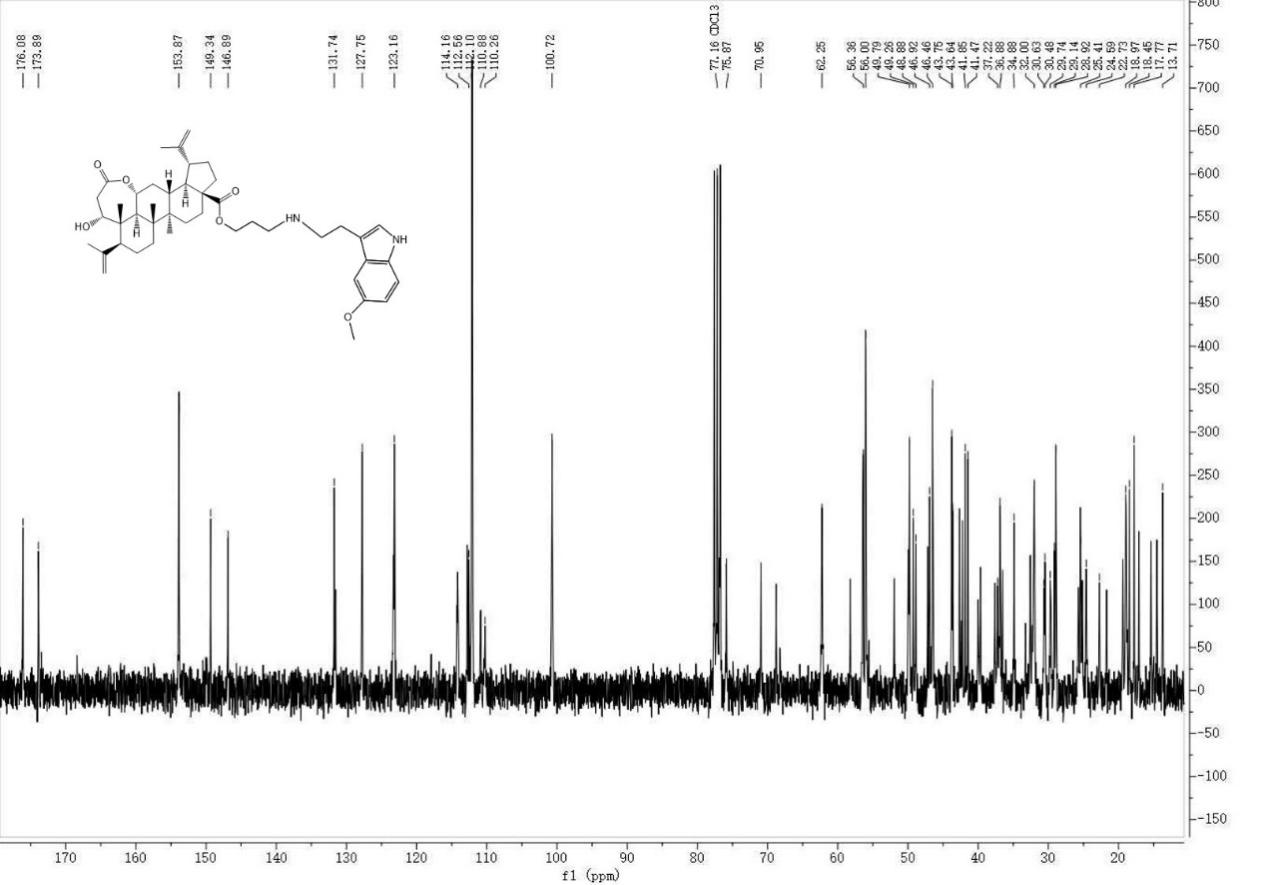


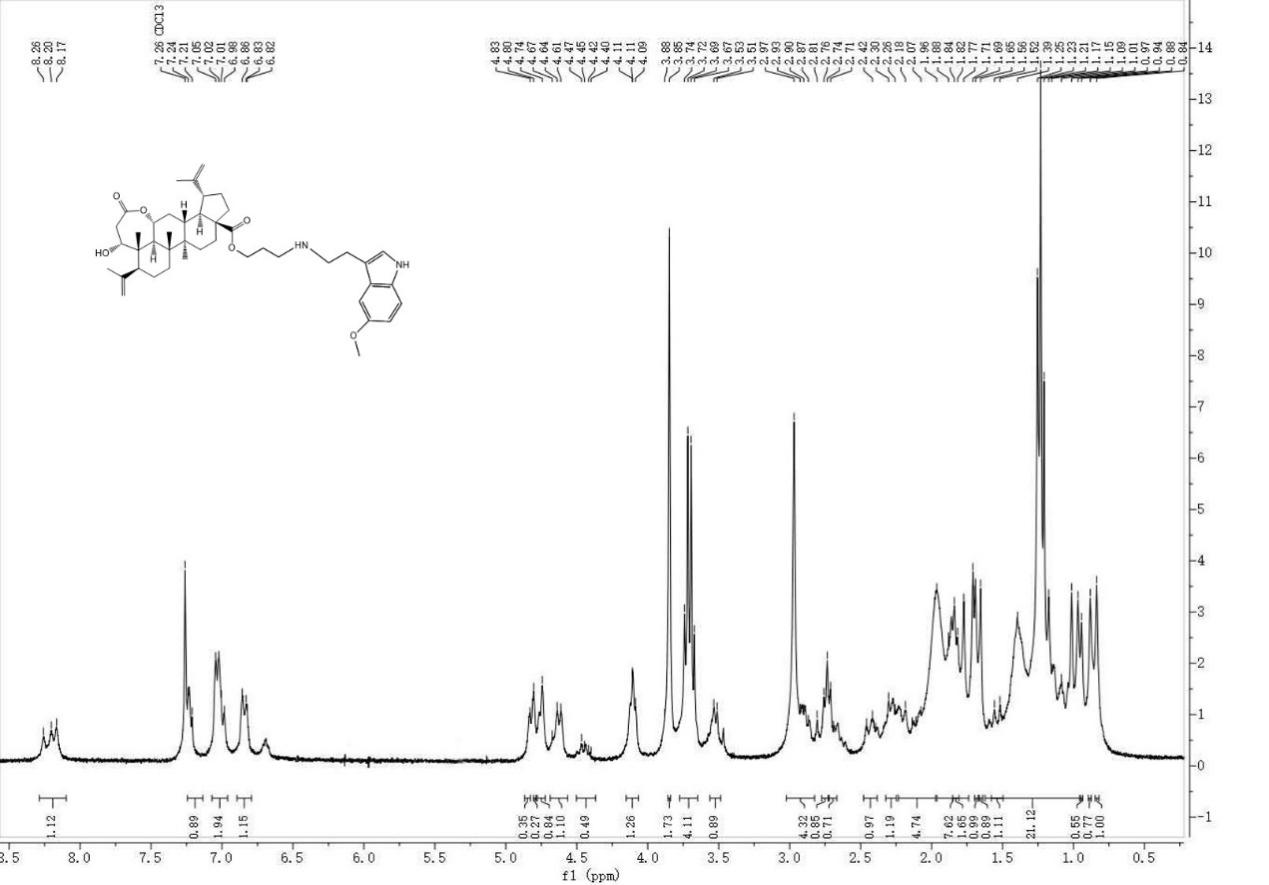


Figure S5. *HPLC tracing of compound****17.***


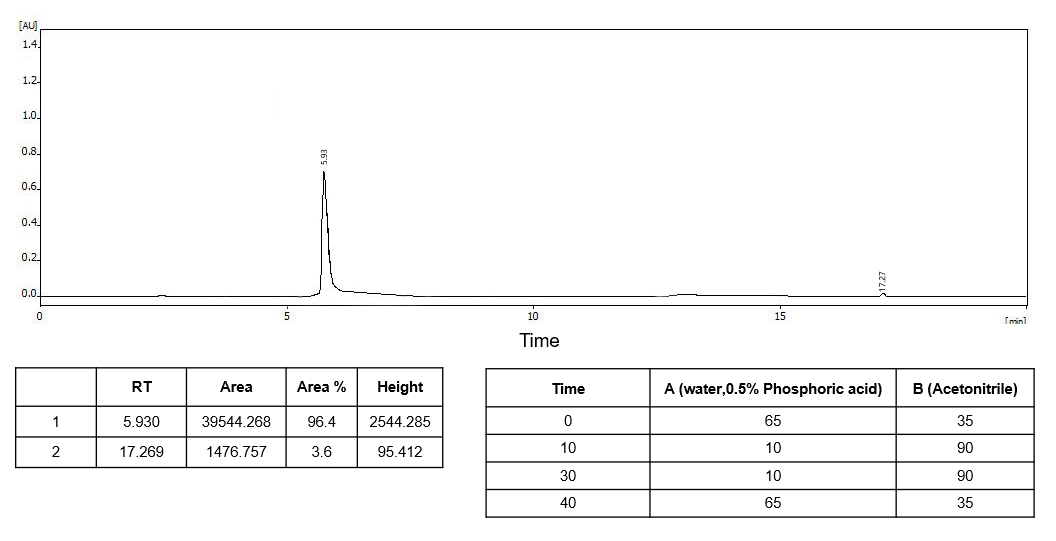


Figures S6. *^13^C and ^1^H NMR of compound* ***17****.*


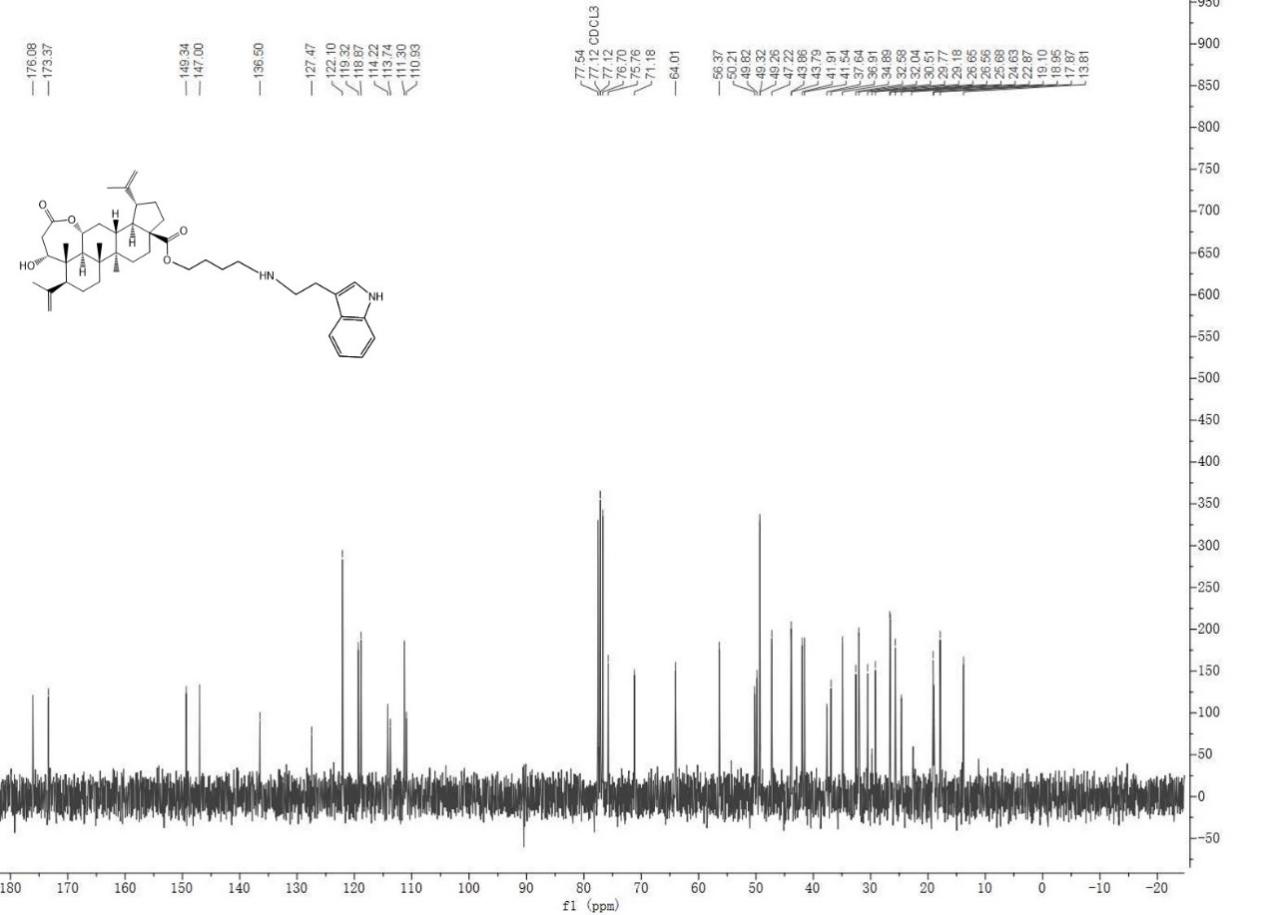


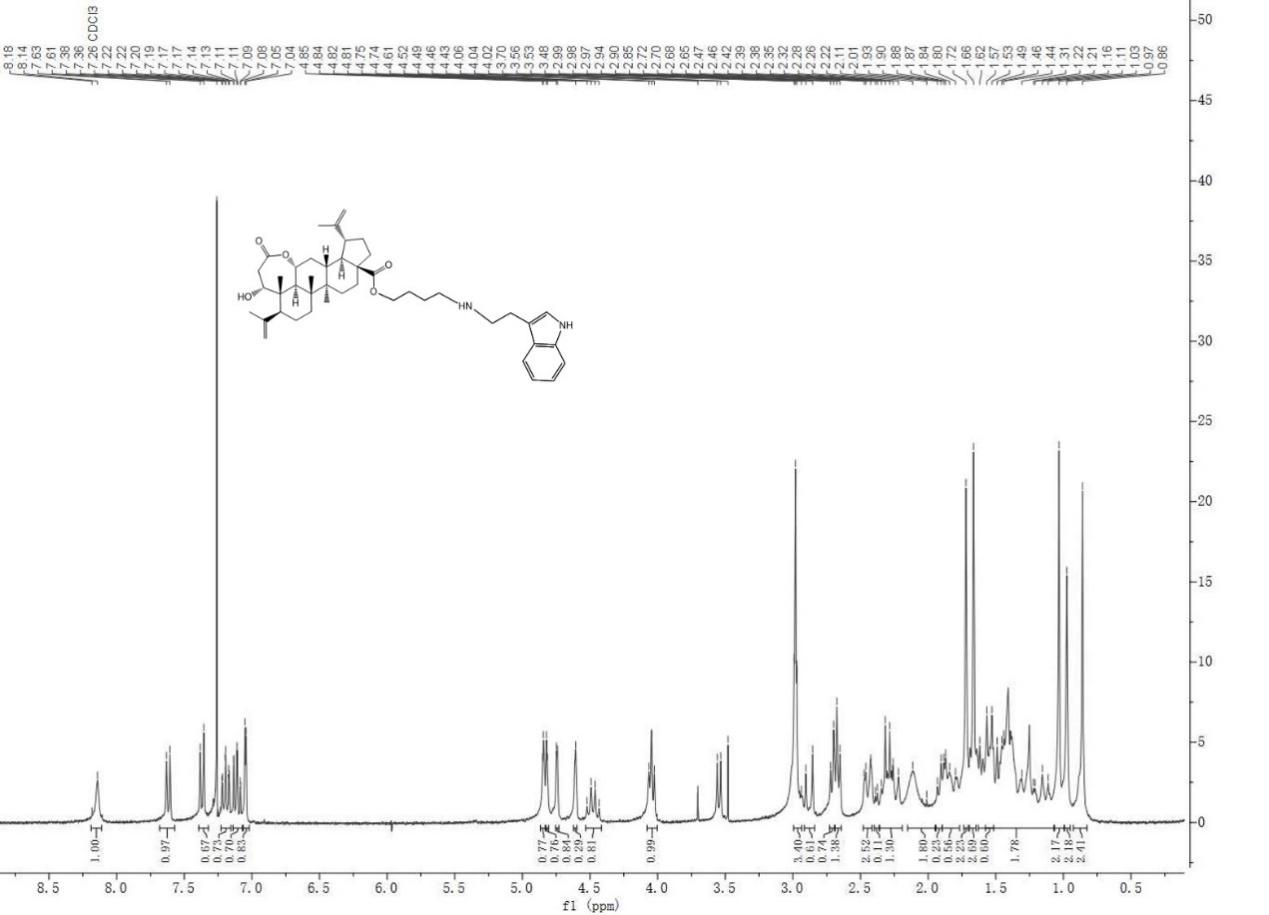


Figure S7. *HPLC tracing of compound* ***18.***


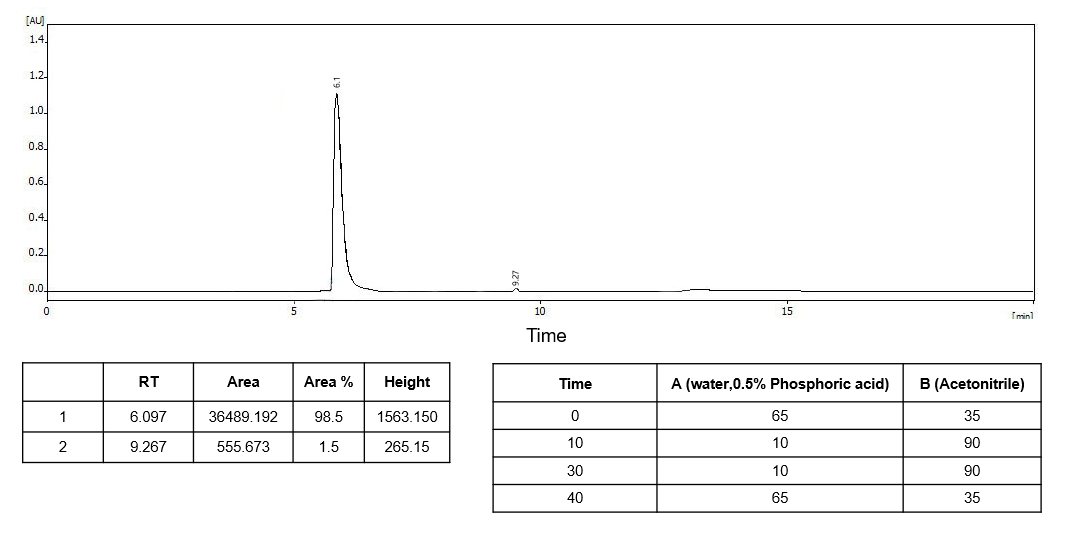


Figures S8. *^13^C and ^1^H NMR of compound* ***18****.*


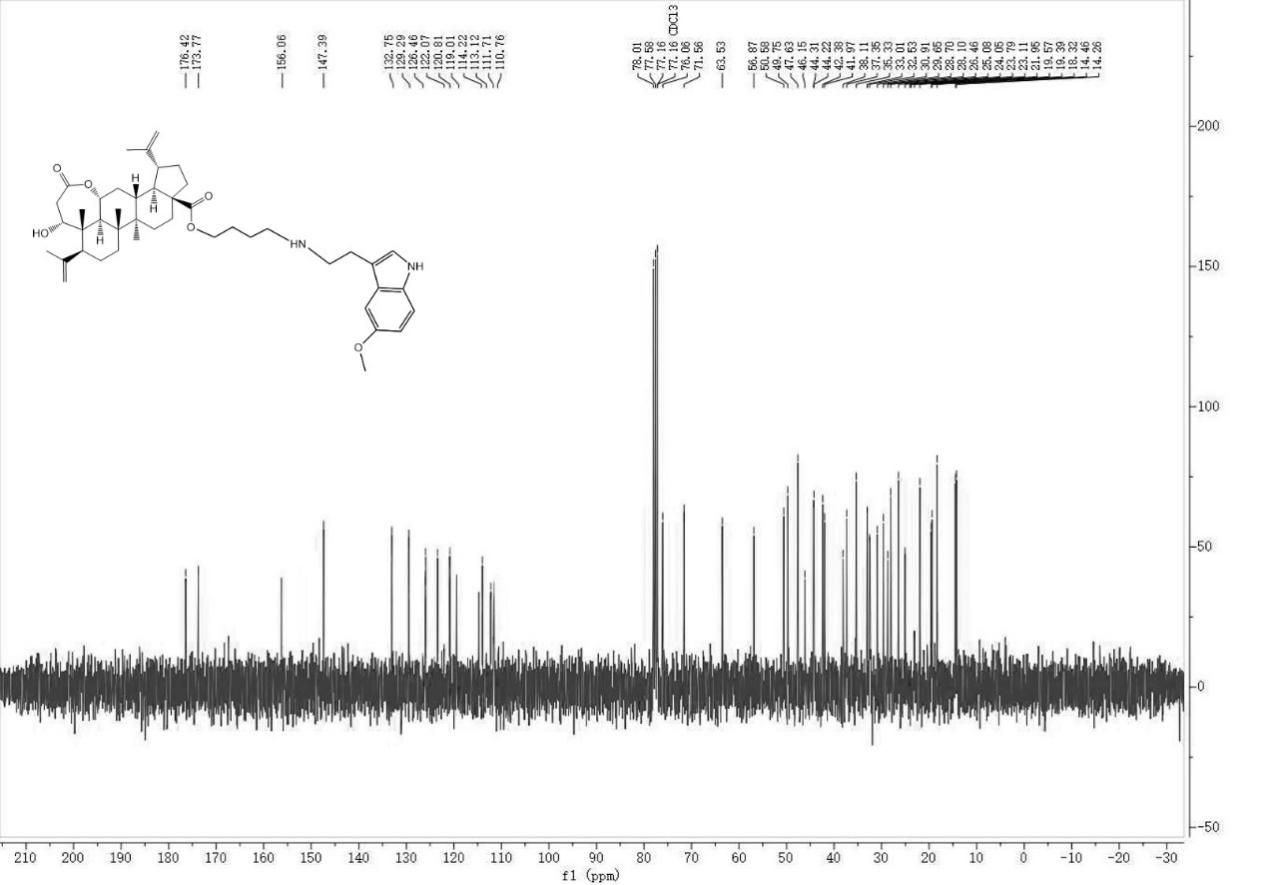


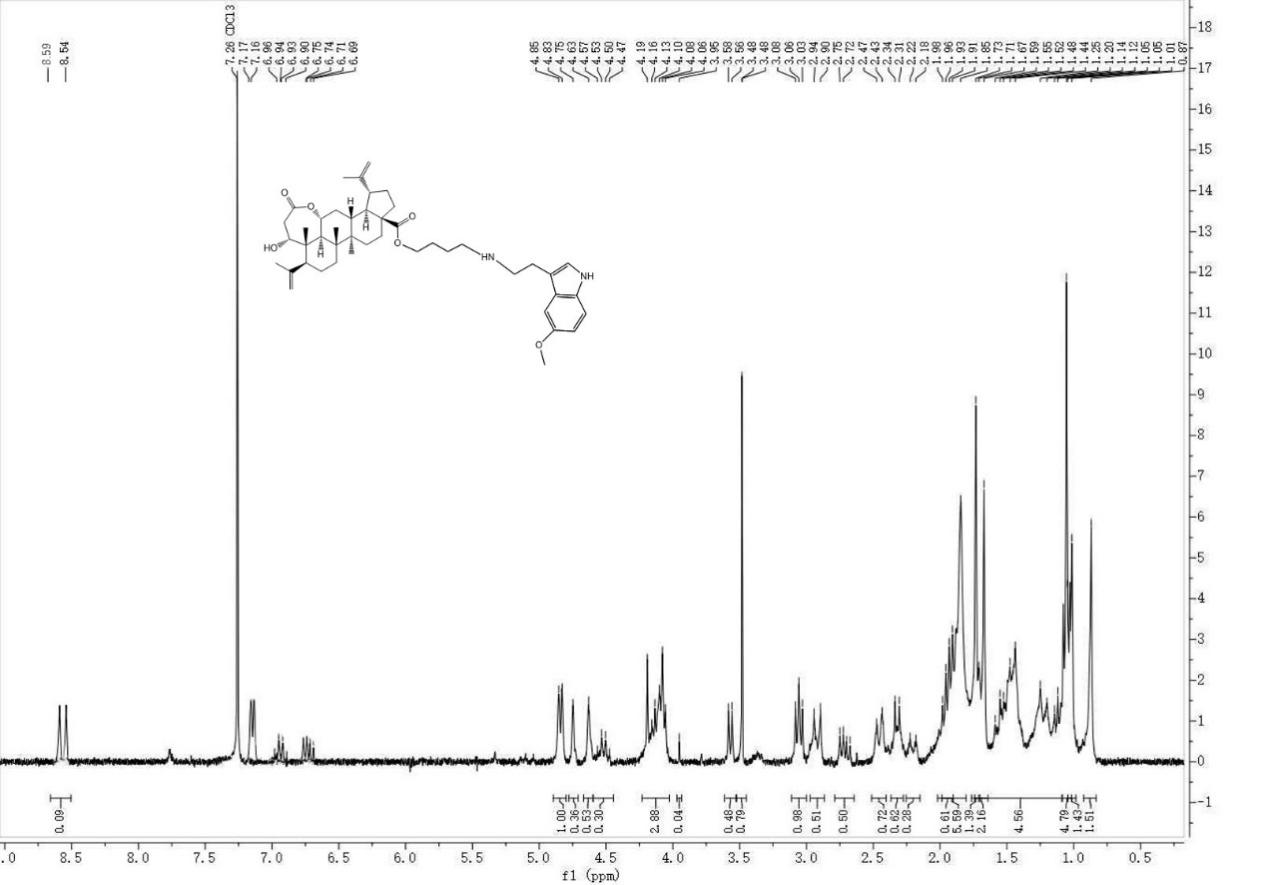


Figure S9. *HPLC tracing of compound* ***26****.*


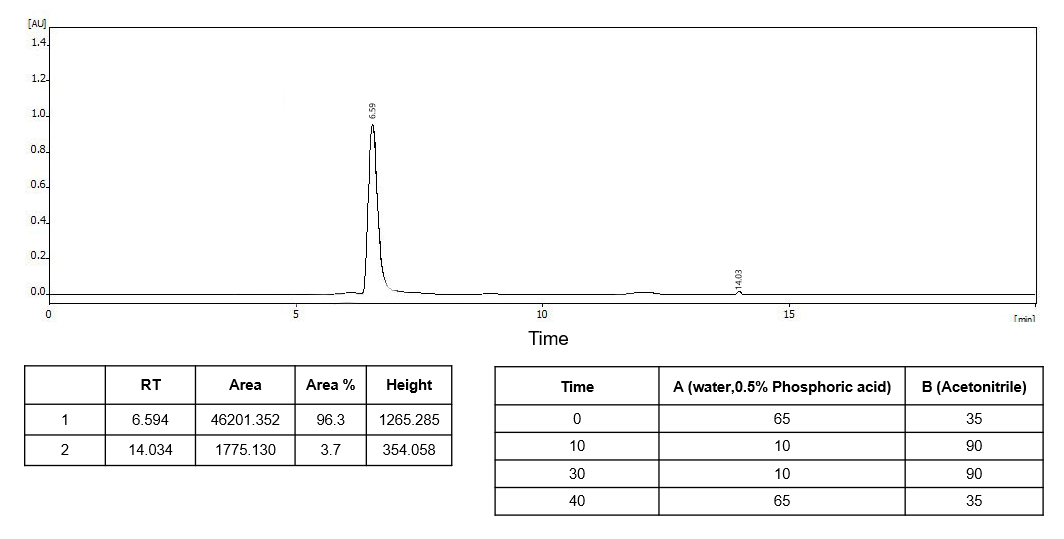


Figures S10. *^13^C and ^1^H NMR of compound* ***26****.*


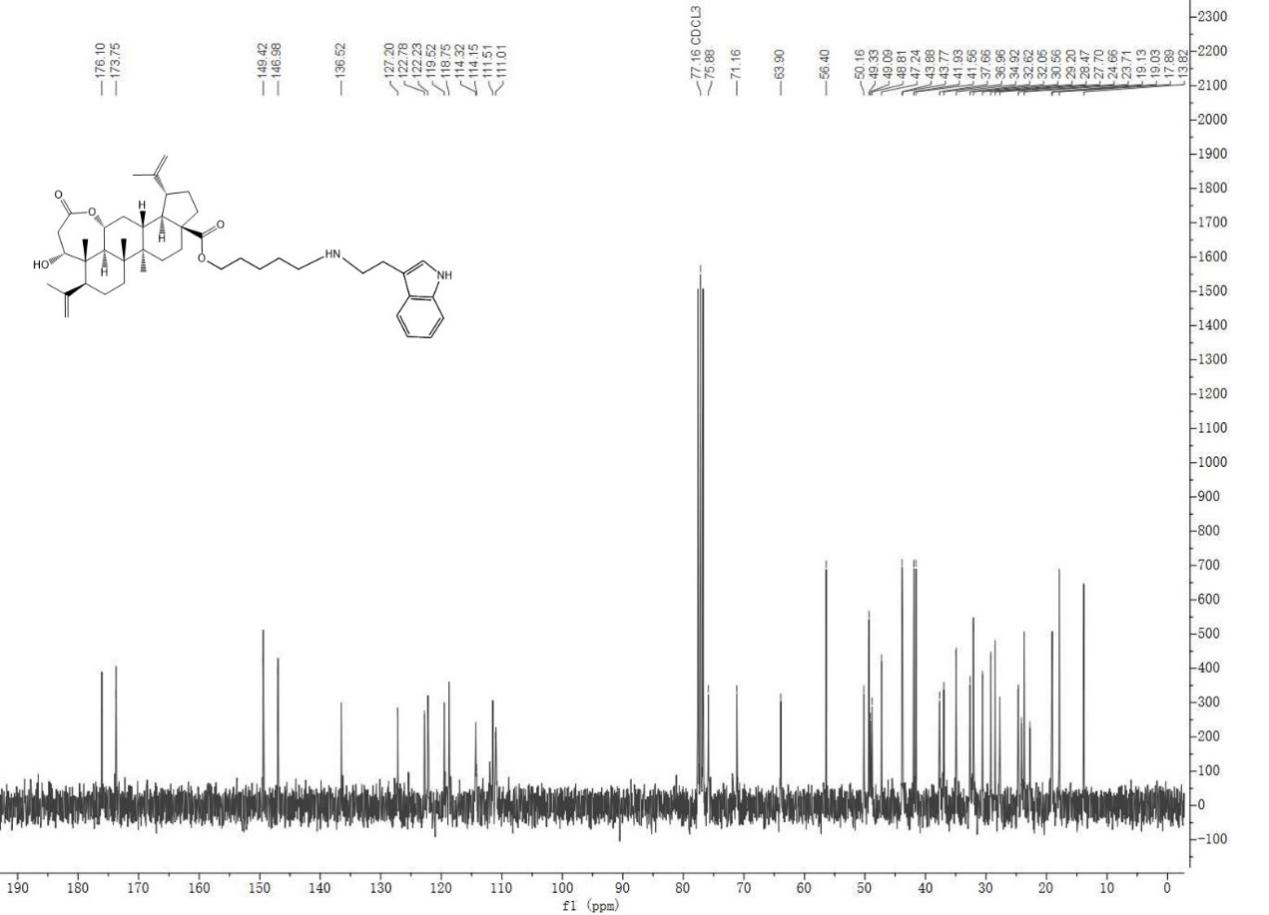


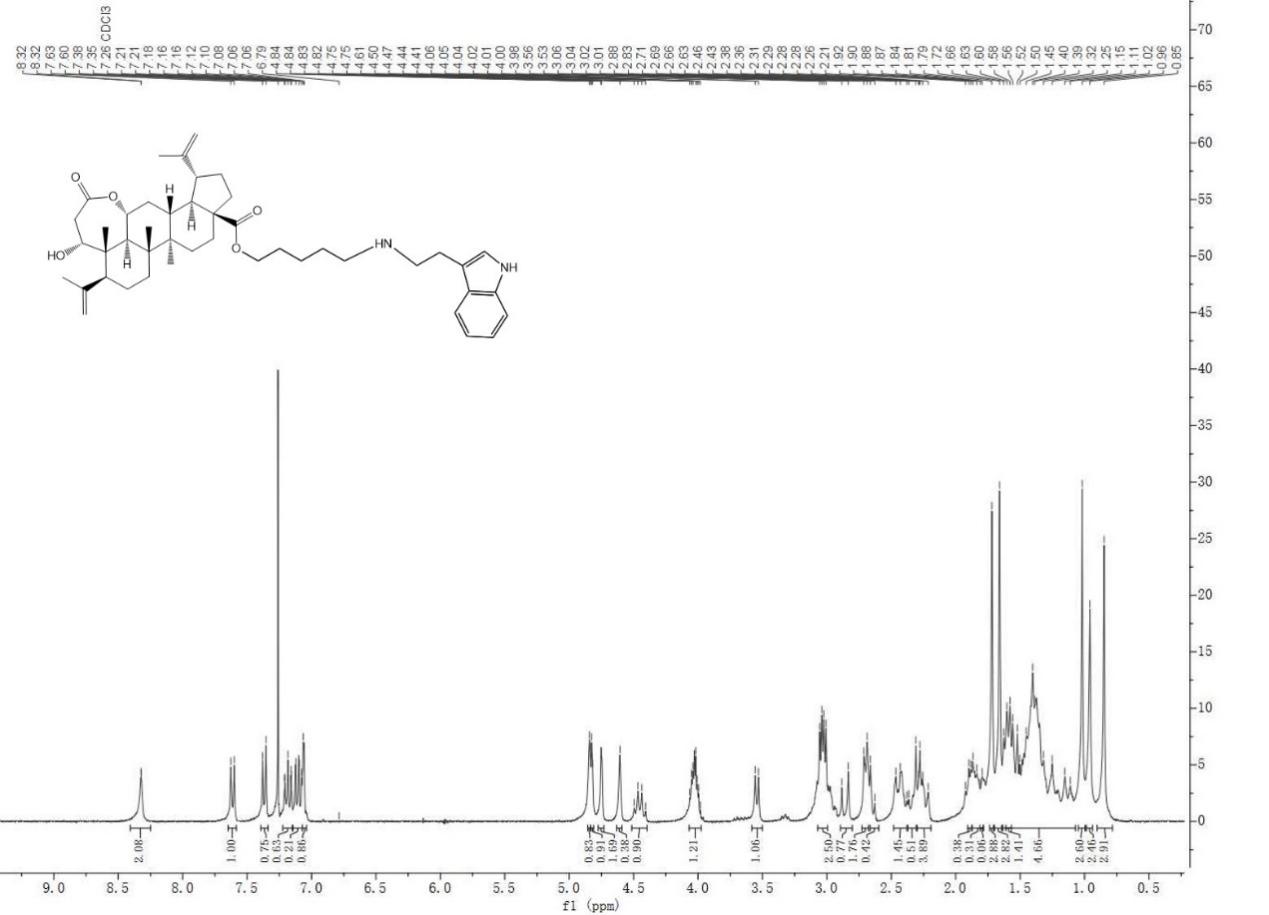


Figure S11. *HPLC tracing of compound* ***27.***


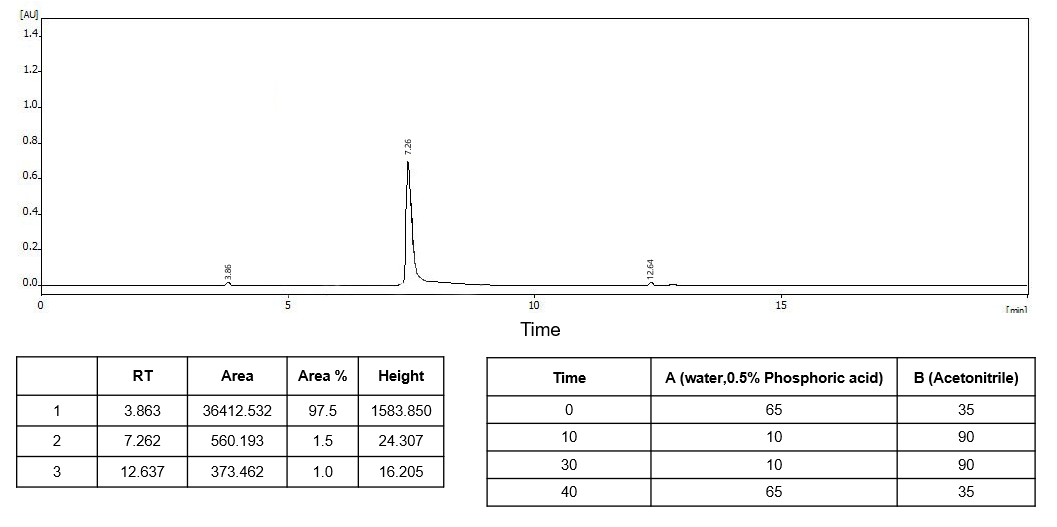


Figures S12. *^13^C and ^1^H NMR of compound* ***27****.*


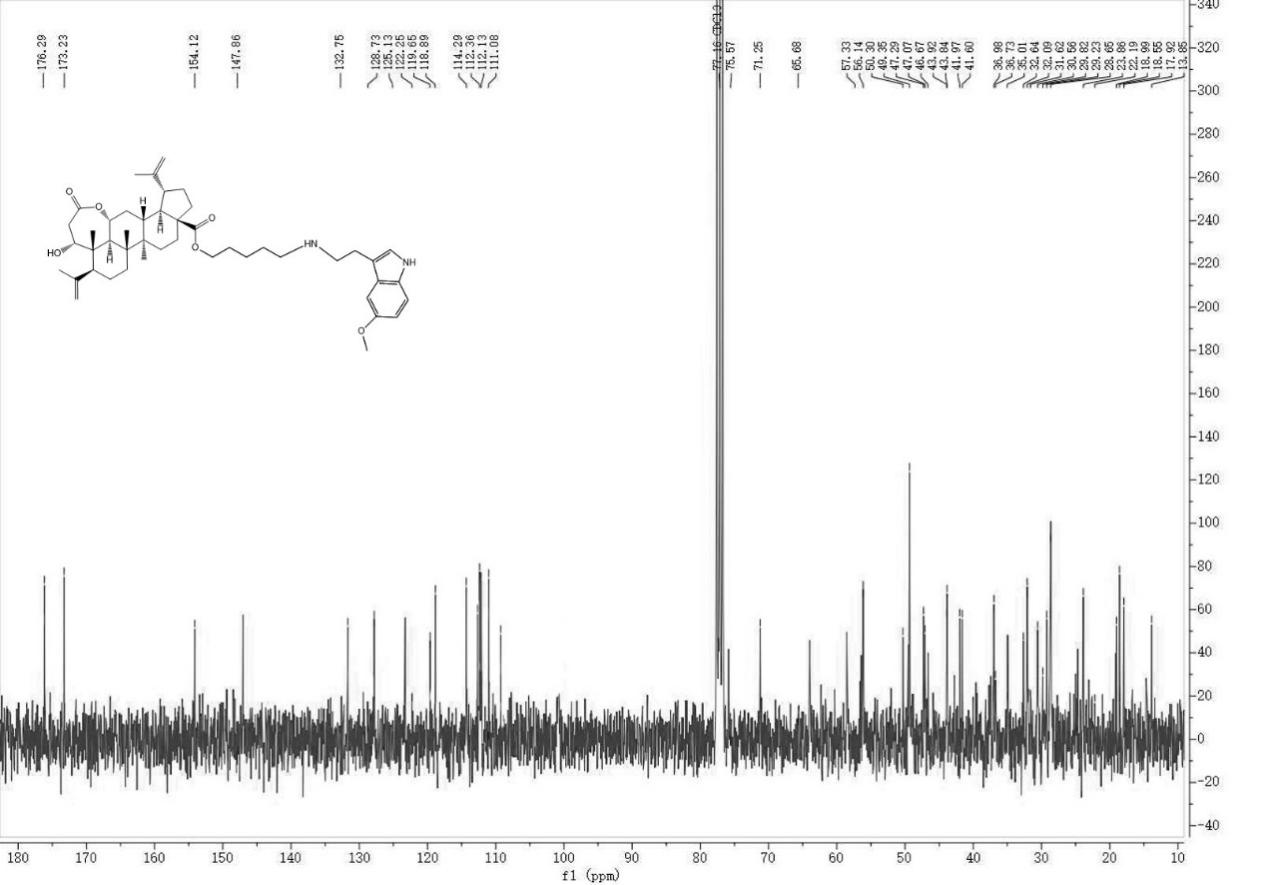


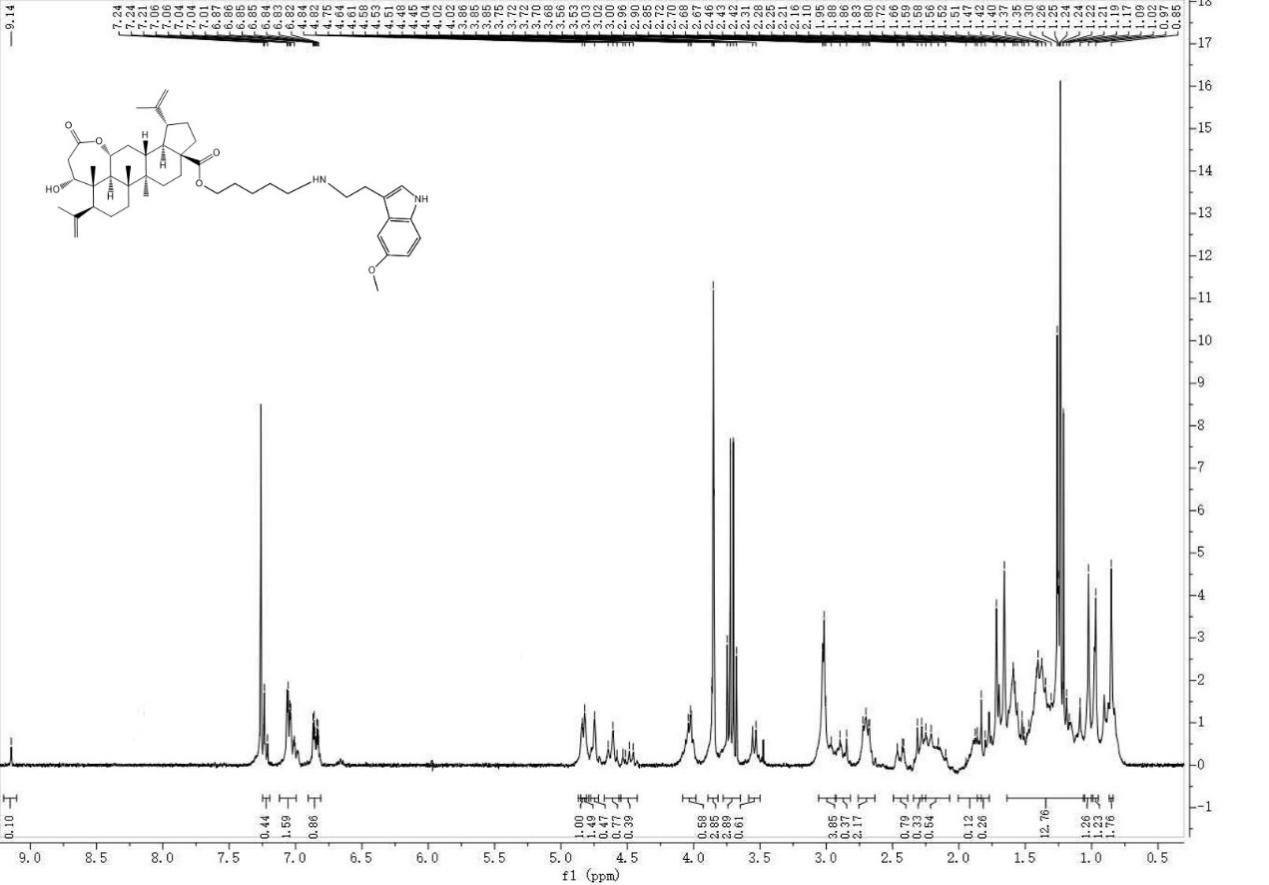


Figure S13. *HPLC tracing of compound* ***35****.*


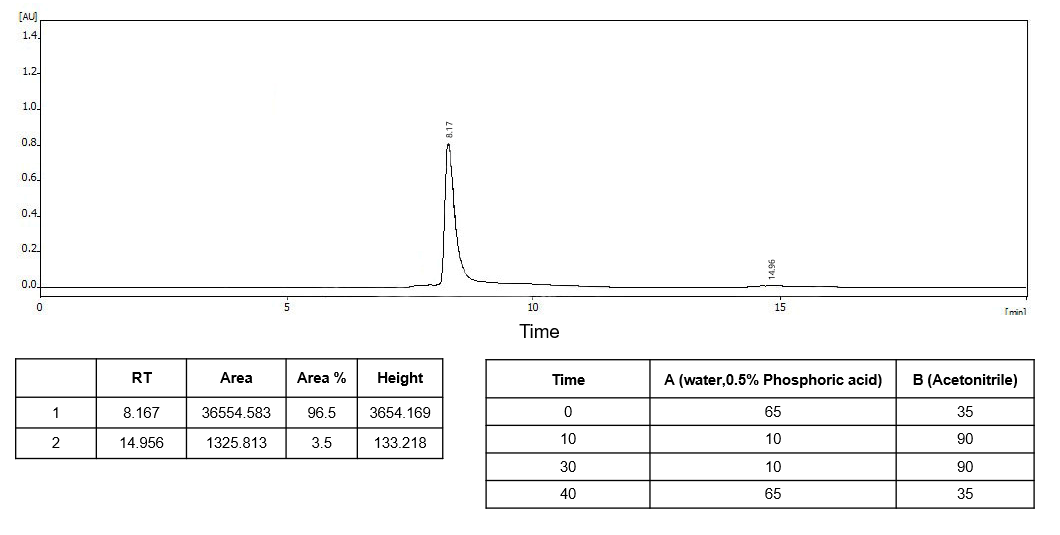


Figures S14. *^13^C and ^1^H NMR of compound* ***35****.*


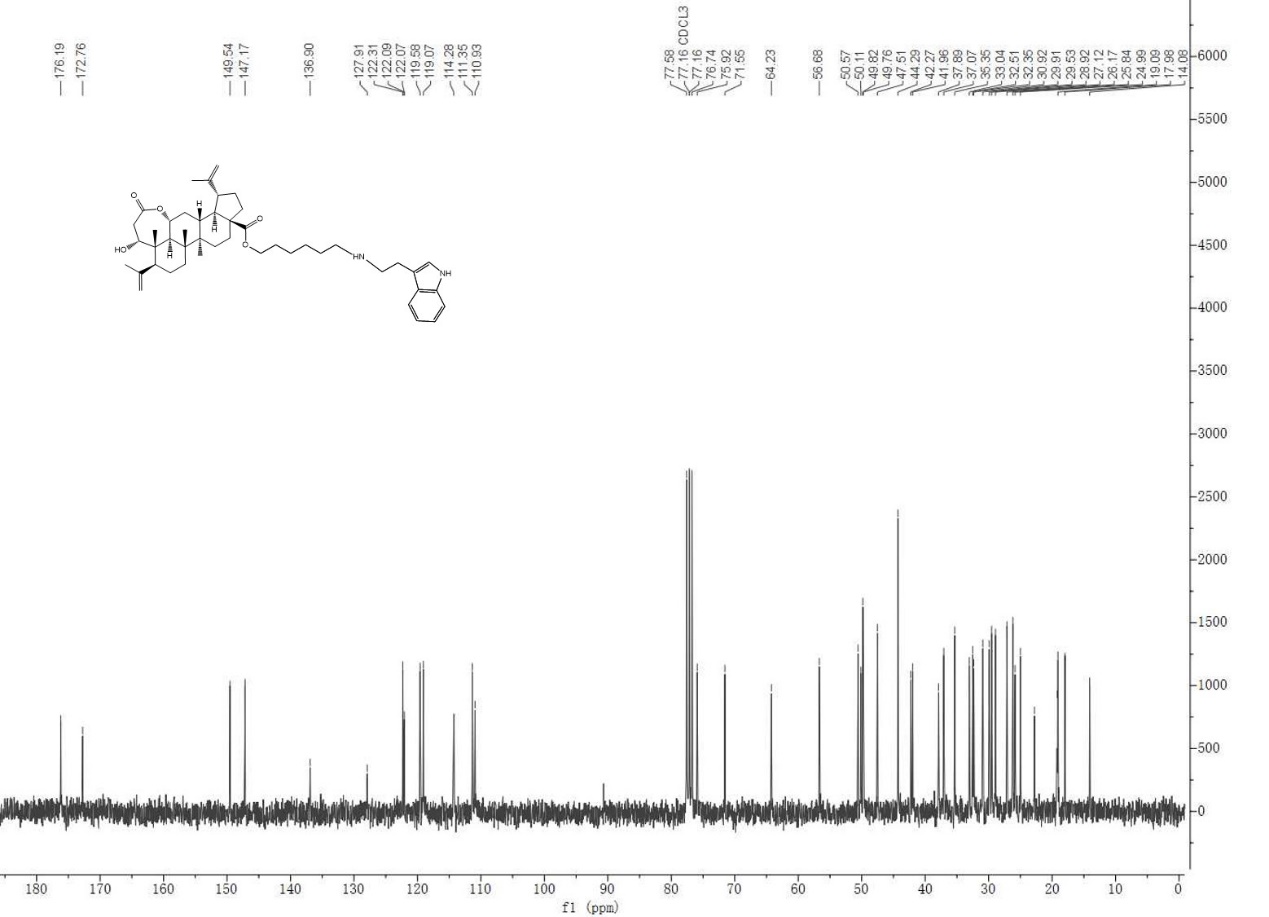


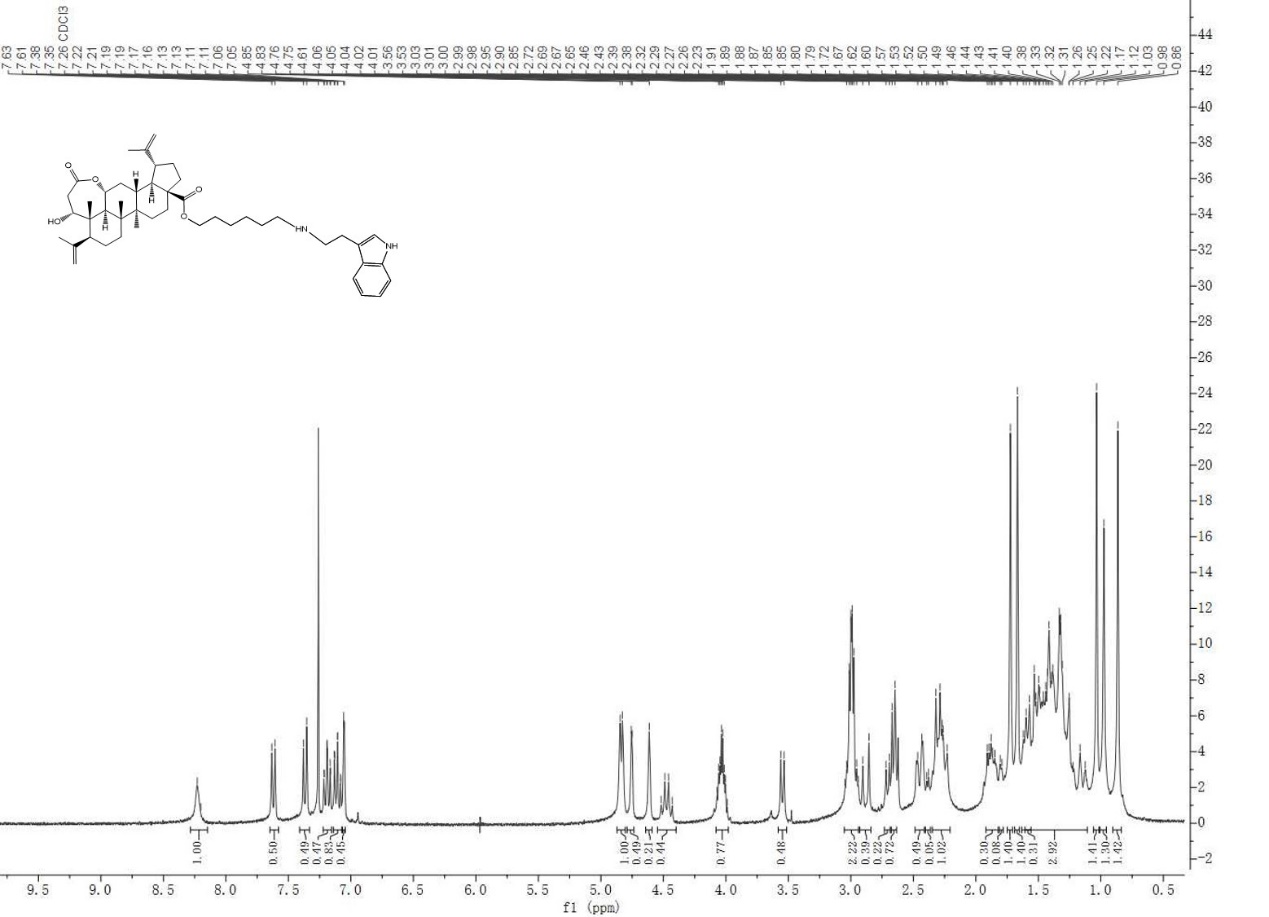


Figure S15. *HPLC tracing of compound* ***36****.*


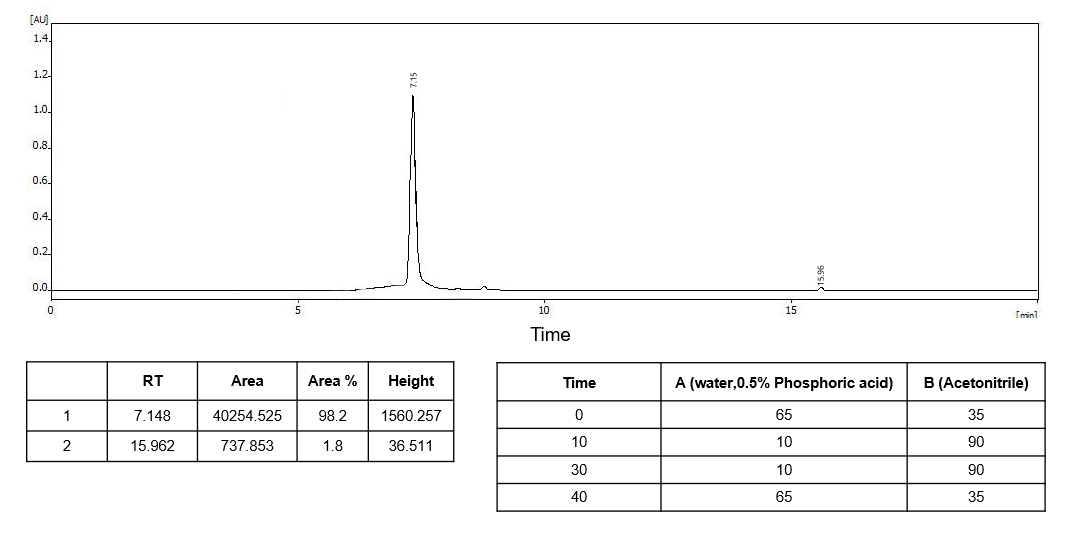


Figures S16. *^13^C and ^1^H NMR of compound* ***36****.*


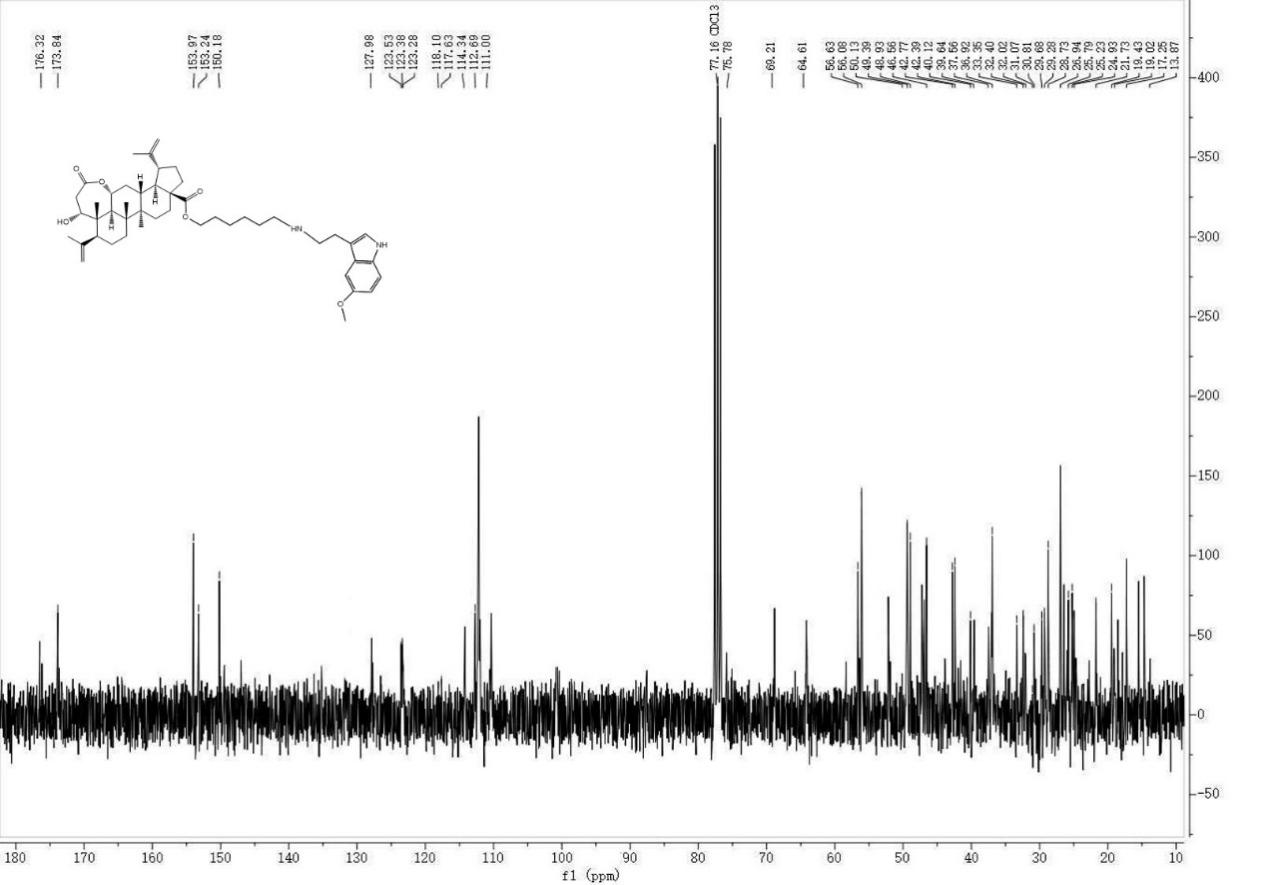

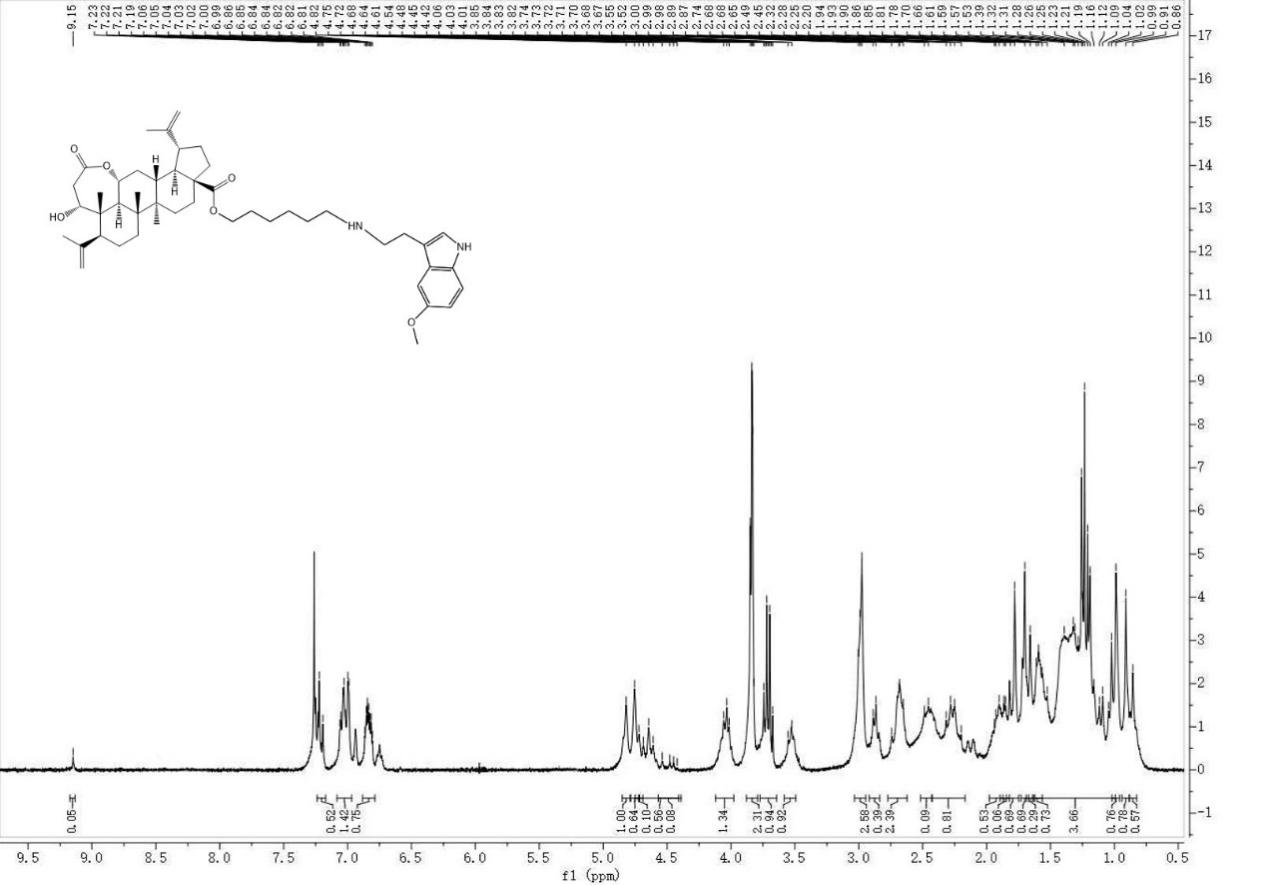


Figure S17. *HPLC tracing of compound* ***44****.*


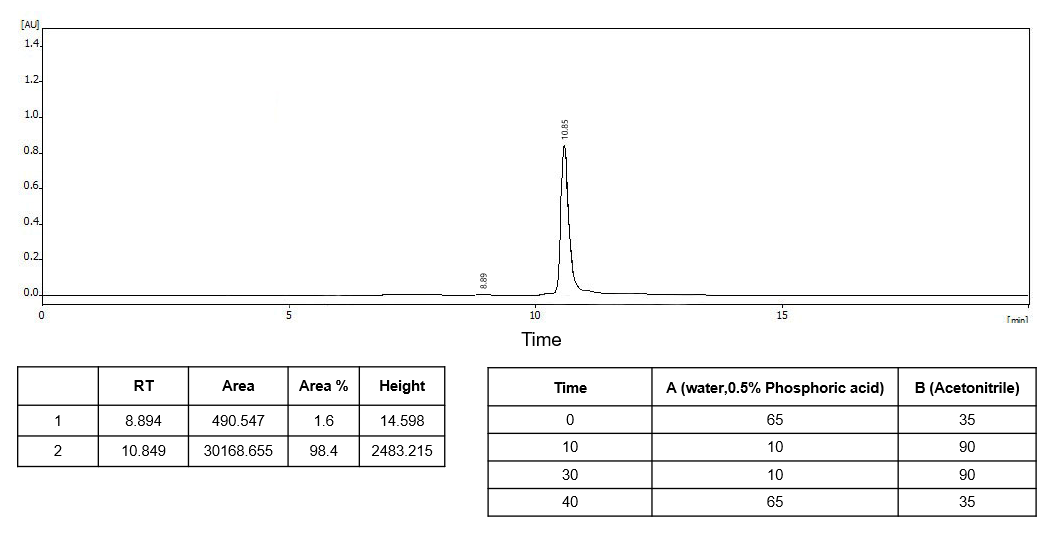


Figures S18. *^13^C and ^1^H NMR of compound* ***44****.*


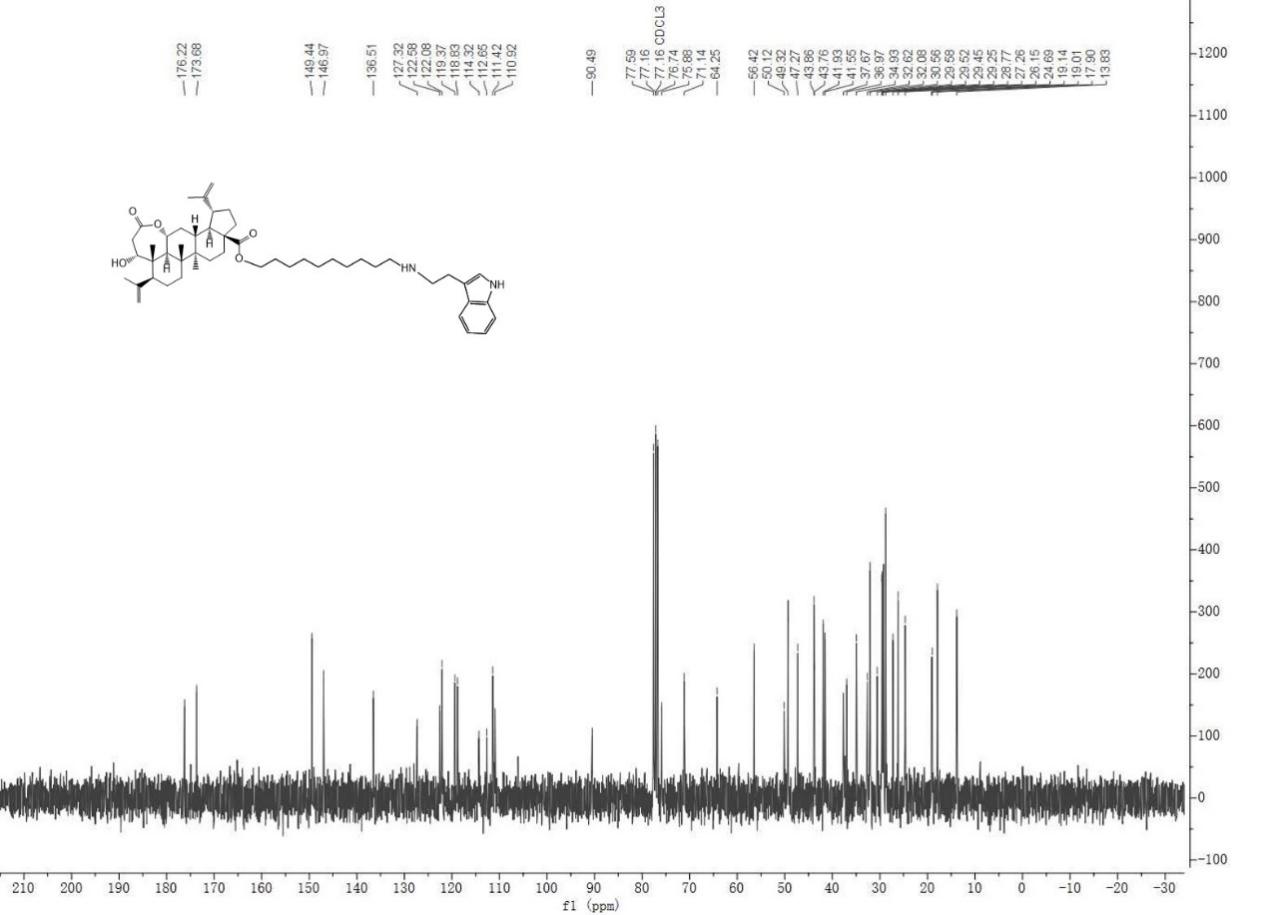

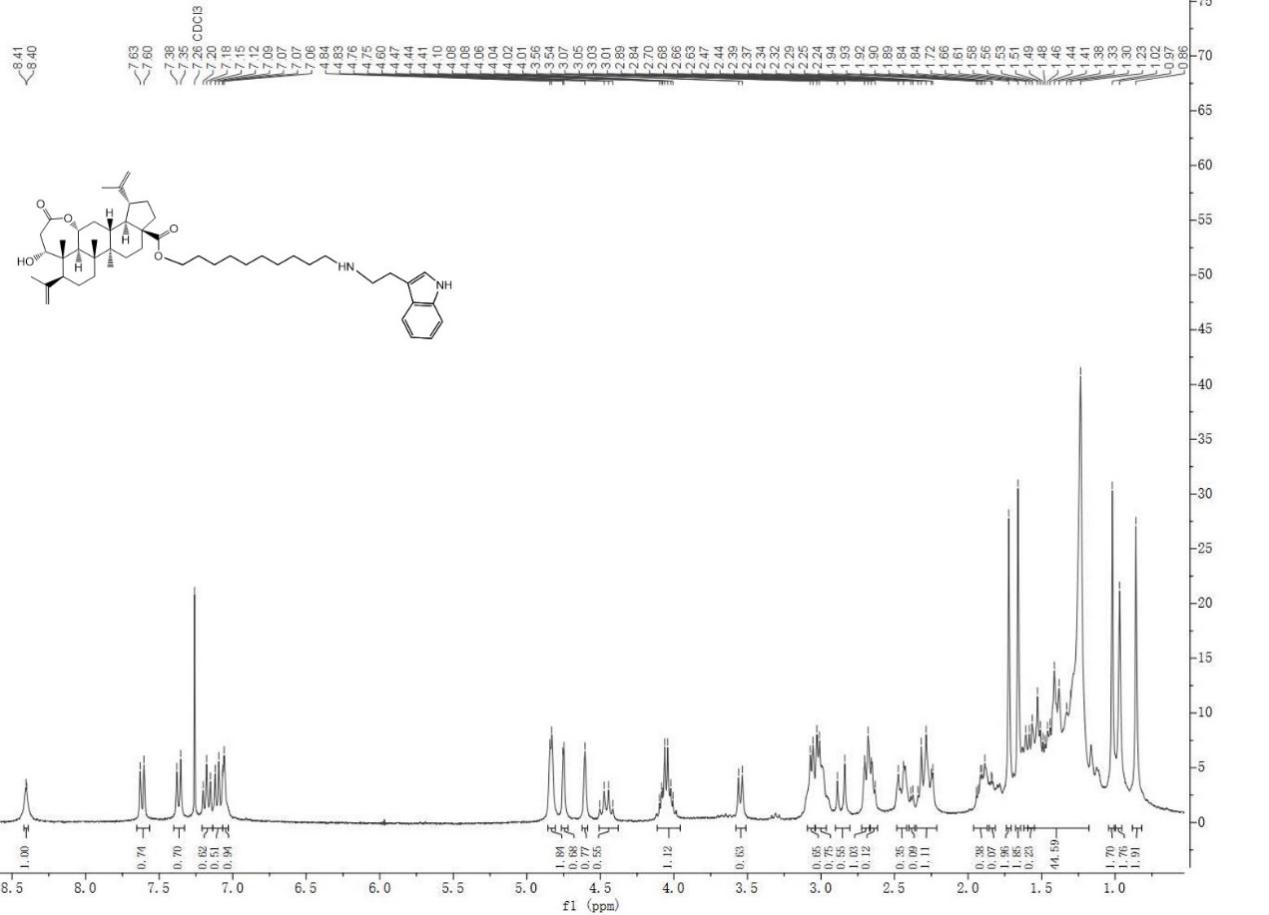


Figure S19. *HPLC tracing of compound* ***45****.*


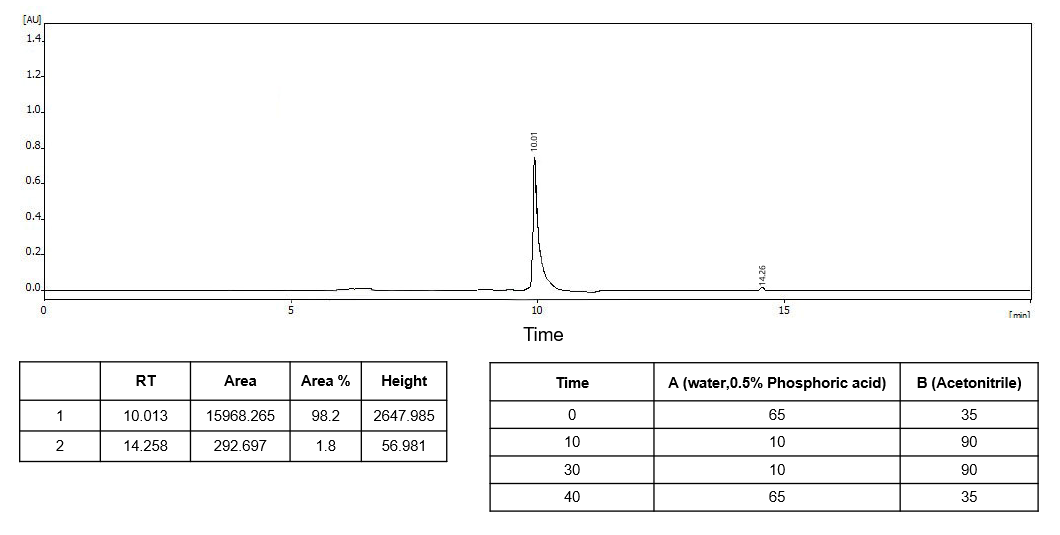


Figures S20. *^13^C and ^1^H NMR of compound* ***45****.*


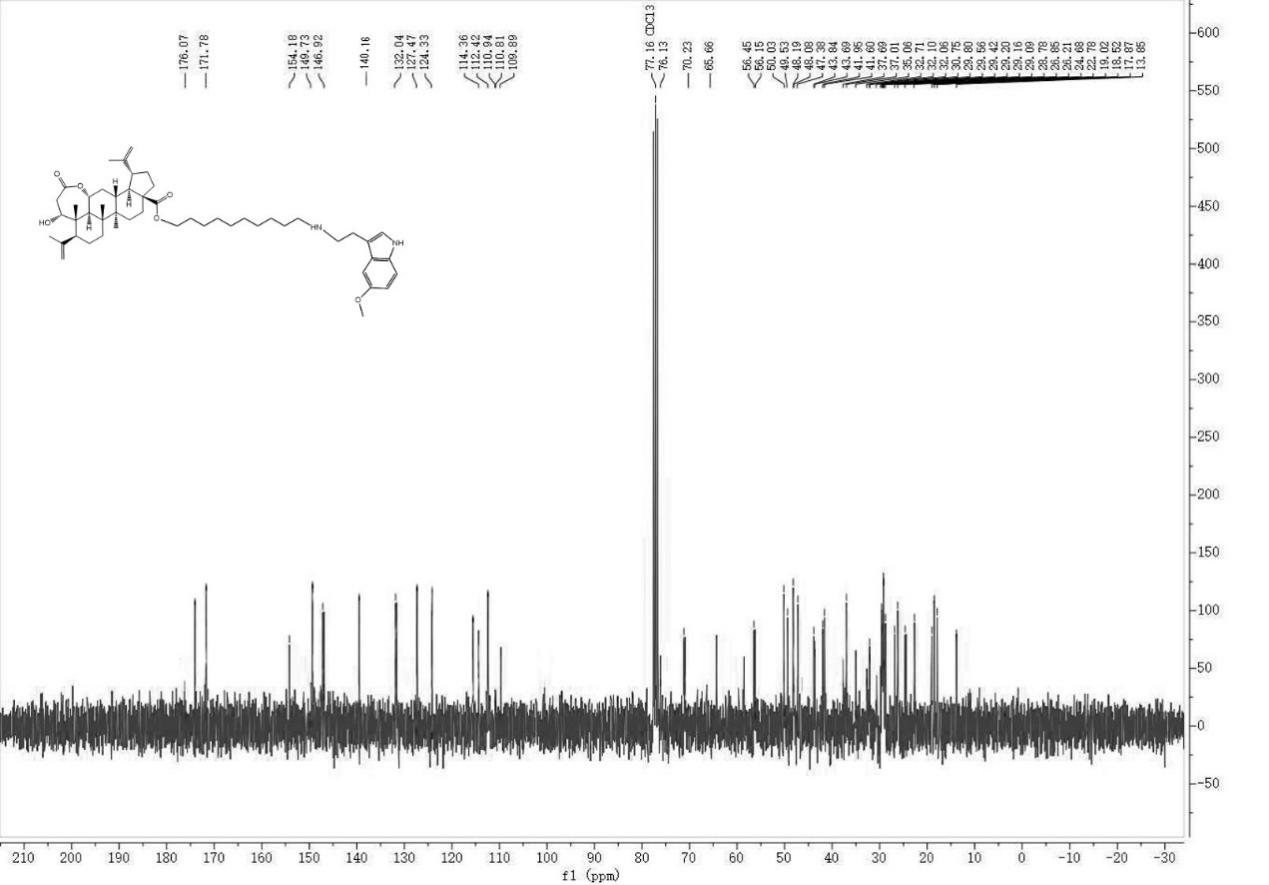

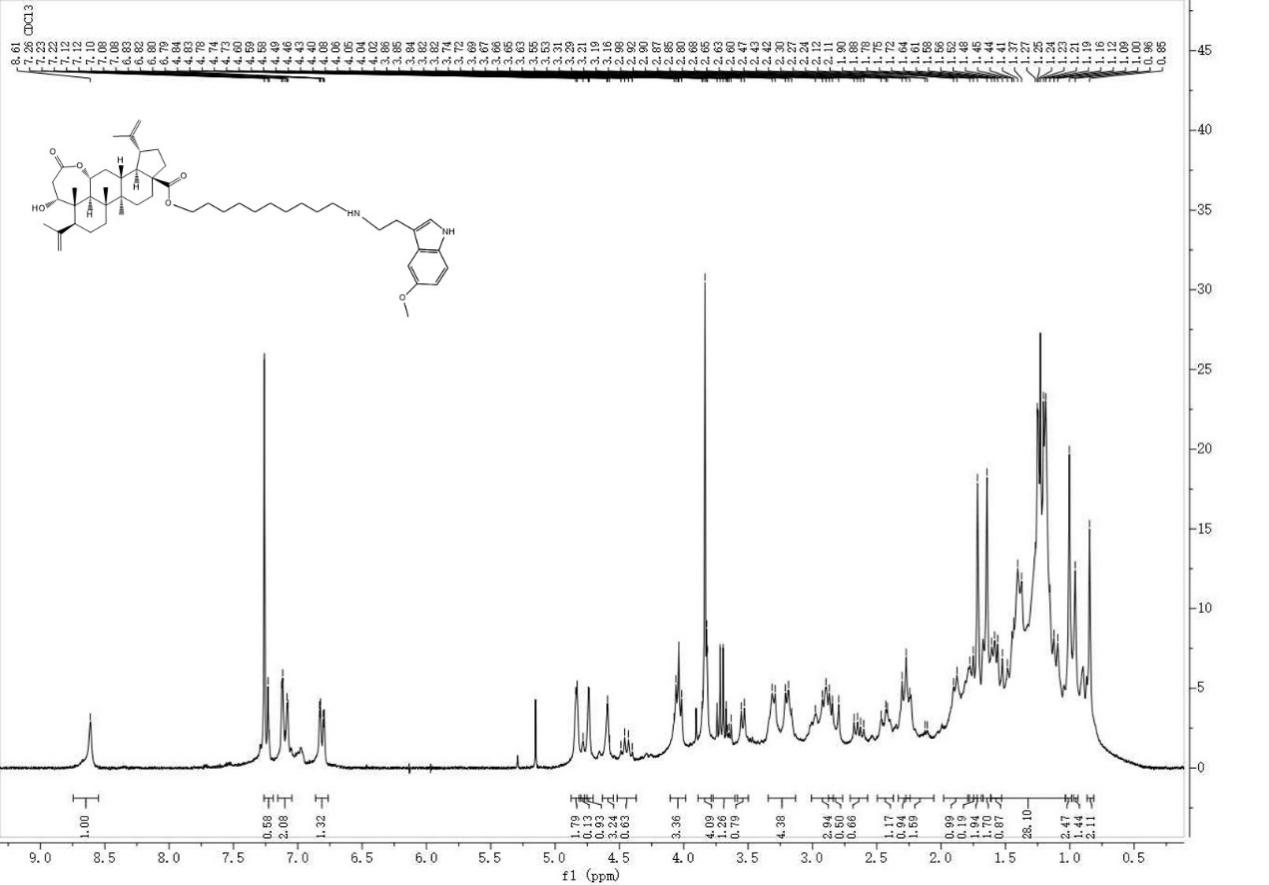


Figure S21. *HPLC tracing of compound* ***53****.*


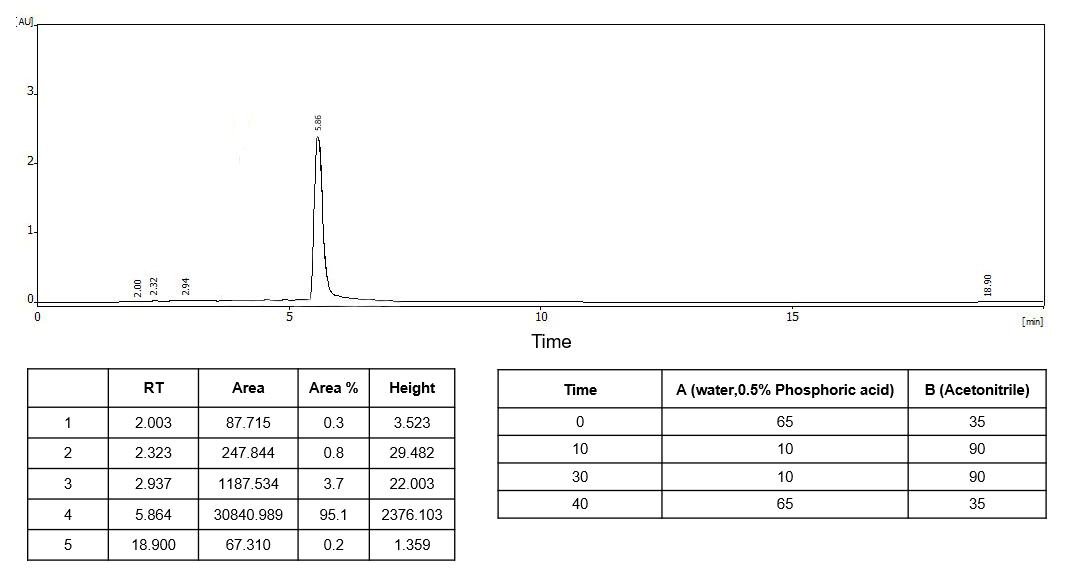


Figures S22. *^13^C and ^1^H NMR of compound* ***53****.*


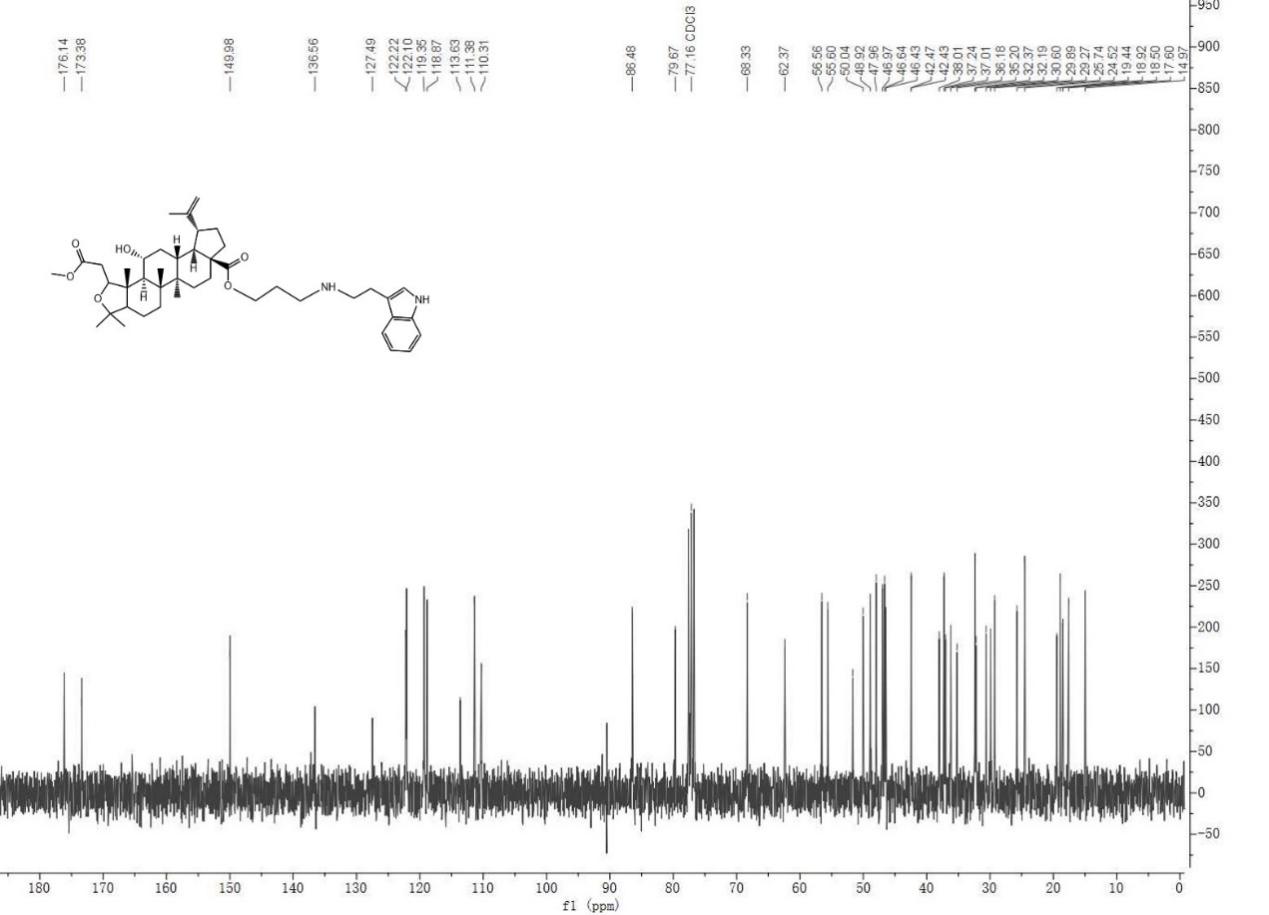

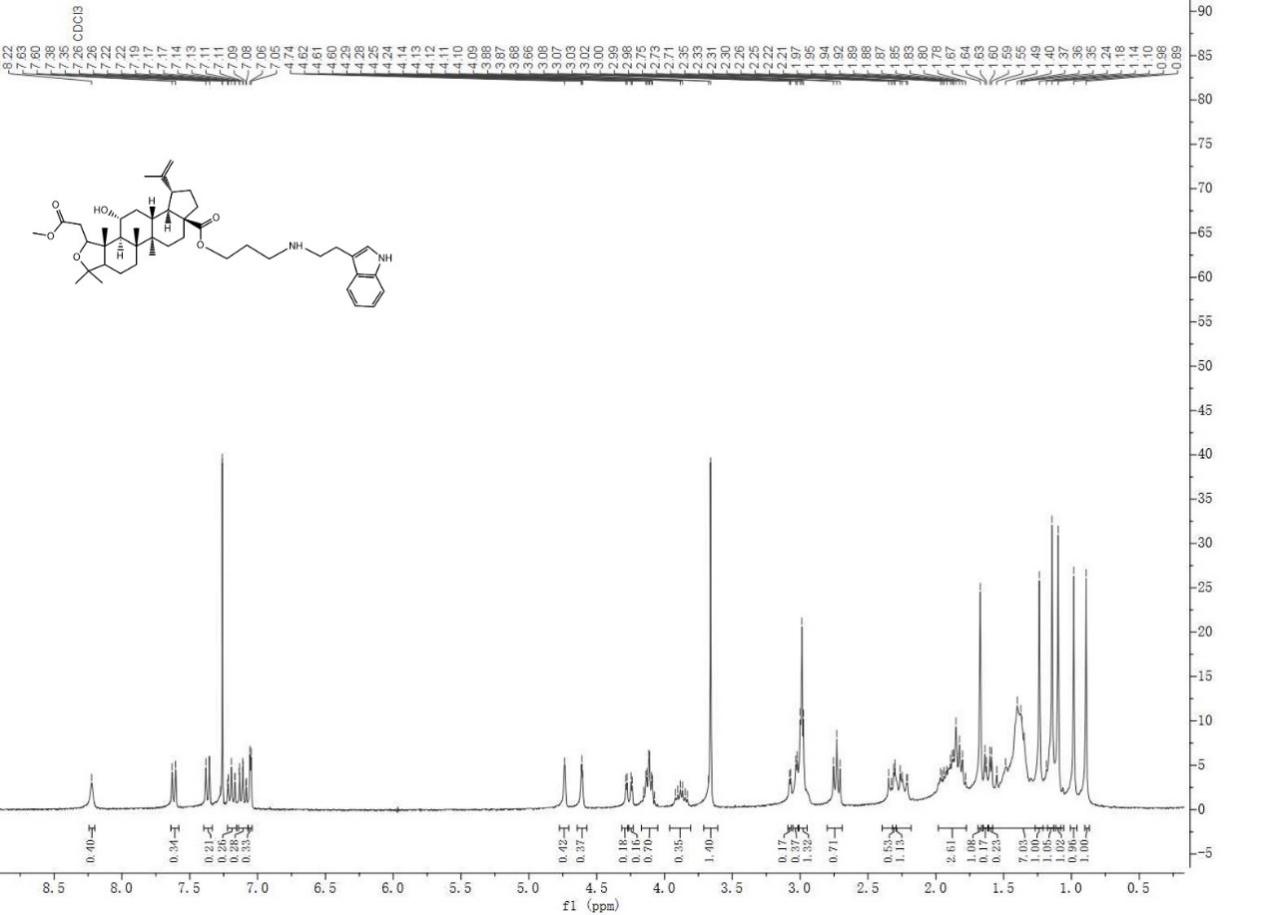


Figure S23. *HPLC tracing of compound* ***54****.*


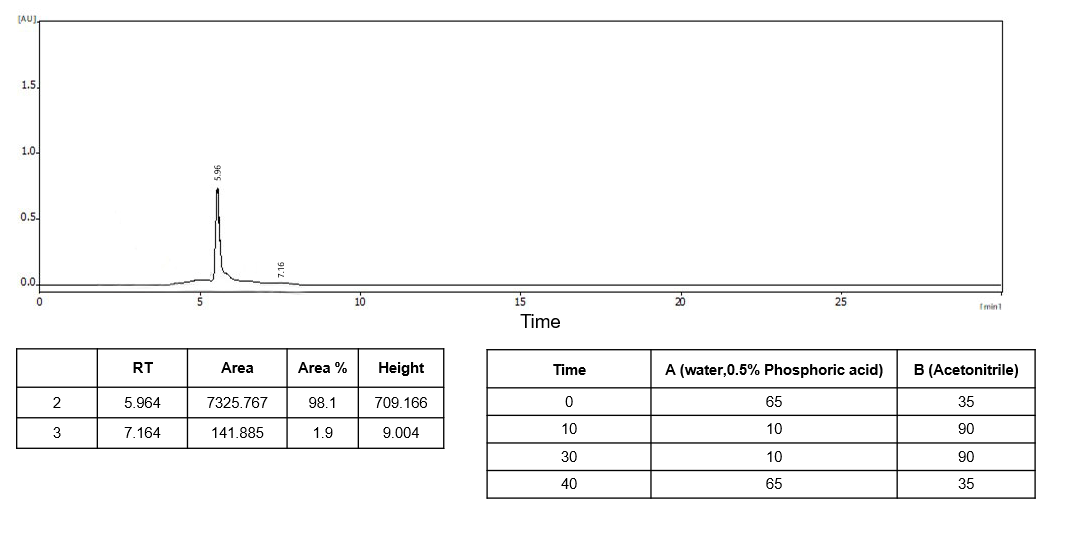


Figures S24. *^13^C and ^1^H NMR of compound* ***54****.*


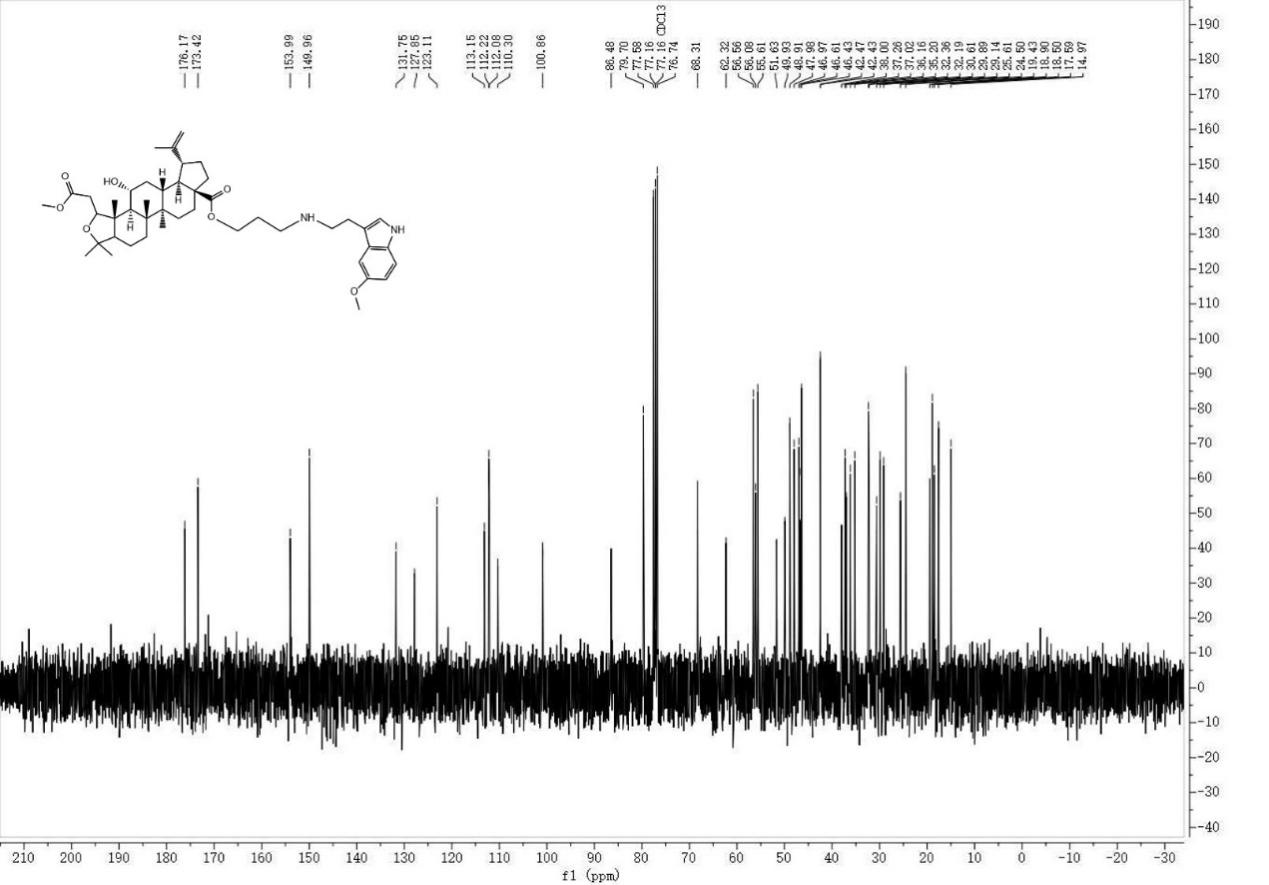

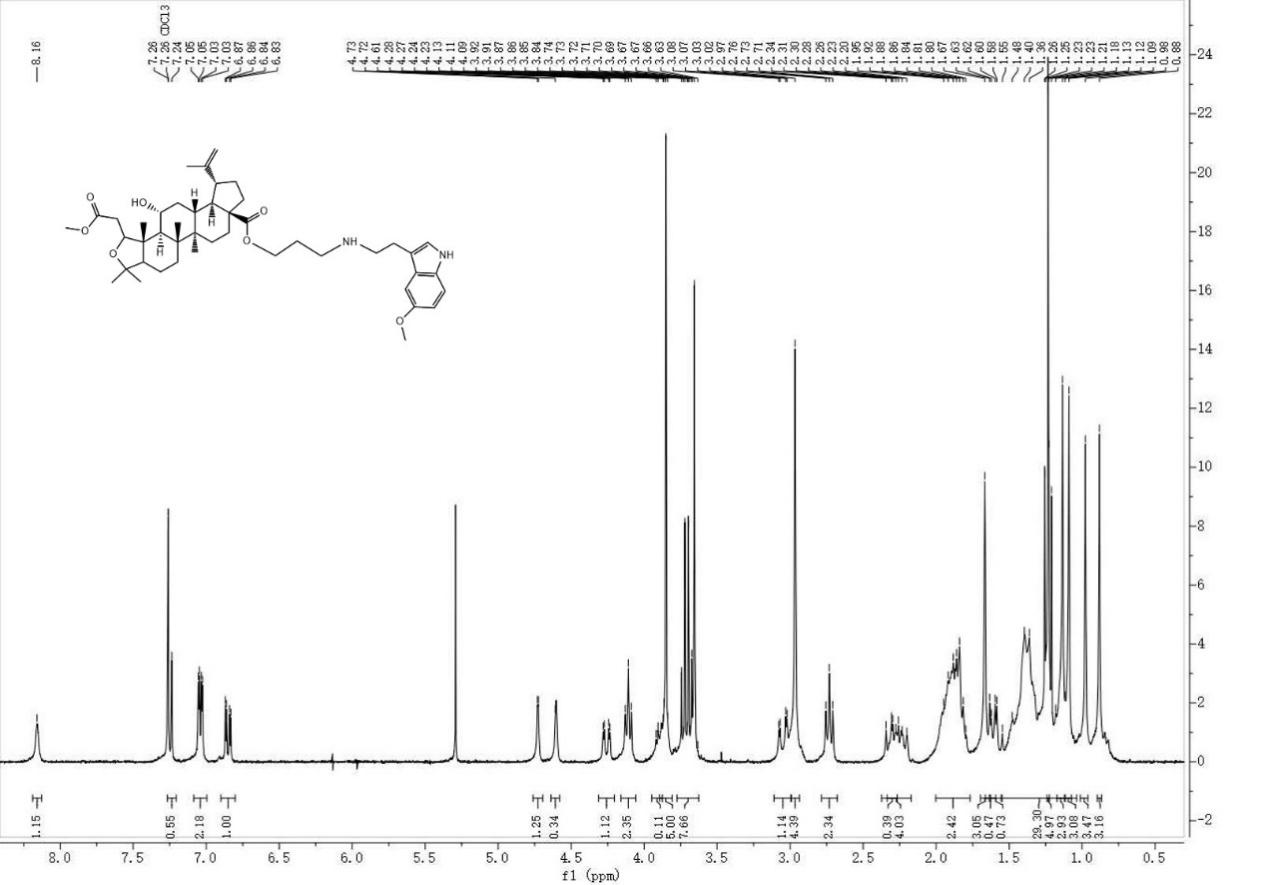


Figure S25. *HPLC tracing of compound* ***62****.*


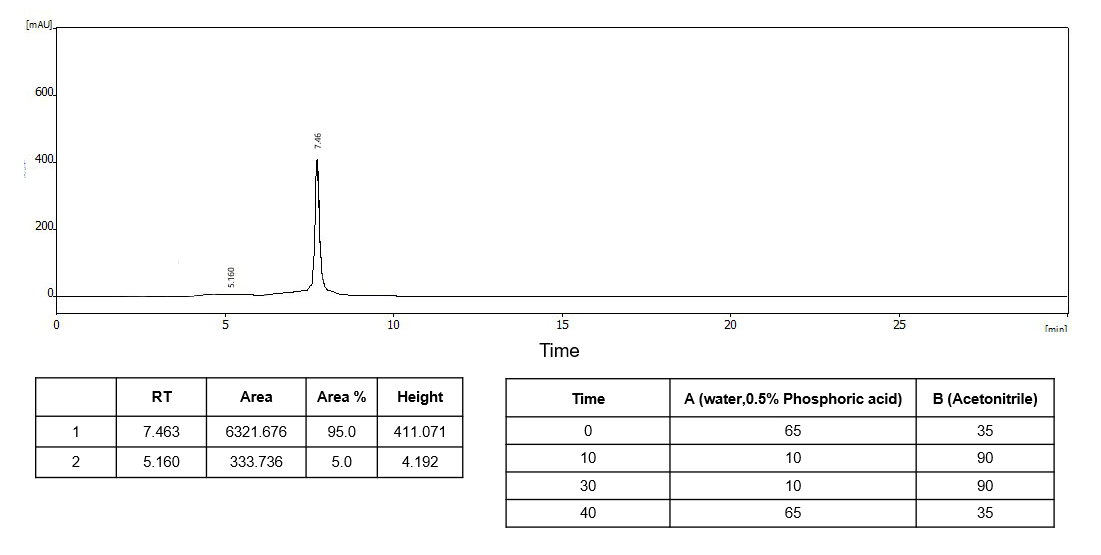


Figures S26. *^13^C and ^1^H NMR of compound* ***62****.*


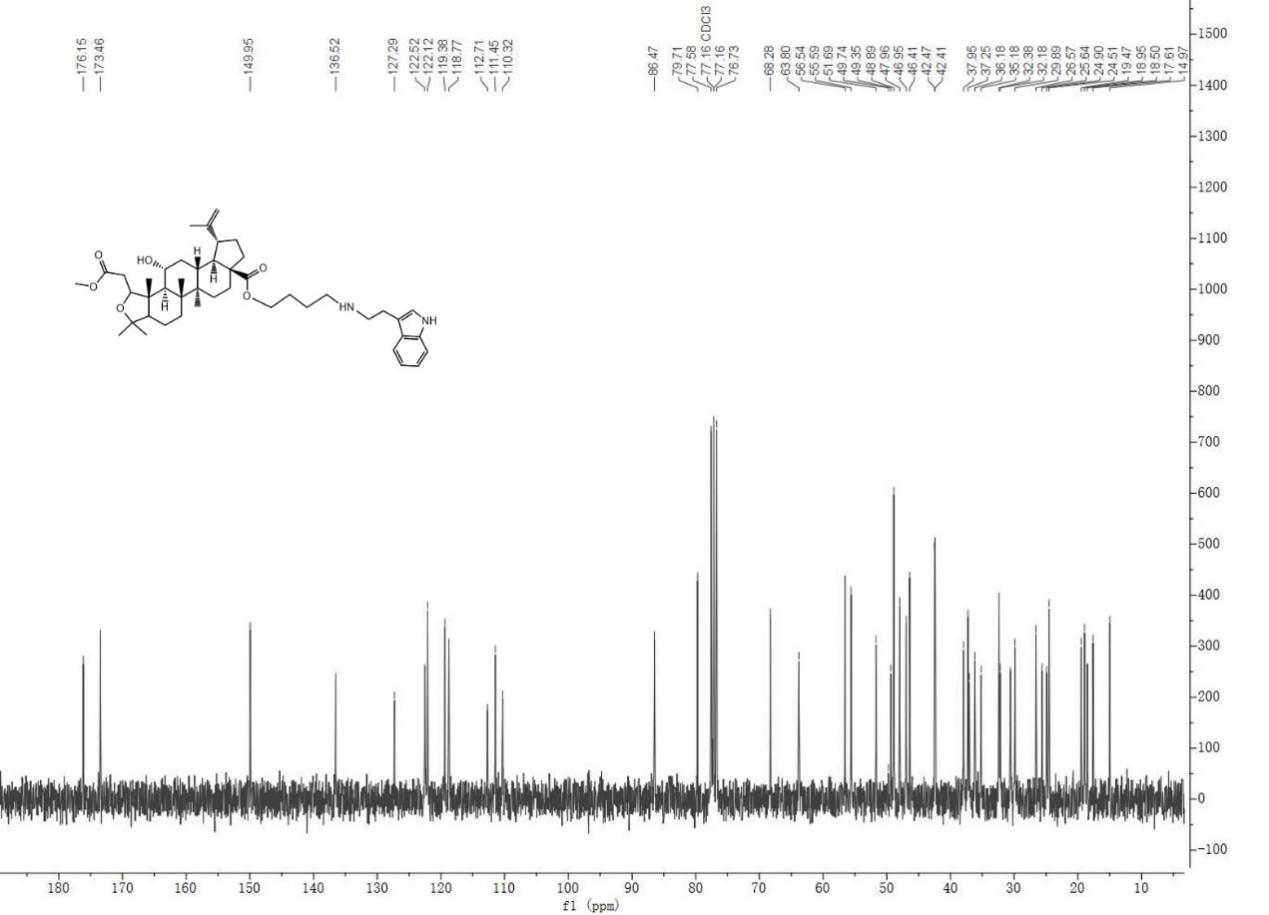

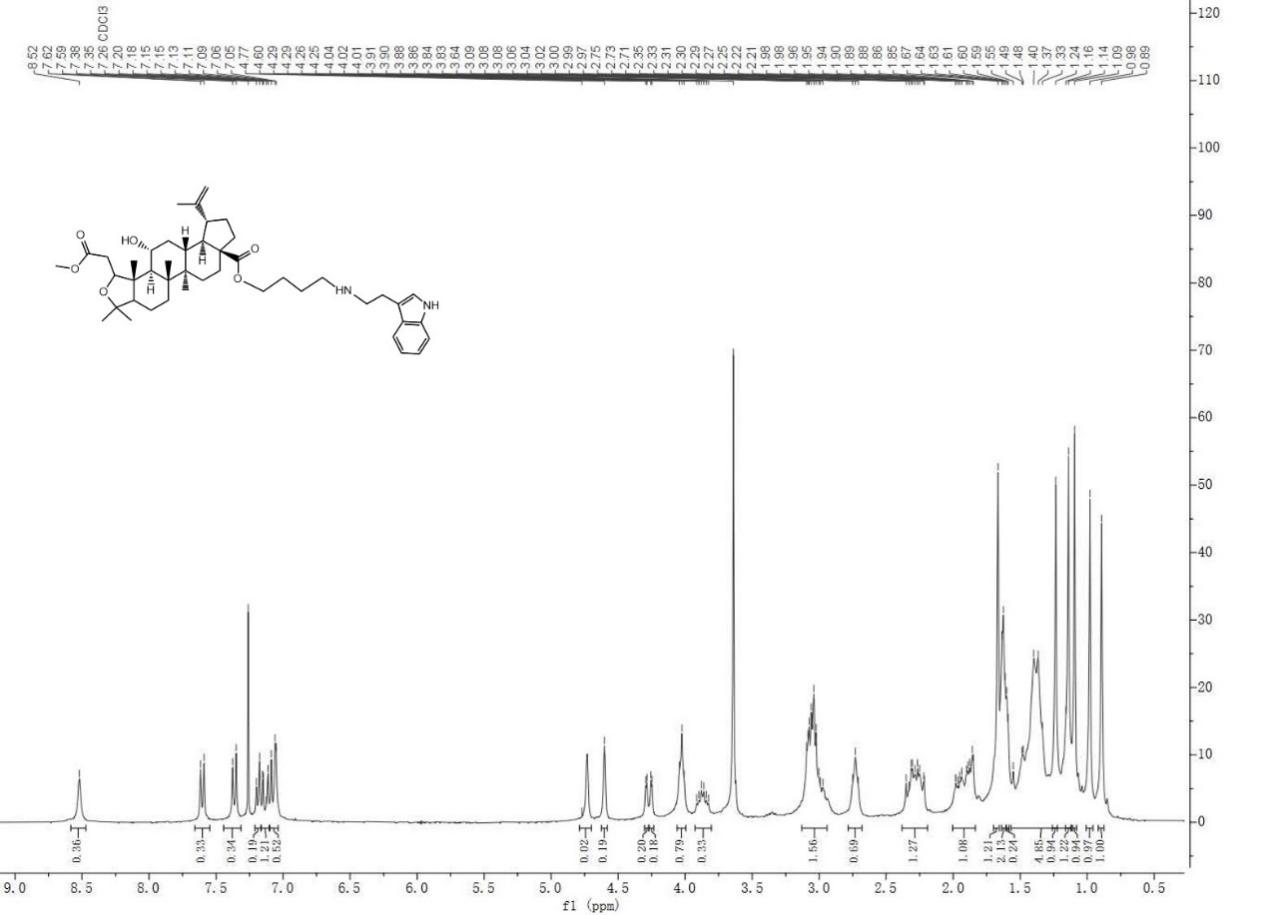


Figure S27. *HPLC tracing of compound* ***63****.*


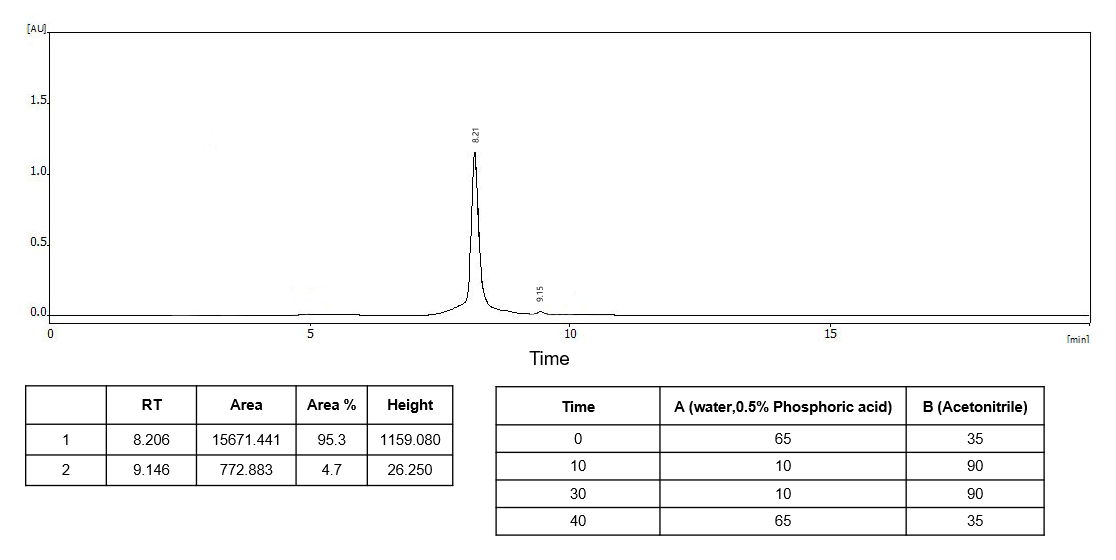


Figures S28. *^13^C and ^1^H NMR of compound* ***63****.*


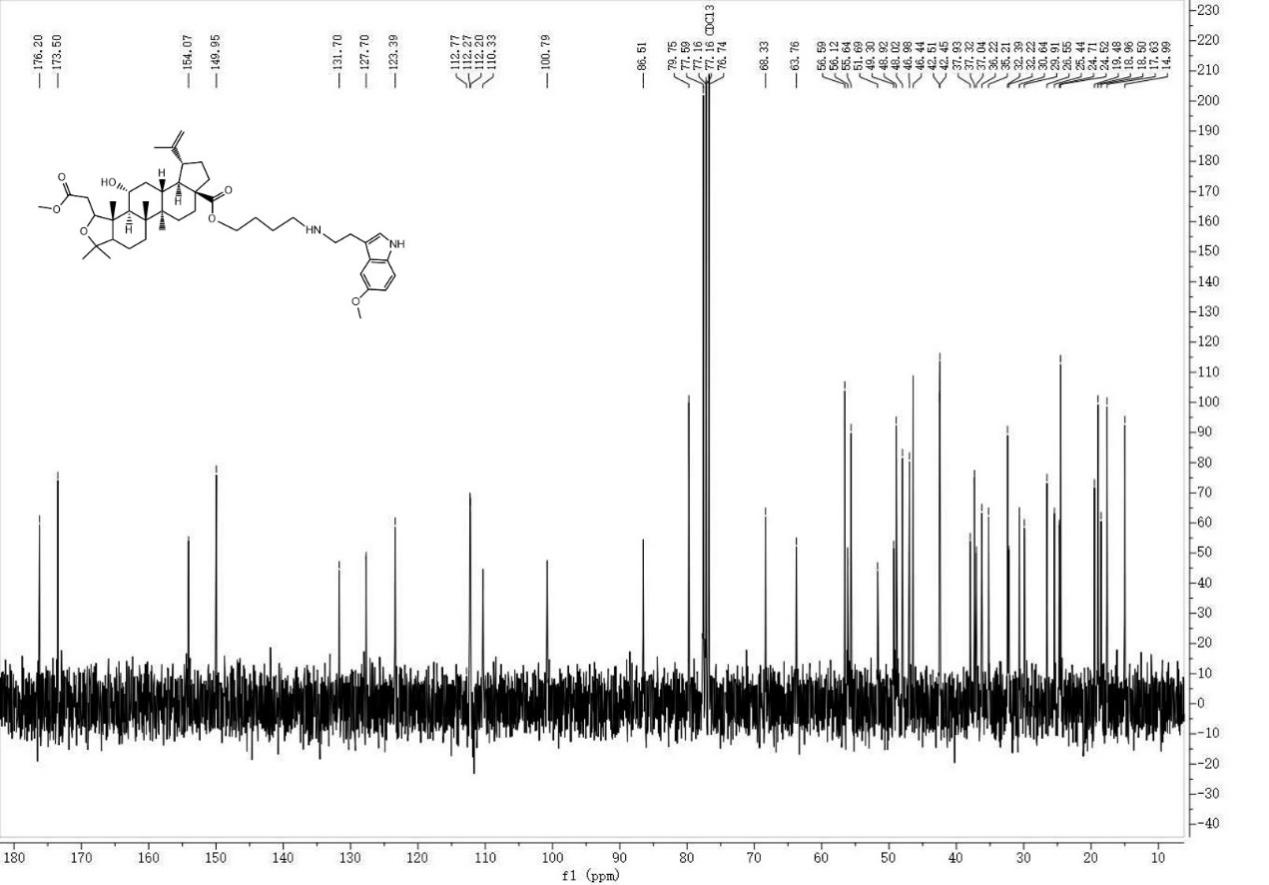

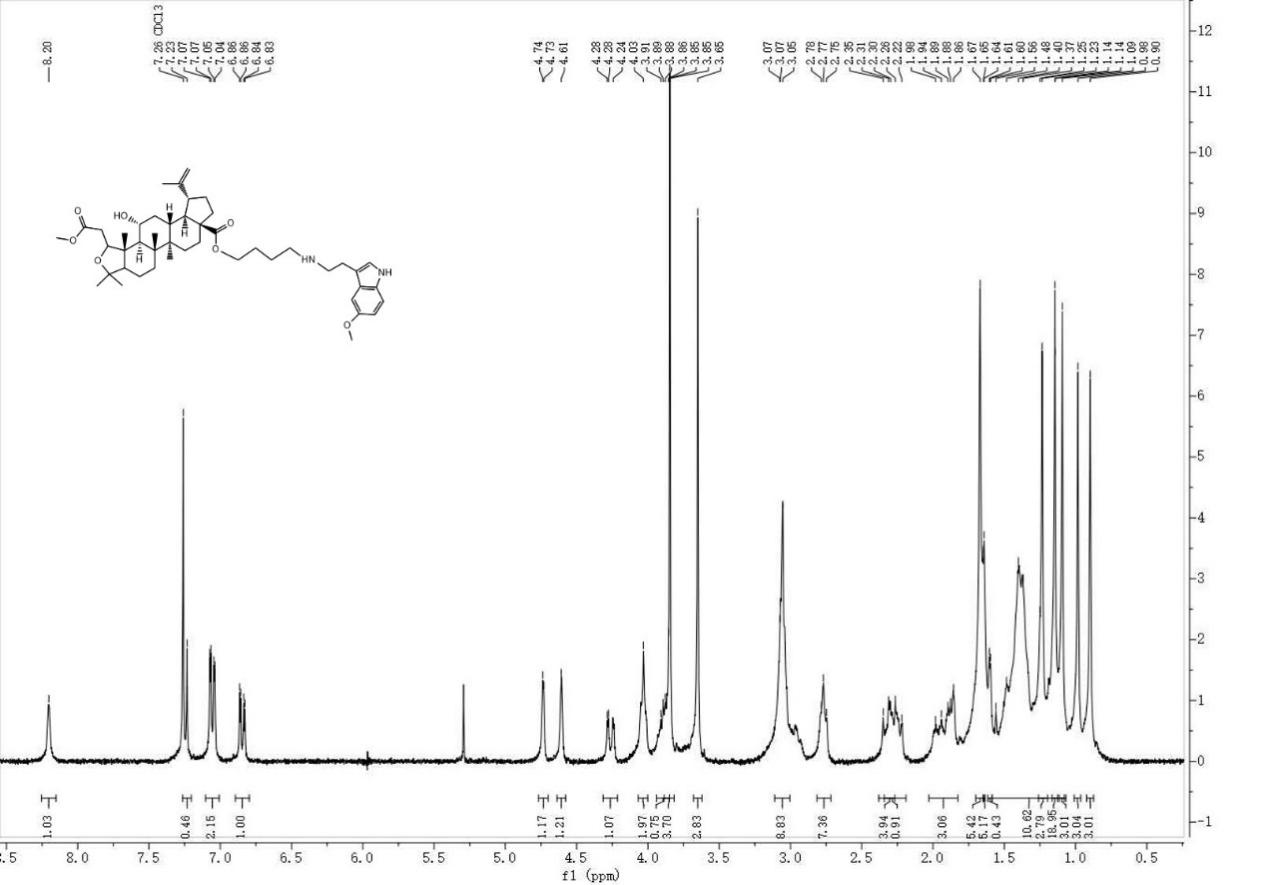


Figure S29. *HPLC tracing of compound* ***71****.*


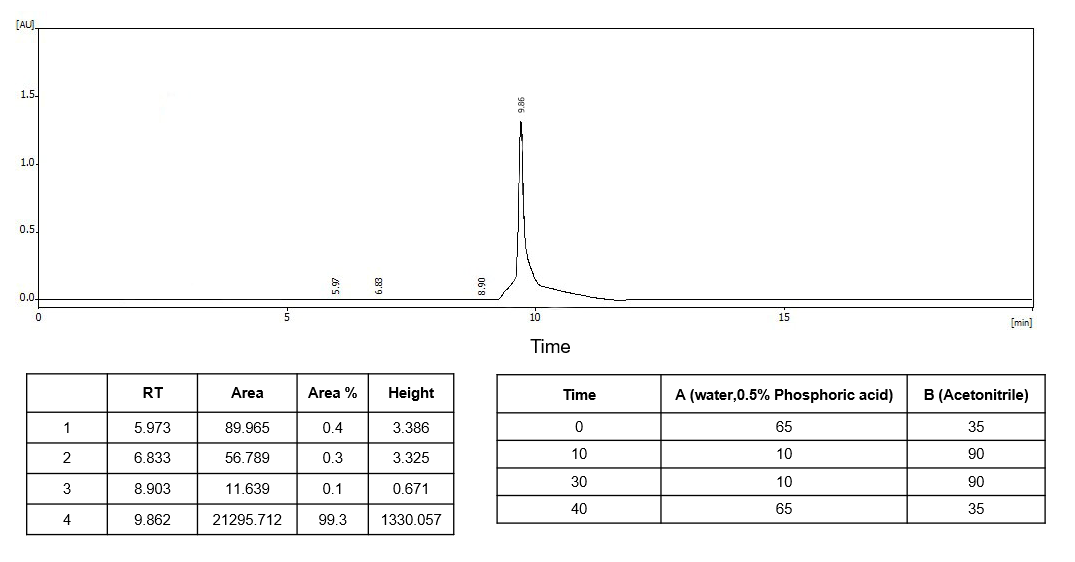


Figures S30. *^13^C and ^1^H NMR of compound* ***71****.*


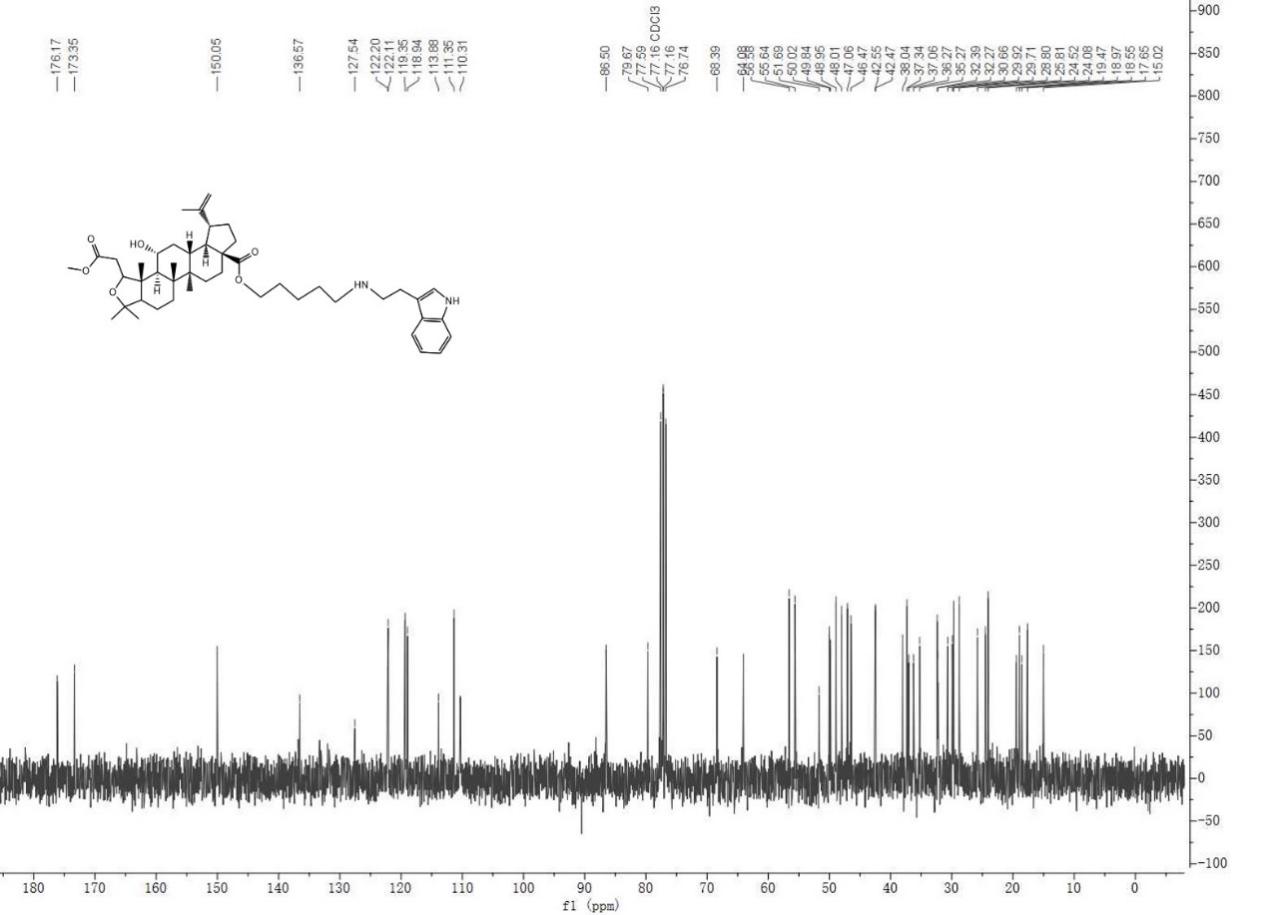

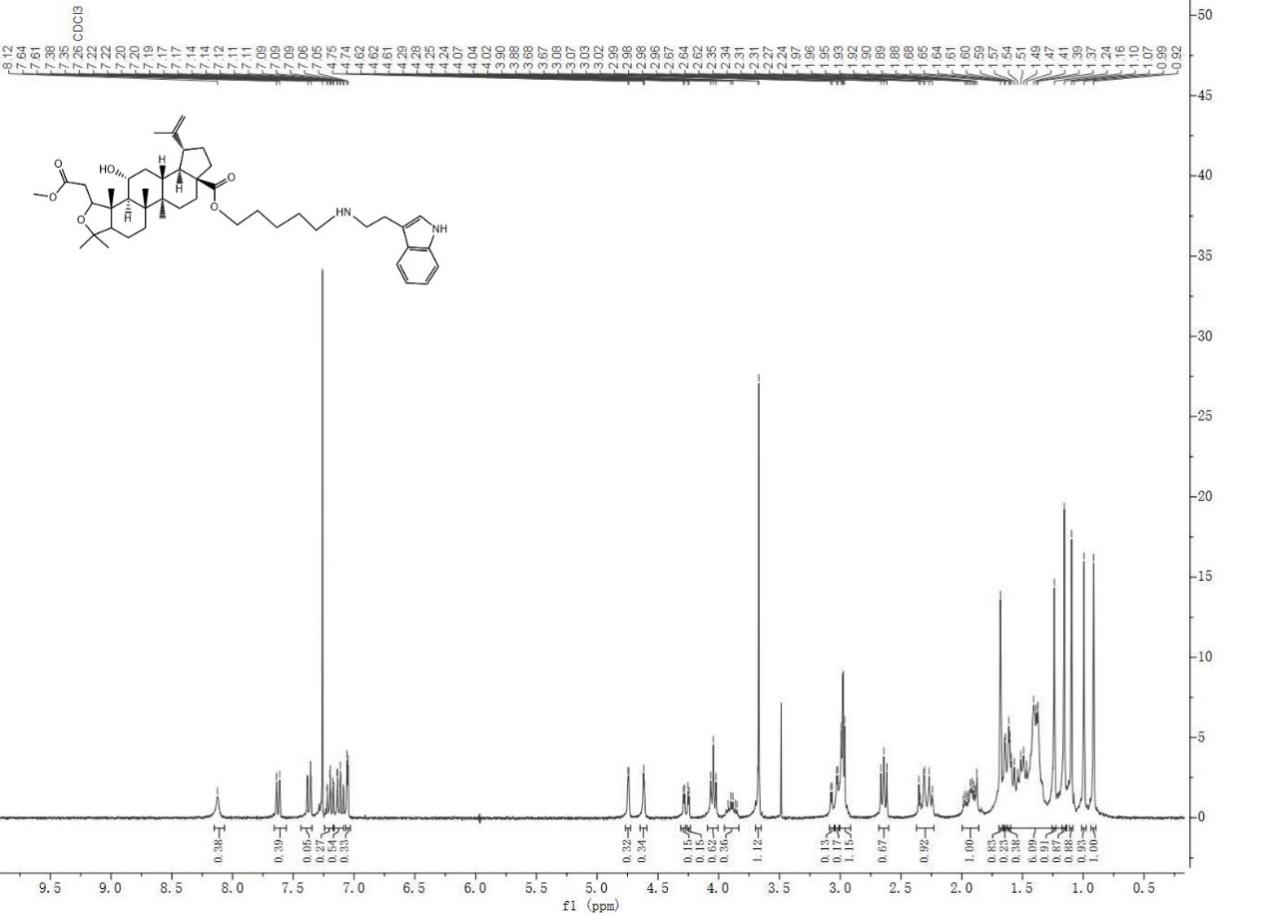


Figure S31. *HPLC tracing of compound* ***72****.*


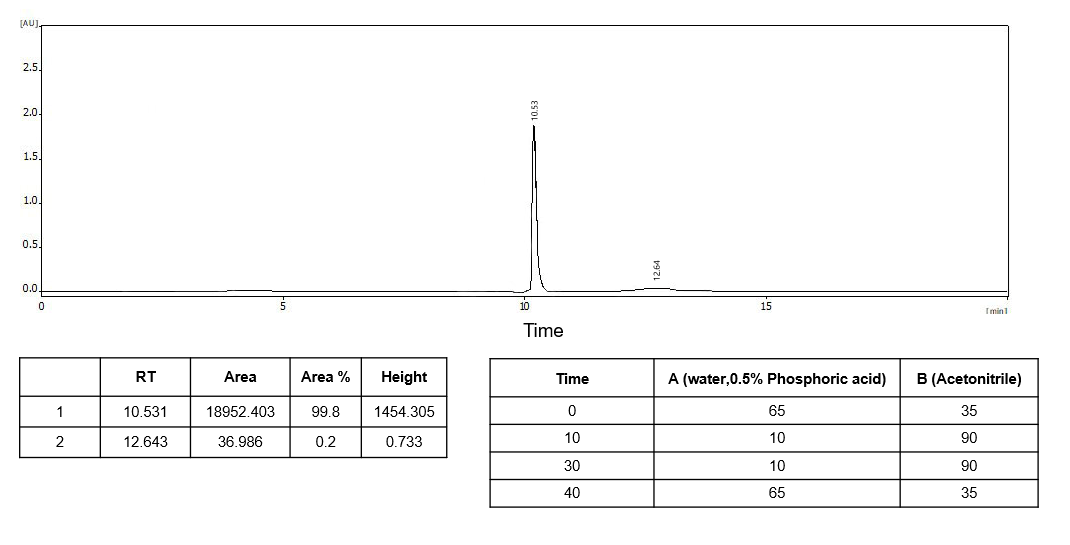


Figures S32. *^13^C and ^1^H NMR of compound* ***72****.*


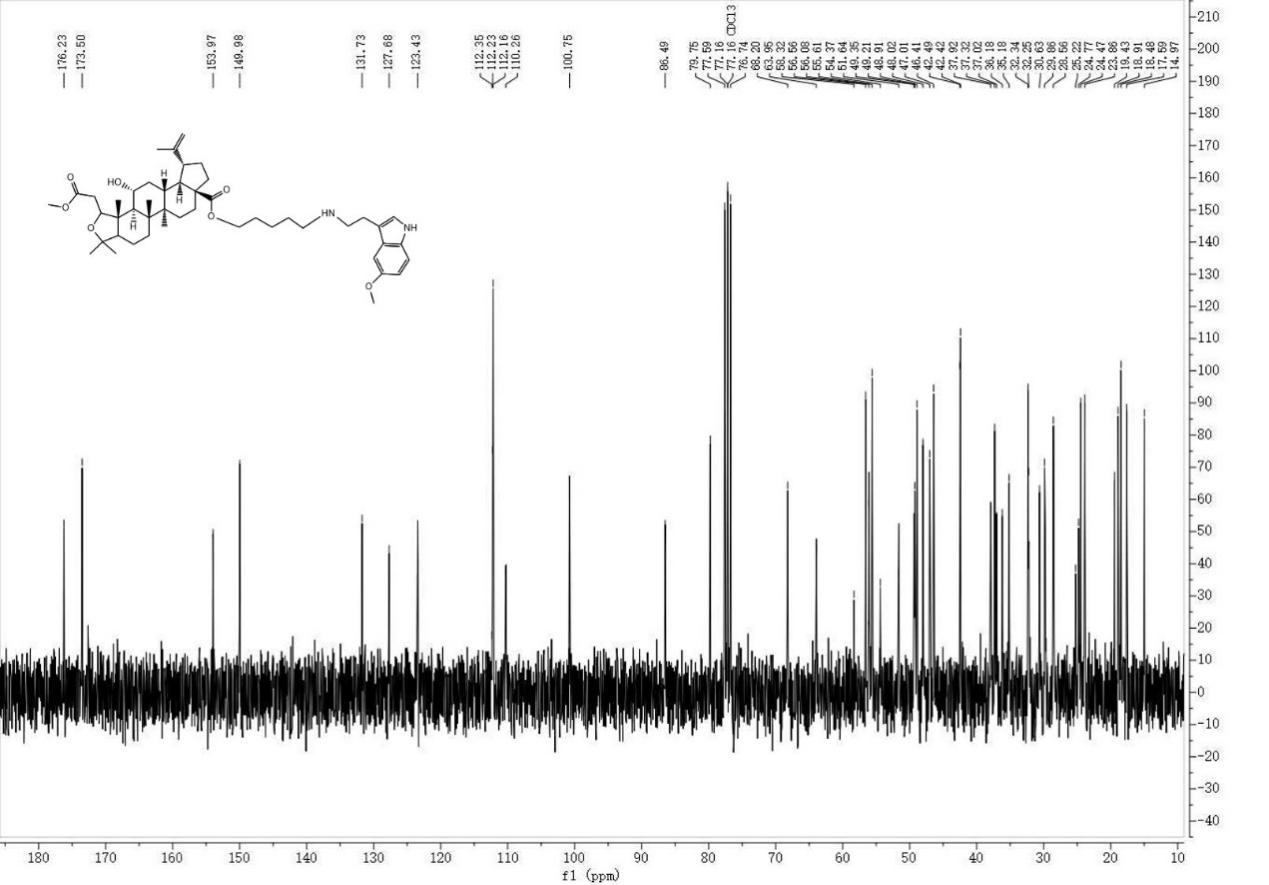

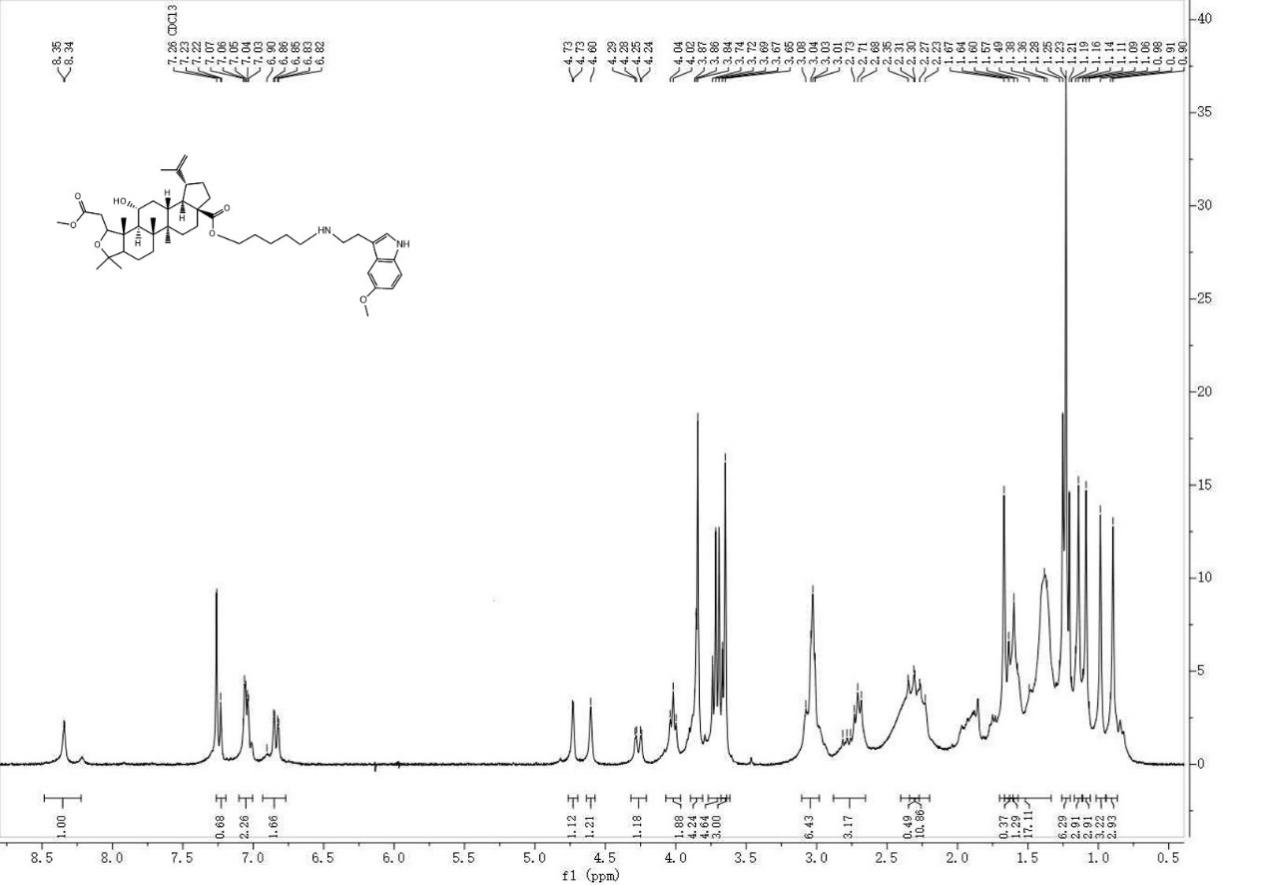


Figure S33. *HPLC tracing of compound* ***80****.*


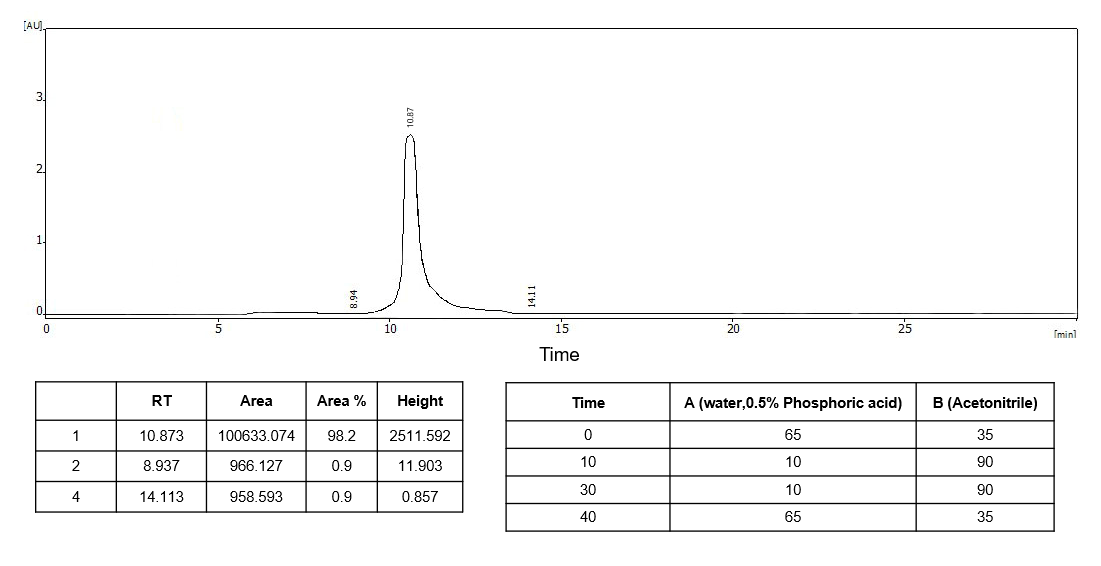


Figures S34. *^13^C and ^1^H NMR of compound* ***80****.*


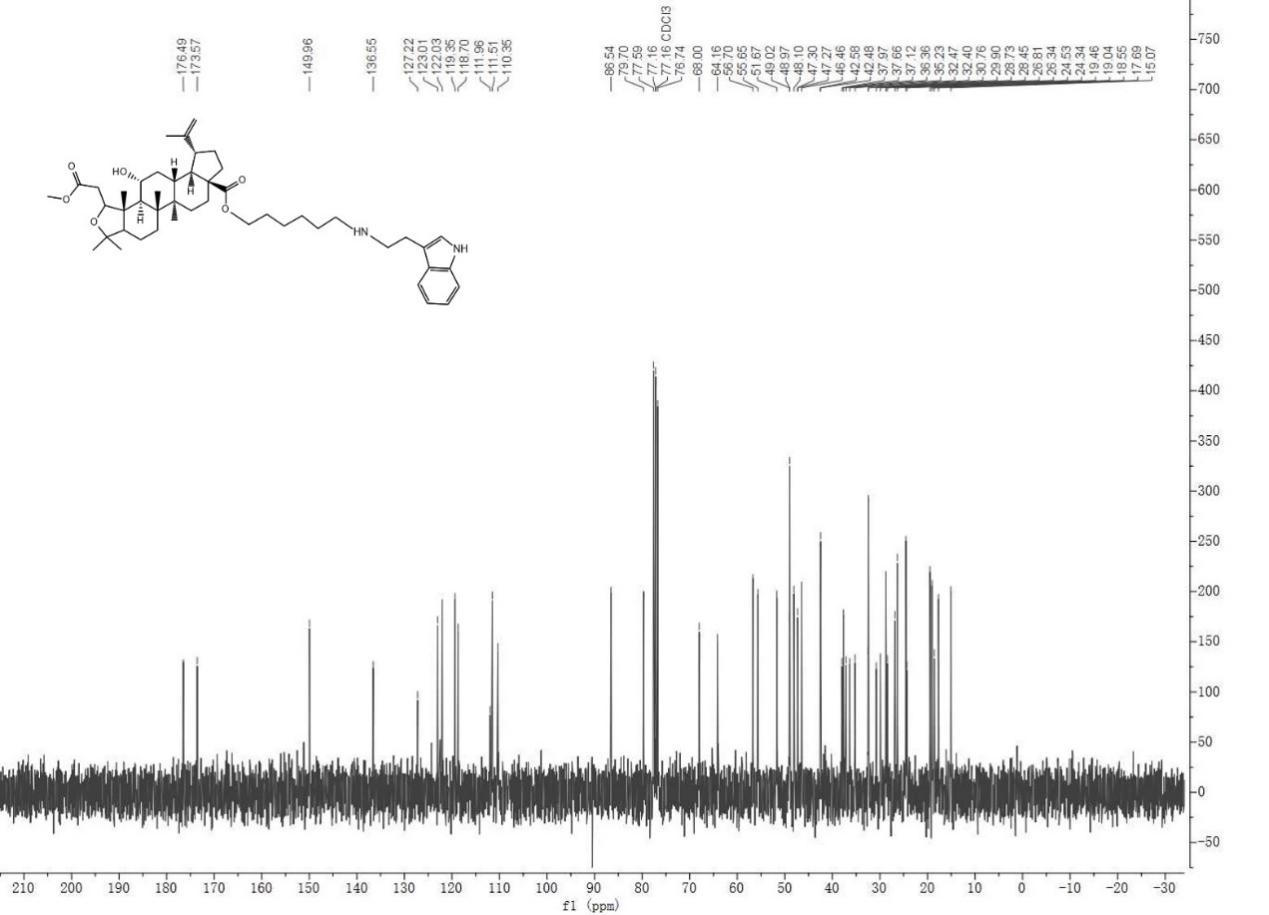

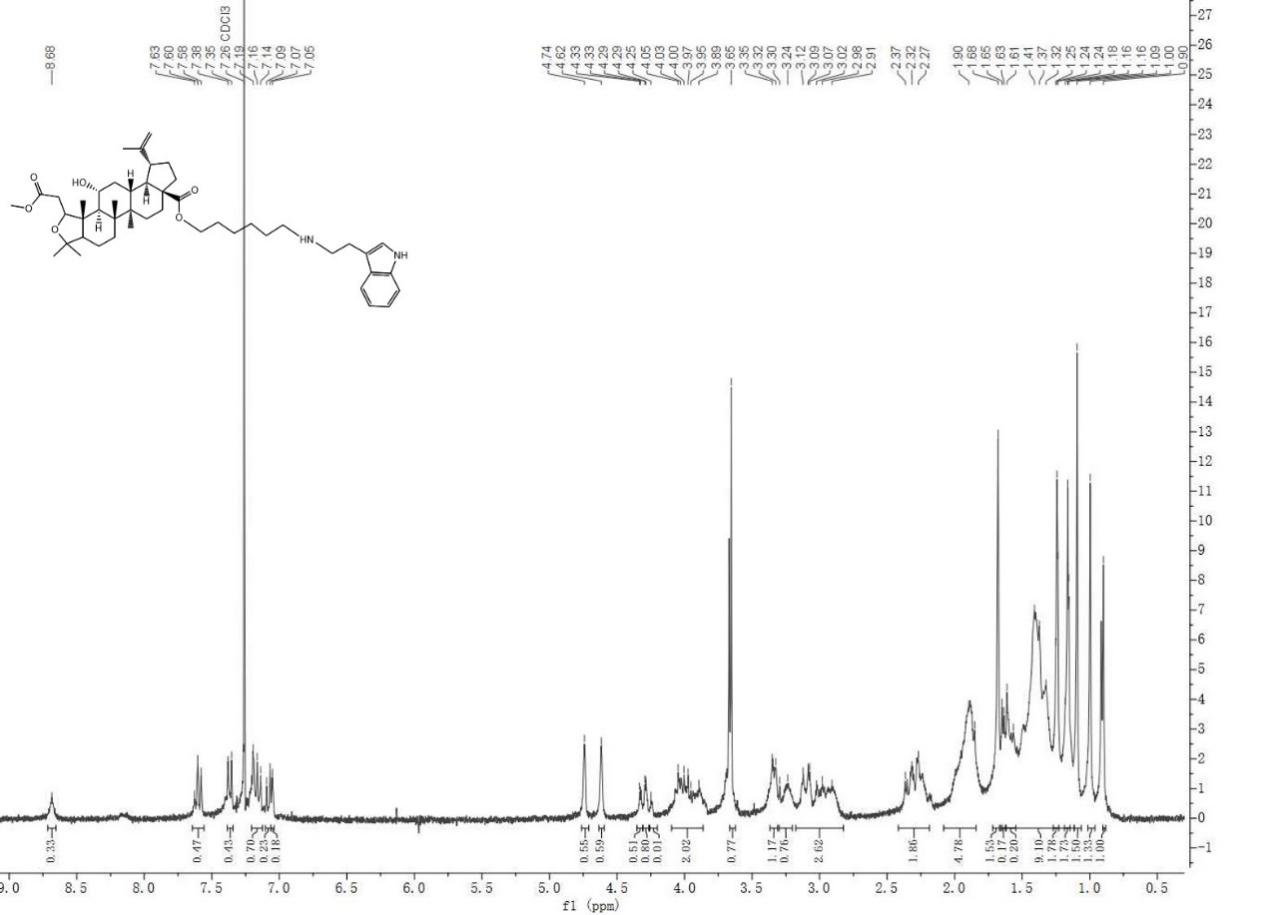


Figure S35. *HPLC tracing of compound* ***81****.*


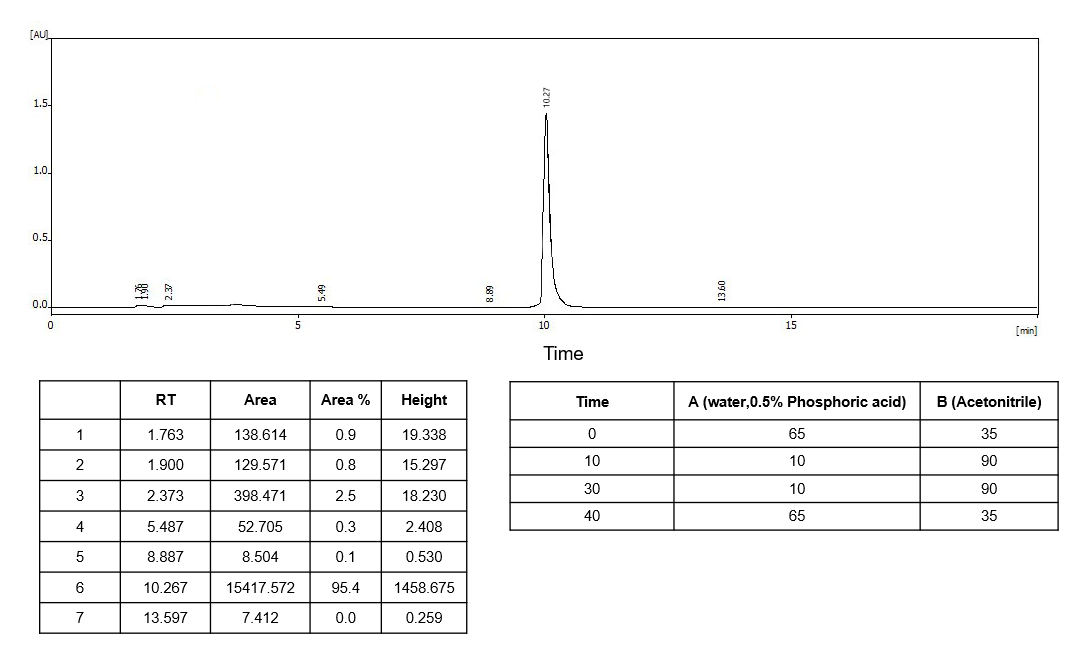


Figures S36. *^13^C and ^1^H NMR of compound* ***81****.*


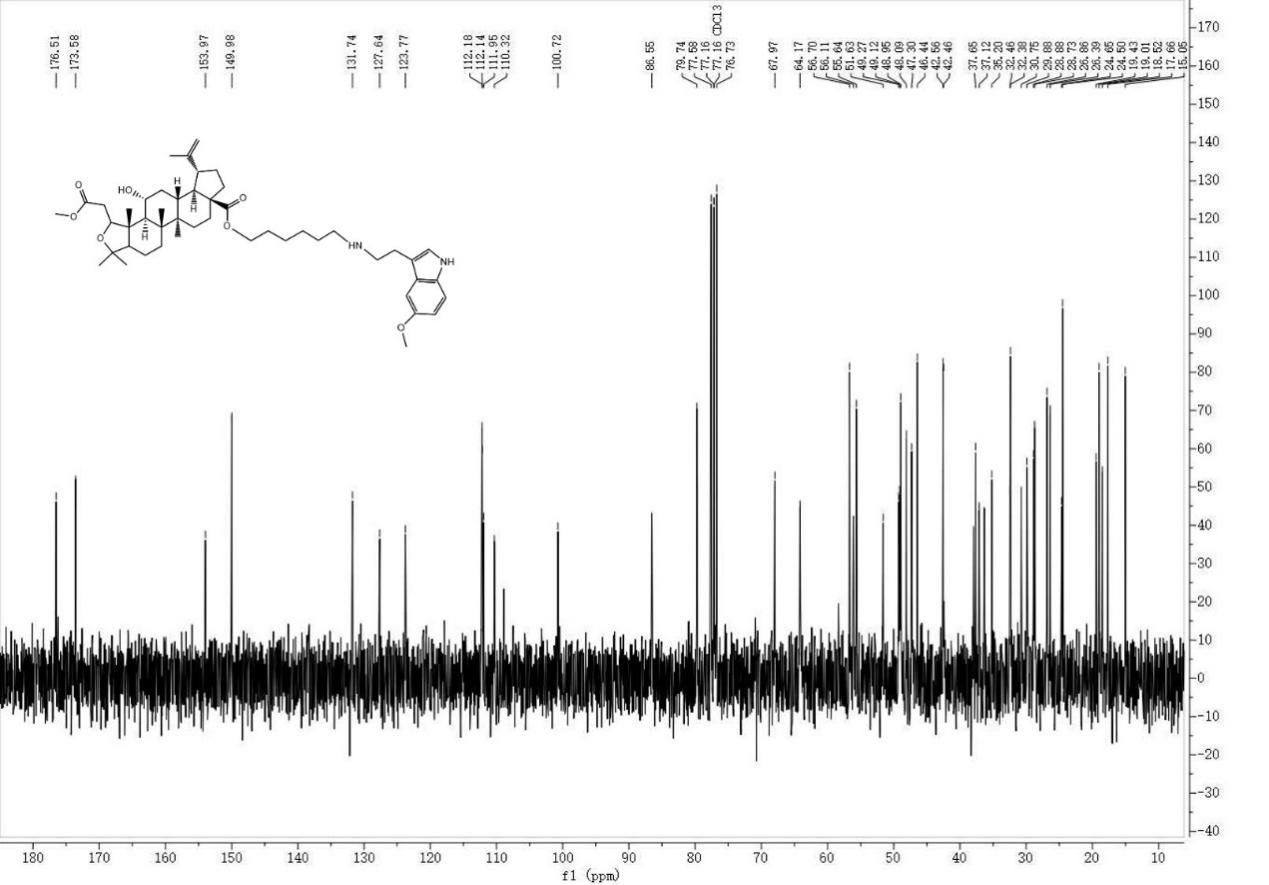

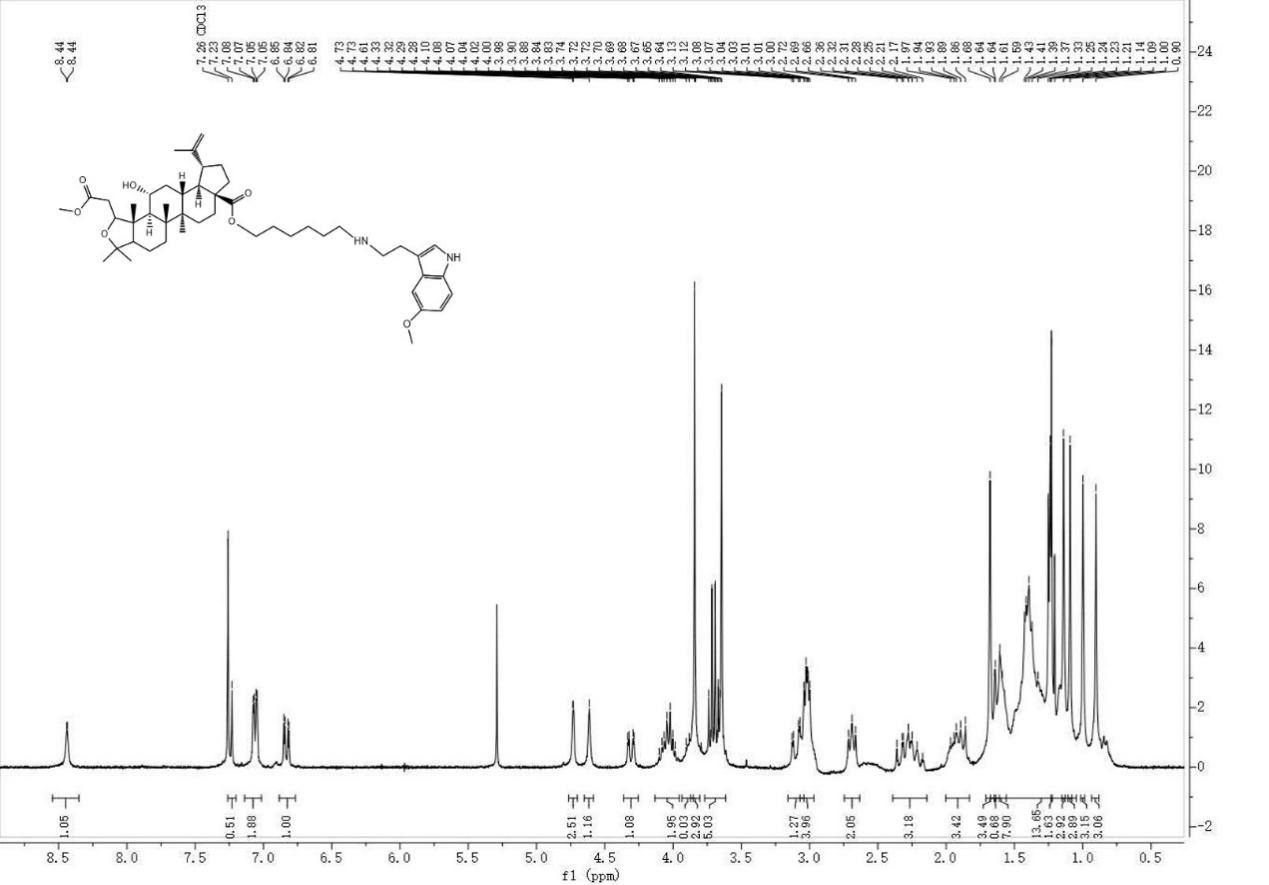


Figure S37. *HPLC tracing of compound* ***89****.*


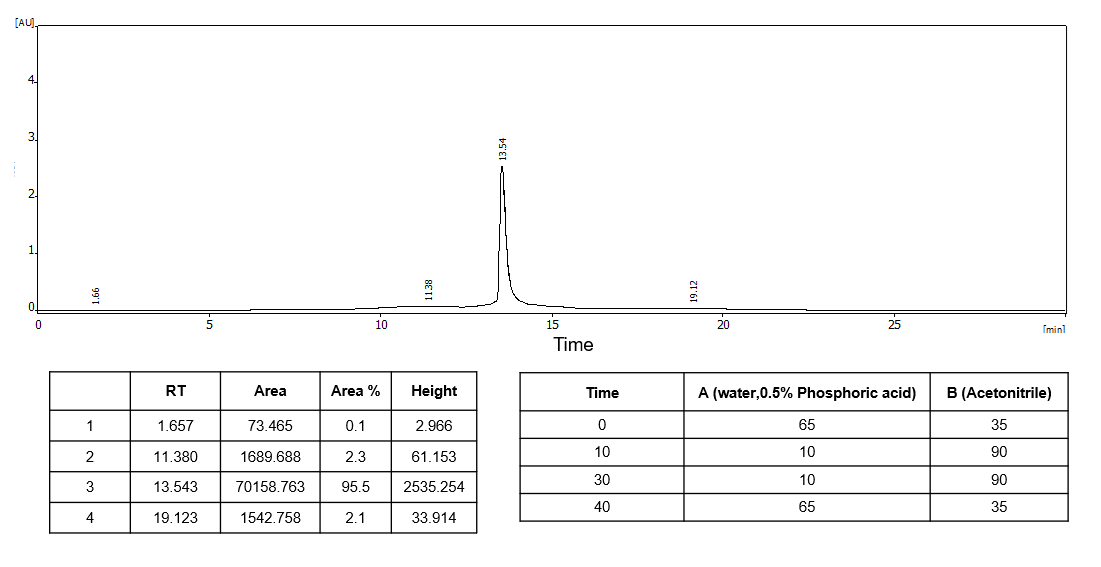


Figures S38. *^13^C and ^1^H NMR of compound* ***89****.*


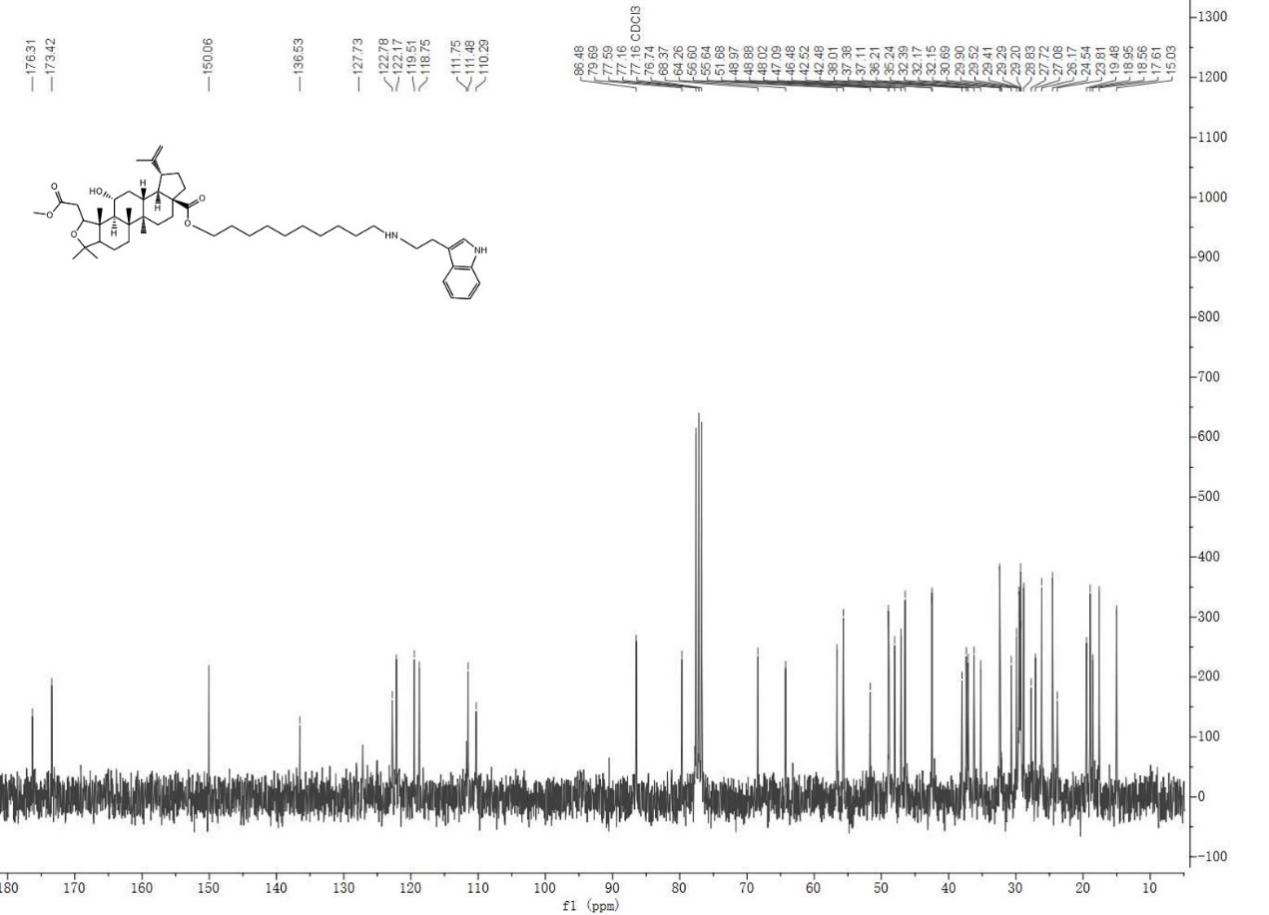

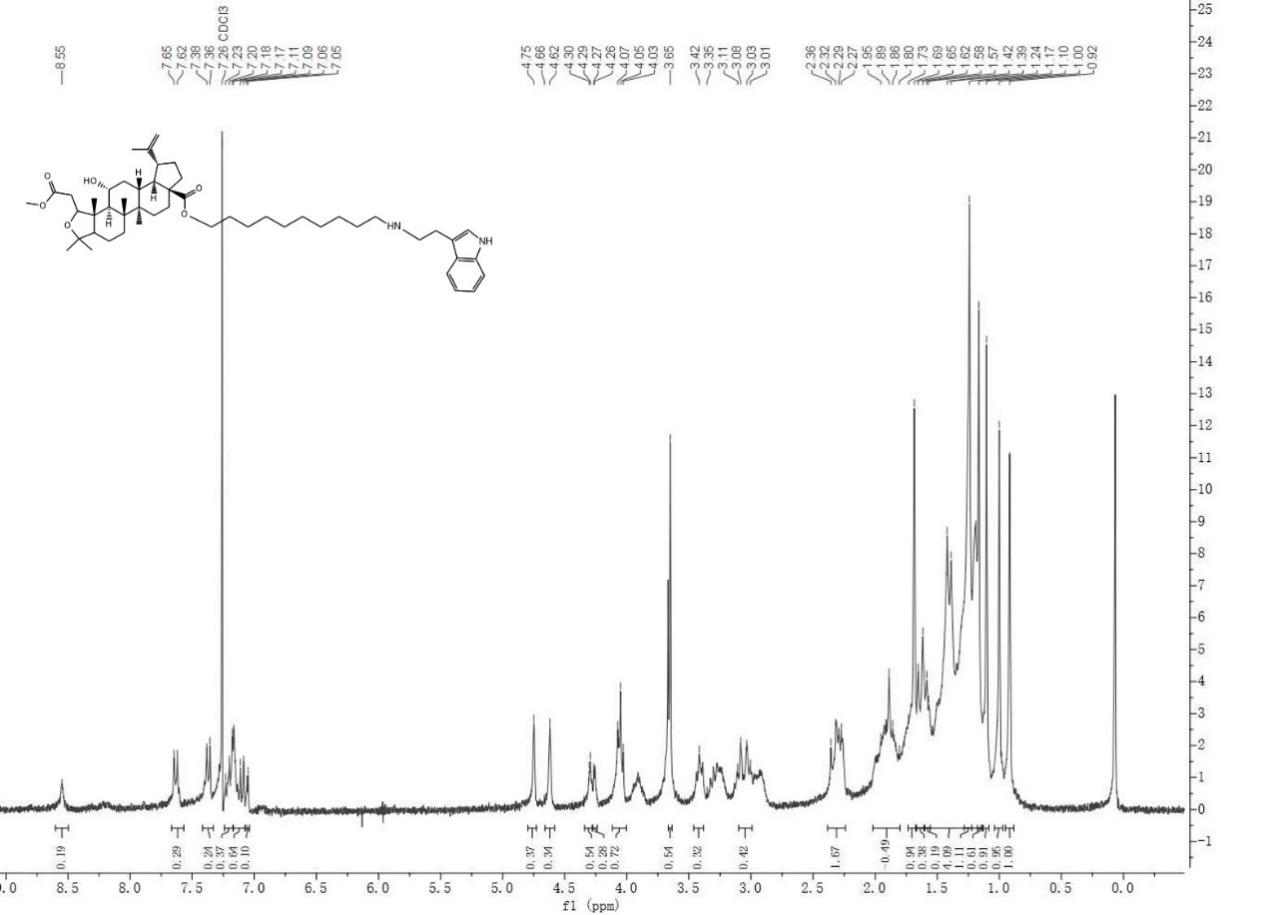


Figure S39. *HPLC tracing of compound* ***90****.*


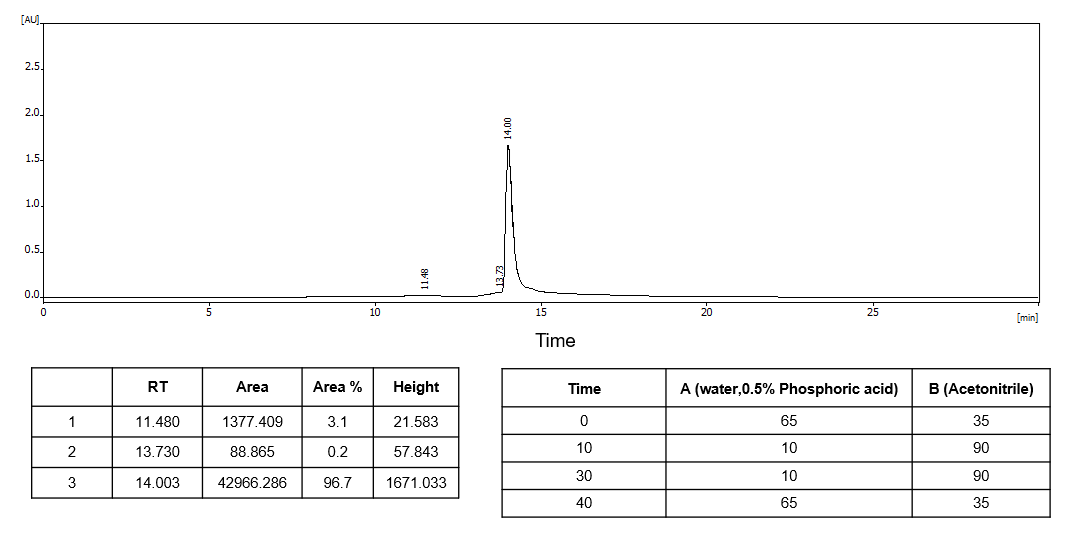


Figures S40. *^13^C and ^1^H NMR of compound* ***90****.*


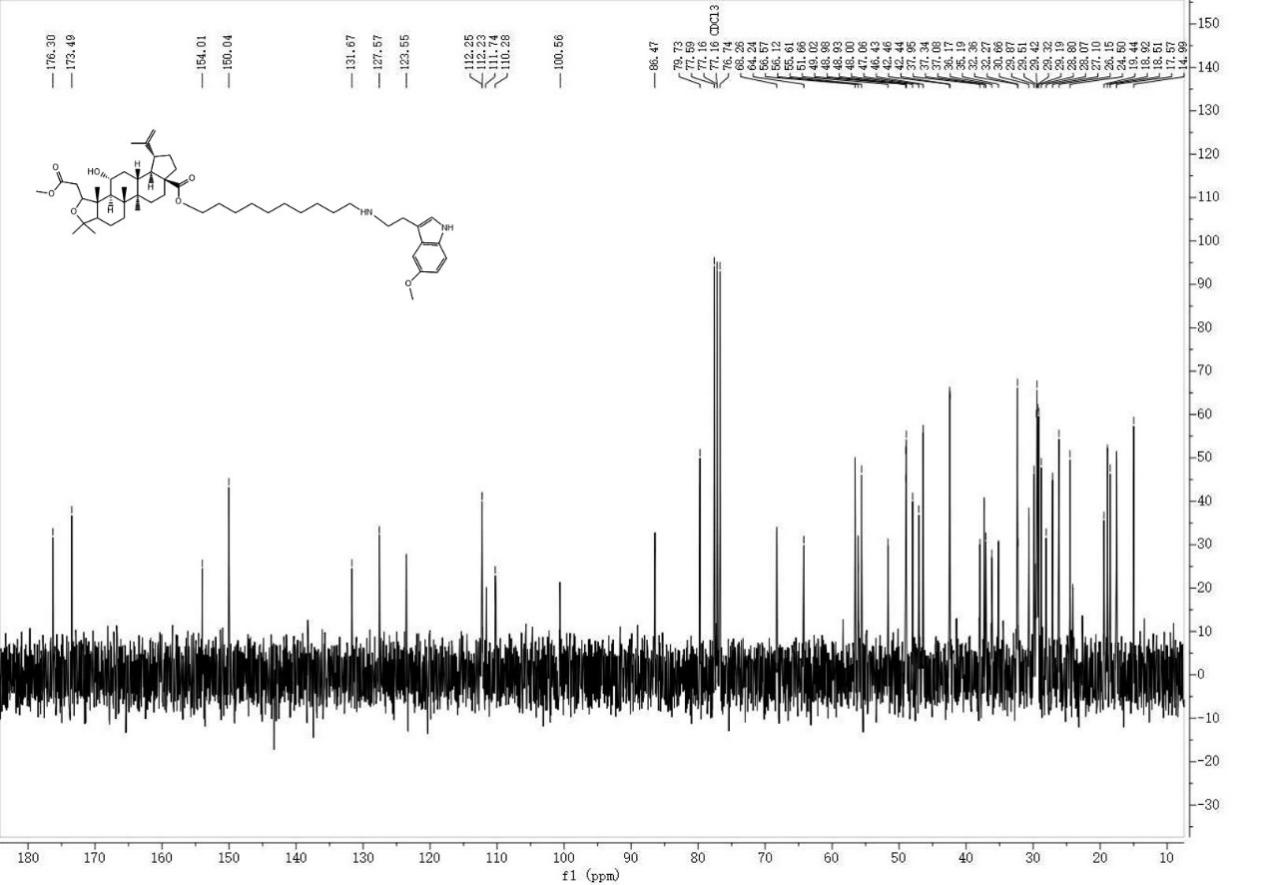

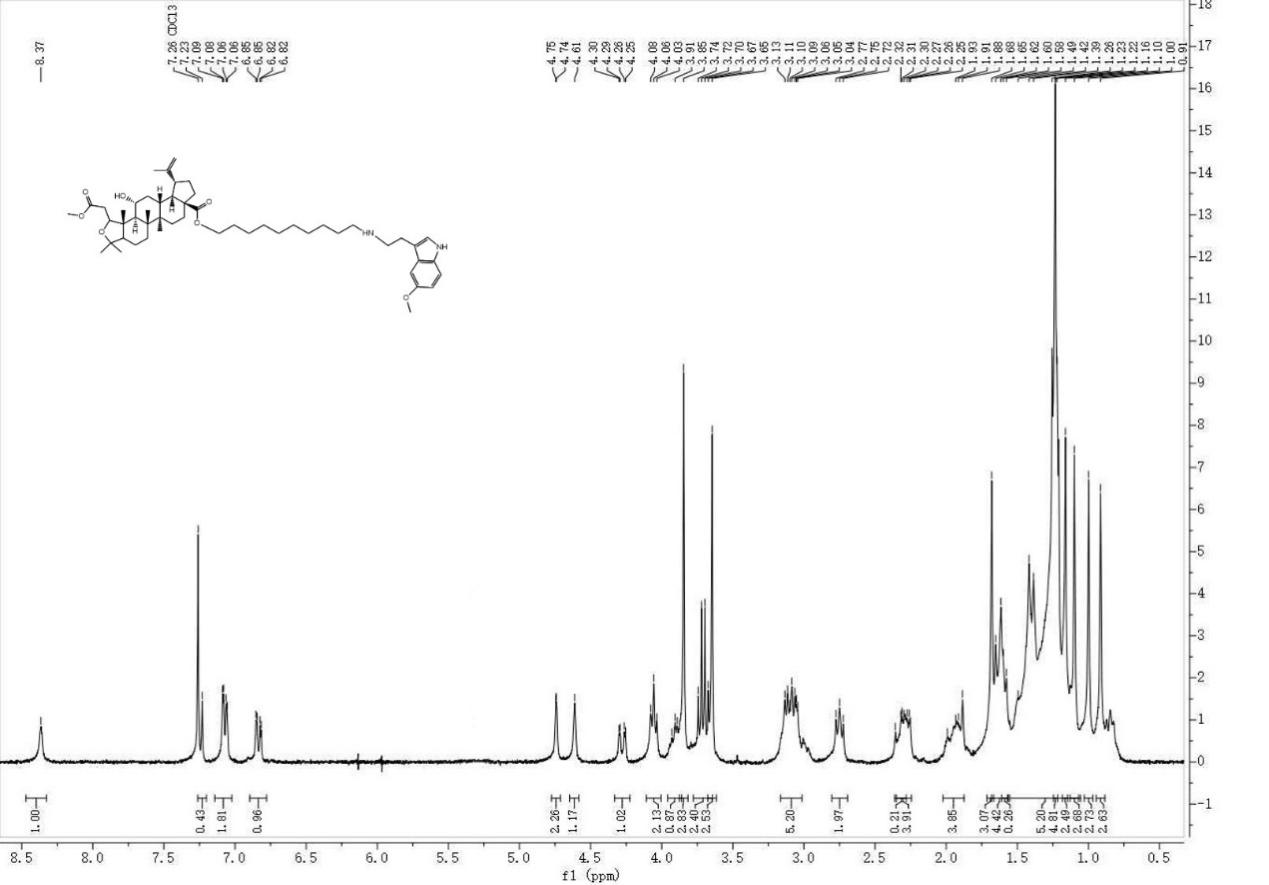

Supplement: Supplementary file 1 — Supplementary Information. [file 41598_2025_4855_MOESM1_ESM.docx]
